# Fast and accurate prediction of the regioselectivity of electrophilic aromatic substitution reactions†
†Electronic supplementary information (ESI) available. See DOI: 10.1039/c7sc04156j


**DOI:** 10.1039/c7sc04156j

**Published:** 2017-11-13

**Authors:** Jimmy C. Kromann, Jan H. Jensen, Monika Kruszyk, Mikkel Jessing, Morten Jørgensen

**Affiliations:** a Department of Chemistry , University of Copenhagen , Copenhagen , Denmark . Email: jhjensen@chem.ku.dk ; http://www.twitter.com/janhjensen; b Discovery Chemistry , DMPK , Neuroscience Drug Discovery , H. Lundbeck A/S, Valby , Denmark . Email: mojj@lundbeck.com; c Department of Drug Design and Pharmacology , University of Copenhagen , Copenhagen , Denmark

## Abstract

While computational prediction of chemical reactivity is possible it usually requires expert knowledge and there are relatively few computational tools that can be used by a bench chemist to help guide synthesis. The RegioSQM method for predicting the regioselectivity of electrophilic aromatic substitution reactions of heteroaromatic systems is presented in this paper. RegioSQM protonates all aromatic C–H carbon atoms and identifies those with the lowest free energies in chloroform using the PM3 semiempirical method as the most nucleophilic center. These positions are found to correlate qualitatively with the regiochemical outcome in a retrospective analysis of 96% of more than 525 literature examples of electrophilic aromatic halogenation reactions. The method is automated and requires only a SMILES string of the molecule of interest, which can easily be generated using chemical drawing programs such as ChemDraw. The computational cost is 1–10 minutes per molecule depending on size, using relatively modest computational resources and the method is freely available *via* a web server at ; http://www.regiosqm.org. RegioSQM should therefore be of practical use in the planning of organic synthesis.

## Abstract 2

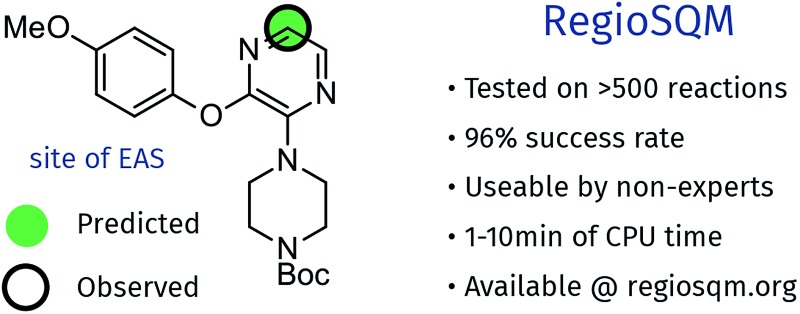
A fast and user-friendly computational for predicting the regioselectivity of electrophilic aromatic substitution reactions of heteroaromatic systems is presented.

## Introduction

Heteroaromatics and benzene derivatives constitute important structural classes in drug discovery, agrochemistry, and material science. Their halogenated derivatives are often applied as substrates in carbon–carbon and carbon–heteroatom cross-coupling reactions such as Suzuki–Miyaura, Heck, and Buchwald–Hartwig couplings.^1^,^2^ The prerequisite (hetero)aryl halides are typically prepared by electrophilic aromatic substitution (EAS).^3^ Halogenated arenes are also important substrates for directed metallation reactions and for the generation of organolithium and organomagnesium species by metal–halogen exchange, metal insertion, or direct metallation.^4^ Unlike the benzene series for which the relative reactivity of the substrates is textbook knowledge, it is not *a priori* obvious at which position(s) halogenation will occur for heteroaromatic systems, especially in compounds that contain multiple (hetero)aromatic rings or in compounds that contain both heteroarene and benzene rings. Consequently, organic chemists tend to install the halogens early in the synthesis because they are not comfortable with late-stage functionalization.^5^ Thus, there is an unmet need for synthetic chemists to be able to predict the regioselectivity of EAS reactions. It is critical that prospective methods are easy for the end-users to work with to have a significant impact. This paper provides a fast, reliable, and easy-to-use computational tool to predict the site selectivity of EAS reactions more robustly than our previously reported NMR-based method.^6^ In a broader sense, the ability to block the more reactive site(s) in complex heteroaromatics may increase the use of halogens as protective groups in arene chemistry.^7^,^8^

It was recently reported that empirically calculated ^1^H and ^13^C chemical shifts can be applied to retrospectively account for the regiochemical outcome of *ca.* 80% of 130 EAS reactions from the literature. For many of the remaining cases, the regioselectivity was rationalized by visual inspection of the HOMO orbital computed using DFT, bringing the success rate up to *ca.* 95%.^6^ The chemical shifts were obtained immediately from the structure using ChemDraw. Conversely, DFT calculations are time-consuming for larger compounds and cannot be performed by non-expert users and there is a certain degree of subjectiveness in predicting the regioselectivity based on the HOMO orbitals. Finally, the sound judgment of chemists was required to evaluate the reactivity of unsubstituted and electron-deficient benzenes, and the method failed for 1,2,4-triazoles.

**Scheme 1:**
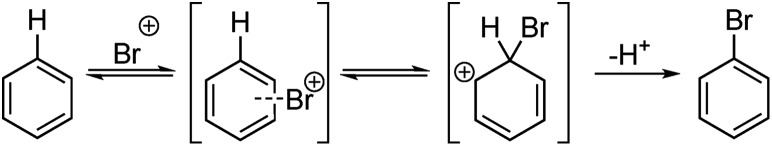
General EAS bromination mechanism.

In this paper the Kruszyk *et al.*^6^ study is extended to more than 525 reactions reported in the literature and we present a new semiempirical quantum mechanical (SQM)-based method that improves both the accuracy and precision of the predictions. The majority of the selected reactions were performed with *N*-bromosuccinimide (NBS) to avoid any substrate protonation that might occur when using for example bromine in acetic acid. This precaution may not have been necessary as the vast majority of cases where both conditions have been applied led to the same regiochemical outcome of the halogenation reaction. The computational work is based on the observation by Streitwieser and others that the rates of many EAS reactions correlate well with equilibrium values for protonation in solution.^9^ This observation implies that the site with the highest proton affinity (*i.e.* the protonated regioisomer with the lowest free energy) corresponds to the most probable site for EAS, as was demonstrated by Wang and Streitwieser for several polycyclic aromatic hydrocarbons.^10^ This approach is in line with the commonly accepted EAS reaction mechanism when considering the protonated species as a “surrogate” for the arenium ion (Scheme 1). The rationalization of the regioselectivities in the benzene series based on the relative stabilities of the possible sigma complexes is textbook knowledge and Galabov and co-workers^11^,^12^ electrophile have shown that the computed binding energy of the Bromine cation is correlated with the experimentally measured partial rate factors. Jensen and co-workers recently showed that SQM methods can be used to accurately predict p*K*_a_ values of ionizable groups.^13^,^14^ Gratifyingly, with only the SMILES string as input^15^ this approach pin-points the protonated regioisomer(s) with lowest energy and, hence, the most likely EAS reaction site(s).

**Fig. 1:**
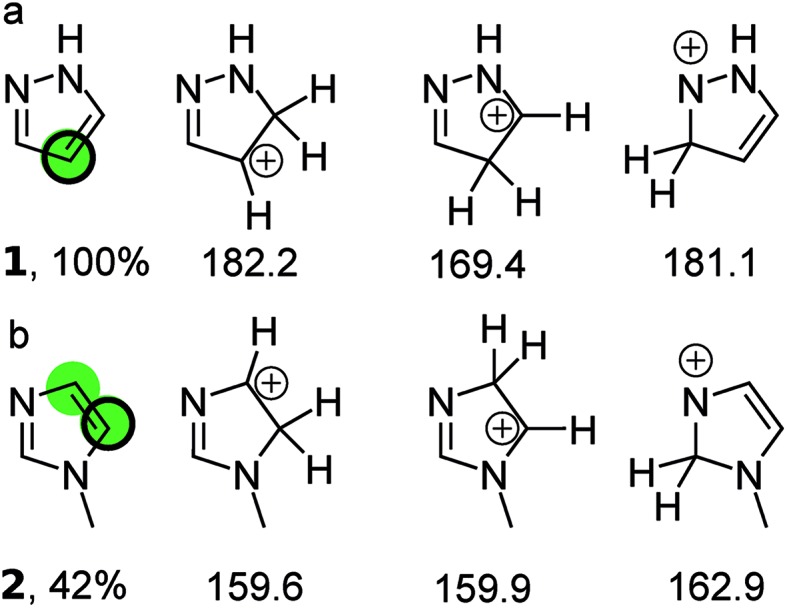
(a) The three protonated isomers of pyrazole and their PM3 standard free energies (kcal mol^–1^) in chloroform. The protonated carbon in the isomer with the lowest free energy (169.4 kcal mol^–1^) is taken to be the bromination site in the parent compound (green circle) and corresponds to the quantitative yield obtained with NBS (black ring). (b) The three protonated isomers of *N*-methylimidazole and their PM3 free energies (kcal mol^–1^) in chloroform. The middle isomer has a standard free energy that is within 1 kcal mol^–1^ of the left isomer, which has the lowest standard free energy. Therefore both protonated carbon atom are taken as most likely bromination sites in the parent compound (green circles). This is considered a correct prediction because one of the sites corresponds to that observed experimentally with NBS (black ring). The references to the experimental data can be found in ESI.†

Fig. 1 illustrates the principle for pyrazole and *N*-methylimidazole. For pyrazole the predictions point exclusively to the 4-position; indeed literature examples of the other regioisomer being formed were not identified. The situation is more complex for *N*-methylimidazole with both 4 and 5 positions as potential sites for the reaction. This correlates well with the moderate yield of 42%. Indeed the 4,5-dihalogenated adduct can be obtained in synthetically useful yields.^16^–^18^ In fact all three positions in *N*-methylimidazole can be halogenated which is not surprising given the fact the calculations suggest that the three possible arenium ions have similar standard free energies.^19^–^21^ Finally, a 1 : 1 mixture of the 4- and 5-brominated products has been reported for *N*-benzylimidazole, which is in line with the predictions.^22^,^23^

## Computational methodology

We used 118 reactions reported by Kruszyk *et al.*^6^ to identify the best conditions for the SQM calculations. Subsequently this method was applied to the entire dataset, and all results are compiled in the ESI.† The most likely site for electrophilic substitution was predicted by finding the protonated regioisomer with the lowest standard free energy (Fig. 1) computed as the sum of the semiempirical heat of formation and the solvation free energy1
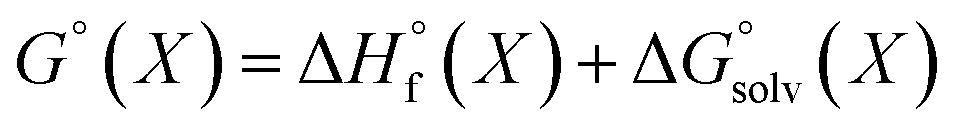



All energy terms are computed using solution phase geometries unless noted otherwise. 
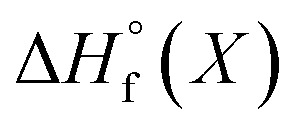
 is computed using either PM6-DH+,^24^ PM6,^25^ PM7,^26^ PM3,^27^ or AM1,^28^ while 
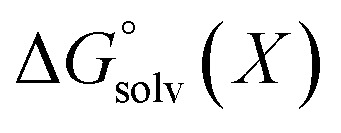
 is computed with the COSMO^29^ solvation method using MOPAC2016. A maximum of 200 optimization cycles were used for geometry optimizations. The conformers for each protonated isomer were generated from SMILES strings using RDKit.^30^ The number of conformers is min(1 + 3*n*_rot_, 20) where *n*_rot_ is the number of rotatable bonds. Increasing 20 to 50 has no discernible effect on the accuracy as shown in ESI.† Gas phase PM3 calculations were complemented with the prototypical solvents used in EAS chemistry, chloroform (dielectric = 4.8) and *N*,*N*-dimethylformamide (DMF, dielectric = 37.0). Structures where protons have transferred and/or other bonds were broken are removed from the analysis. Gas phase, chloroform, and DMF were evaluated using PM3 and the COSMO solvation model in MOPAC. PM3/COSMO was chosen because it gave the best results in a previous study of amine p*K*_a_ values.^14^ It is important to include solvent as gas phase calculations gave incorrect predictions for eleven cases *versus* two cases for chloroform and DMF, respectively. Predictions with chloroform as a solvent resulted in six to eight incorrect predictions using PM6 and PM7 or AM1 and PM6-DH+ (see ESI†). In summary, PM3/COSMO/chloroform or DMF gave the most correct predictions and further work was limited to PM3/COSMO/chloroform henceforth and referred to as RegioSQM.

## Results and discussion

**Fig. 2:**
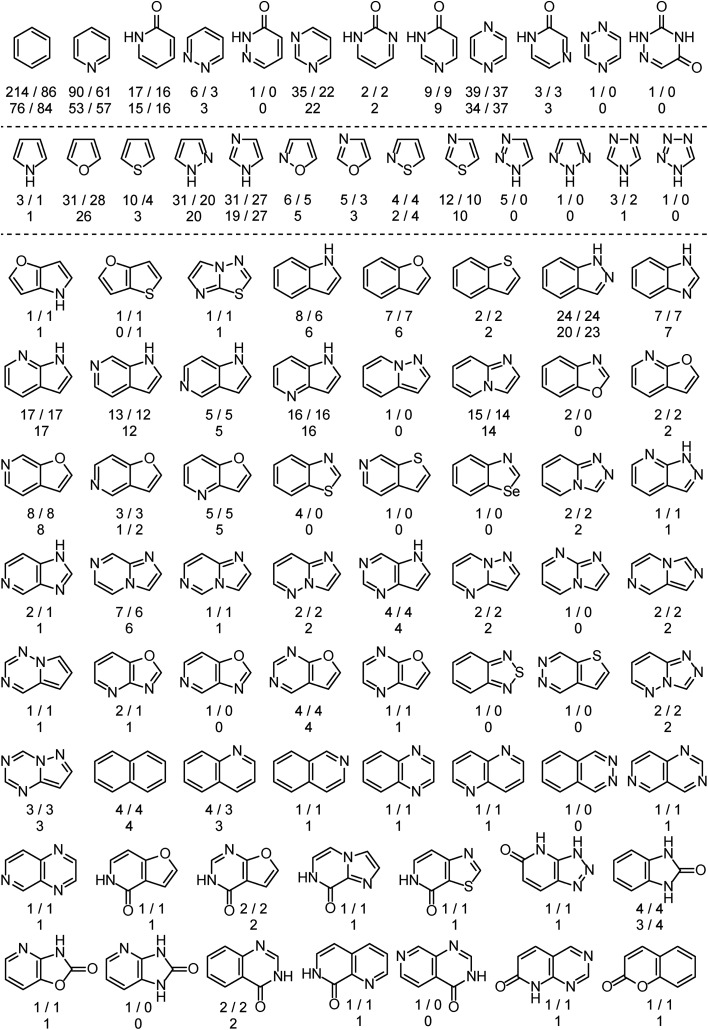
Overview of the mono- and bicyclic heteroaromatics in the analysis. There are 214 benzene containing compounds in the dataset, 86 of which undergo the halogenation reaction at the benzene ring; 76 and 84 of these reactions are correctly predicted by RegioSQM using a 1.0 and 3.0 kcal mol^–1^ cutoff, respectively. If increasing the cutoff to 3 kcal mol^–1^ does not identify additional reactive sites then only the number of correct predictions with 1 kcal mol^–1^ is provided. The references to the experimental data can be found in ESI.†

The analysis is based on more than 525 EAS reactions compiled from the literature. RegioSQM can rationalize the regiochemical outcome of 90% and 96% of these when using a 1.0 and 3.0 kcal mol^–1^ cutoff, respectively. The substrates contain a total of almost 900 aromatic rings as summarized in Fig. 2. The dataset includes twenty monocyclic systems ranging from pyrrole to 1,2,4-triazine-3,5(2*H*,4*H*)-dione and 64 bicyclic systems. Important aromatic systems like benzene and pyridine as well as indazole and 7-azaindole are well-represented with 16–214 examples, but the analysis also includes a number of less common heteroaromatics like pyridazin-3(2*H*)-one and imidazo[1,2-*a*]pyrimidine with **1** and **2** examples, respectively. This diversity of (hetero)aromatic cores serves to illustrate the applicability of RegioSQM as a predictive tool to guide organic synthesis. The full dataset is provided in the ESI† where the compounds are grouped according to the reacting ring.

**Fig. 3:**
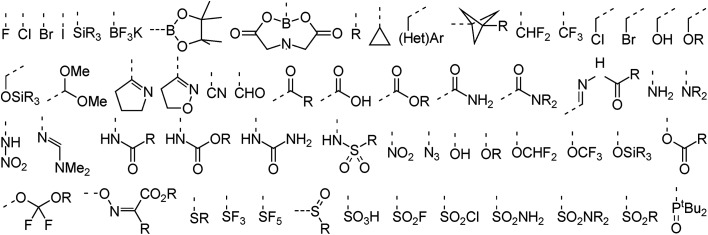
Selected functional groups bound directly to the reacting (hetero)aromatic rings.

In terms of functional groups attached directly to reacting aromatic rings the analysis covers more than 50 substituents that range from the four halogens to more exotic functionalities such as propellanes, imines, azides, and trifluoroborates. A subset of these groups is provided in Fig. 3. An overview of functionalities present elsewhere in the dataset is provided in the ESI (Fig. S1†). These include common amine protective groups like *tert*-butyl oxo-carbonyl (Boc), benzyl oxo-carbonyl (CBz) and tosyl/nosyl sulfonamides as well as synthetically useful functional groups like silyl ethers, amides, esters and lactones, succinate esters (HOSU-esters), alkynes and olefins, aldehydes, ketones, and ketals. The set also covers conjugated epoxides and acrylonitriles, Weinreb amides, enol ethers, primary alkyl and benzyl halides, phosphonates, and unprotected catechols. Indeed, the dataset was assembled to cover not only a diverse set of ring systems but also a wide range of typical as well as less common functional groups to demonstrate the usefulness of RegioSQM as a synthesis planing tool.

**Fig. 4:**
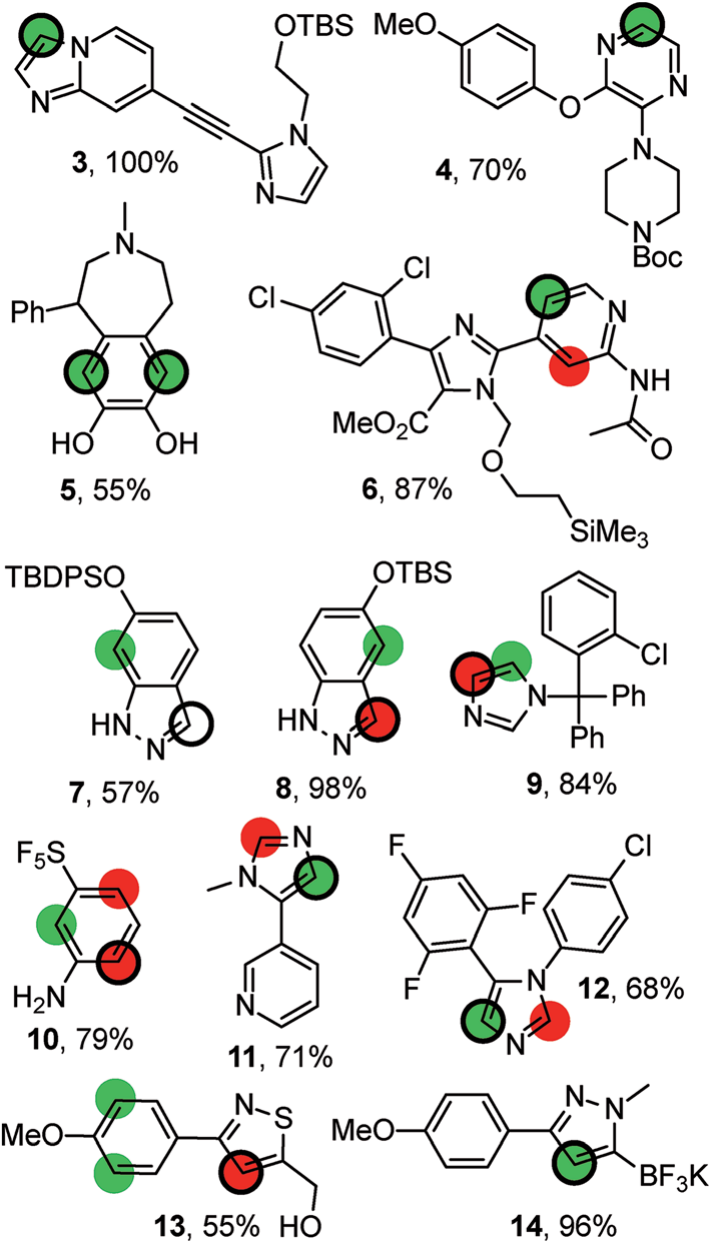
Representative examples from the dataset. The reaction were performed with NBS (**4**, **6**, **9**, **10**, **11**), NCS (**13**, **7**, **12**, **14**), NIS (**3**, **8**), or SO_2_Cl_2_ in acetic acid (**5**). The green and red circles denote sites with standard free energies below 1 kcal mol^–1^ and 3 kcal mol^–1^, respectively. The site of reaction is indicated by the black ring. The references to the experimental data can be found in ESI.†

Fig. 4 compiles possible outcomes of the predictions made by RegioSQM. Firstly, compounds **3**, **4**, **6**, **11**, and **12** are examples where only a single position is proposed at 1 kcal mol^–1^ leading to correct predictions (this applies to *ca.* 400 compounds in the dataset). Similarly there are *ca.* 40 cases including **5** with two proposed sites at 1 kcal mol^–1^ and where the corresponding bis-halogenated products were obtained. The incorrect prediction for **7** may reflect sterical hindrance due to *tert*-butyl-diphenylsilyl group or be a consequence of deprotonation of indazole under the somewhat unusual basic reaction conditions (KO^*t*^Bu/*N*-chlorosuccinimide (NCS)). The corresponding *tert*-butyl-dimethylsilyl analog **8** illustrates that the 3-position of this ring system is also a reactive site. Furthermore, chlorination and nitration of 6-hydroxy-indazole occur at the 7-position in agreement with the predictions.^31^ Steric effects may also explain the experimental data for **9** and **10**. Conversely, it is difficult to rationalize the regioselectivity for **13** although a directing effect of the primary alcohol cannot be ruled out. Imidazole **12** is reported to react with NCS at the 4-position in agreement with the predictions. However, bromination of the same substrate with Br_2_ has been reported to occur at the 2-positions of the imidazole ring.^32^ RegioSQM is also applicable to Molander's potassium trifluoroborates as illustrated for **14**.^33^,^34^

Broad functional group compatibility (Fig. 3) is important when considering the potential of using halogen substituents not only as chemical handles for further derivatization but also as orthogonal protective groups in complex molecule synthesis. With the ability to predict the regiochemical outcome of EAS chemistry chemists could focus on synthesis of key scaffolds from which substituents could be installed as late as possible, for example in the context of exploring Structure–Activity-Relationships (SAR) or to support large scale preparation of one of more analogs from a common late-stage intermediate.

## Practical usage considerations

RegioSQM consists of several python scripts executed using two Linux Bash scripts, one that automatically generates all MOPAC input files given a list of SMILES strings and one that extracts energies from the MOPAC output files, identifies the most reactive arene atom(s), and generates a 2D representation of the molecule and highlights the likely site of reaction (see ESI† for examples). All scripts are made available on GitHub under the MIT open source license (see ESI† for details). The SMILES strings for all molecules are provided in ESI† and the results can thus be reproduced by installing the necessary software and running the two scripts. RegioSQM users need to install RDKit, OpenBabel,^35^ and MOPAC2016 on a Linux computer or partition. RDKit and OpenBabel are open source packages, while MOPAC2016 is freely available to academic researchers.

The computational cost per molecule depends on the size of the molecule and the number of energy minimizations, where the latter is a function of the number of rotatable bonds and the number of protonated isomers for a given molecule. The prediction of the most reactive site of a relatively small molecule like 3-(1-methyl-1*H*-imidazol-5-yl)pyridine (**11**) requires 2–3 minutes on a single CPU. A relatively large and flexible molecule like **6** requires 45–60 minutes on a single CPU. Each energy minimization is run on a single CPU, so the computation time is reduced almost linearly with the number of CPUs. For example by using 24 CPUs the time requirement for **6** is reduced to 2–3 minutes. The computational cost could be further reduced by using Merck molecular force field (MMFF)^36^–^40^ energy minimized structures and PM3 single point energy calculations. However, when applied to the Kruszyk dataset^6^ this resulted in 22 incorrect predictions using chloroform as a solvent and a 1 kcal mol^–1^ energy cutoff.

## Conclusion and outlook

RegioSQM is a new computational method that identifies the aromatic carbon with highest proton affinity (Fig. 1) using the PM3 semiempirical method. It has been applied to a set of more than 525 EAS reactions compiled from the literature. With an energy cutoff of 1.0 kcal mol^–1^ to define sites with indistinguishable proton affinities (Fig. 1b), RegioSQM correctly predicts the mono-halogenation of 396 of these reactions and the bis-functionalization of an additional 39 compounds, leading to an overall success rate of 81%. This means that the false positive rate is 19%, *i.e.* that more sites are predicted to be reactive than are observed experimentally, using a 1 kcal mol^–1^ cutoff. The number of correct predictions increases 92% and 96% when considering examples with multiple predicted reactive sites as correct if the experimental site is among those identified by RegioSQM at energy cutoffs at 1 and 3.0 kcal mol^–1^, respectively. This increase in correct predictions comes at the expense of more incorrect predictions. RegioSQM fails to identify the experimental site of reaction in 13 cases (<3%).

The method is based on the MOPAC software package, which is free for academic research, and the RDKit and OpenBabel toolkits, both of which are open source. The scripts that automate the calculation and the associated python codes are available on GitHub under the MIT open source license (see ESI† for more details). The method is relatively fast, requiring on the order of 1–10 minutes per molecule depending on the size of the molecule and the number of CPUs available. RegioSQM should therefore be of practical use to synthetic chemists and is freely available as a web-service at ; http://www.regiosqm.org.

Unlike our previous NMR-based method,^6^ RegioSQM considers all aromatic CH positions. The software correctly predicts the low reactivity of fluorinated arenes and generally accounts for the high reactivity of 1,2,4-triazoles, both of which represent improvements over the original method. RegioSQM is fully automated and straightforward to use requiring only the SMILES string of the molecules of interest as input. Importantly, this means that neither computationally demanding density functional theory calculations, that are difficult to perform by non-expert users, nor visual inspection of the HOMO orbitals are required to obtain solid predictions regardless of the nature of the substrates. Finally, RegioSQM predicts the specific reactive sites correctly in 81% of the cases as compared to approximately 60% when basing predictions on the chemical shifts calculated using ChemDraw. RegioSQM is thus a much improved method compared to our NMR-based original approach.

The underlying dataset used to develop and test RegioSQM contains more than 80 different mono- and bicyclic aromatic cores bearing more than 50 functional groups directly on the rings that undergo the reaction as well as and more than 30 commonly used protective groups and important reactive functionalities in organic synthesis. This structural array not only illustrates the high chemoselectivity of EAS chemistry, but also that RegioSQM is applicable to essentially any molecule of interest. Consequently, chemists can apply the method in the planning of synthesis routes and confidently begin to contemplate novel approaches to target molecules where the strategic halogen atom be introduced at any stage of the synthesis pathway either to introduce a halogen in the final molecule, as protective groups to block more reactive sites to ensure the desired regiochemical control, or to introduce a halogen handle for a subsequent step. In other words, chemists would be in the position to predict the site selectivity of EAS reactions and focus on the synthesis of key scaffolds suitable for late-stage functionalization, for example to support efficient SAR explorations. Readers are encouraged to test RegioSQM on the webserver http://www.regiosqm.org.

## Conflicts of interest

There are no conflicts to declare.

## Supplementary Material

Supplementary information
