# Supplementary material for: Fast and accurate prediction of the regioselectivity of electrophilic aromatic substitution reactions
Source: Chem Sci. 2017 Nov 13;9(3):660–5. doi: 10.1039/c7sc04156j (PMC5869546; doi:10.1039/c7sc04156j)
Supplement: Supplementary file 1 [file SC-009-C7SC04156J-s001.pdf]

Electronic Supplementary Material (ESI) for Chemical Science.
This journal is © The Royal Society of Chemistry 2017

Section S1: Supporting links and supplementary data

Supporting links and supplementary data

RegioSQM is distributed on GitHub under the MIT license
https://github.com/jensengroup/RegioSQM/releases/tag/v1.0.

The SMILES strings, references, experimental EAS sites, etc can be found at
github.com/jensengroup/db-regioselectivity.

The study Kruszyk et al. contained 130 unique reactions. Several reactions had no yields reported and after removing these there are 118 left. These were used to test the performance of several methods.

As described in the main text PM3/chloroform or PM3/DMF have the highest success rate (Table S1 and S2) and that proton affinity is a better probe for reactivity than Br cation affinity at least using semiempirical methods (Table 3).

The high failure rate for PM6 Br cation calculations appears to be due to a strong large attraction between Br^+ and sp^2 nitrogen atoms, which leads to unphysically short Br^+-N distances and small Br^+-X-N angles compared to PM3.

One possible reason that newer methods such as PM6 perform worse is that the radii used in the COSMO calculations were optimized for AM1 and PM3, which means that PM6/COSMO calculations are less accurate (Table S4).

Table 1: Number of incorrect predictions of regioselectivity by the PM3 method in the gas phase, chloroform and DMF using a 1 kcal/mol cutoff. A maximum of 20 conformations per isomer is used.

	gas	chloroform	DMF
incorrect	11	2	2

Table 2: Number of incorrect predictions of regioselectivity by the PM3 method in the gas phase, chloroform and DMF using a 1 kcal/mol cutoff. A maximum of 20 conformations per isomer is used.

	PM3	AM1	PM6	PM6-DH+	PM7
incorrect	2	8	6	6	7

Table 3: Number of incorrect predictions using a maximum of 20 ("20 H") and 50 ("50 H") conformers and proton and Bromide cation (20 Br) affinity as a measure of reactivity.

	PM3	PM6
20 H	2	6
50 H	2	
20 Br	4	46

Table 4: Mean absolute error (MAE) and mean error (ME) of aqueous solvation energies computed using the COSMO method and the MNSOLV data set.

	AM1	PM3	PM6
MAE	3.6	3.4	3.9
ME	0	0.5	1.8

Section S2: Table listing numbering used in the paper and SI.

Table 5: This table lists numbering used in the paper and SI.

Paper	SI	Reference
1	1	[312]
2	15	[168]
3	170	[64]
4	199	[128]
5	514	[318]
6	75	[105]
7	373	[420]
8	367	[261]
9	163	[227, 366]
10	547	[180]
11	125	[59]
12	137	[269]
13	13	[394]
14	419	[283]

Section S3: Figure S1 Substituents of the compounds

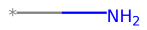

subst1

subst2

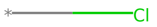

subst3

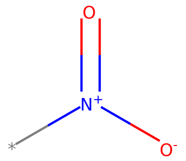

subst4

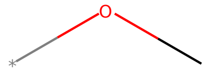

subst5

subst6

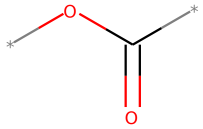

subst7

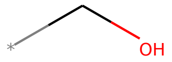

subst8

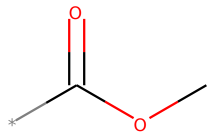

subst9

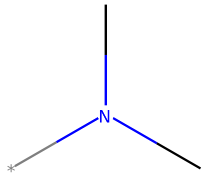

subst10

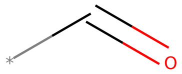

subst11

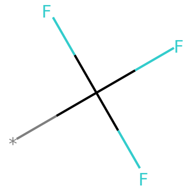

subst12

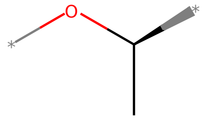

subst13

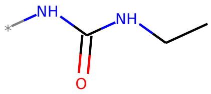

subst14

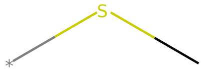

subst15

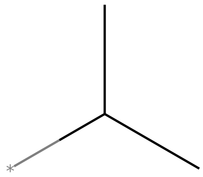

subst16

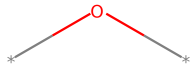

subst17

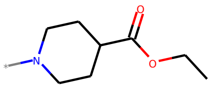

subst18

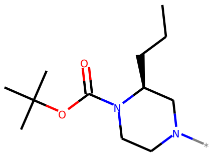

subst19

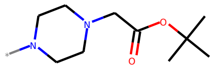

subst20

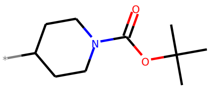

subst21

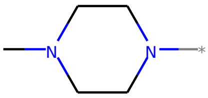

subst22

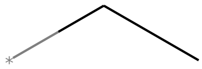

subst23

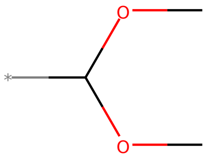

subst24

subst25

subst26

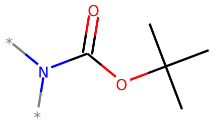

subst27

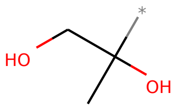

subst28

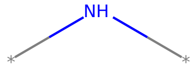

subst29

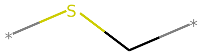

subst30

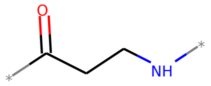

subst31

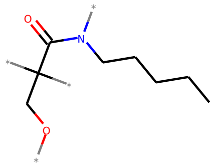

subst32

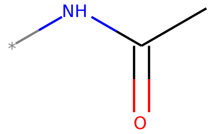

subst33

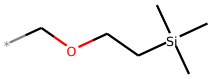

subst34

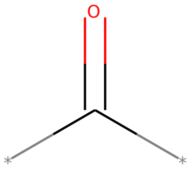

subst35

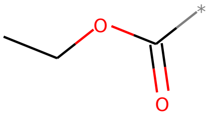

subst36

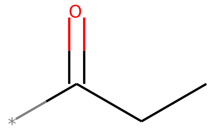

subst37

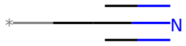

subst38

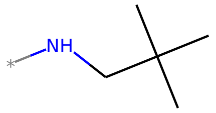

subst39

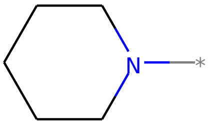

subst40

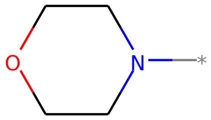

subst41

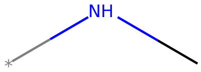

subst42

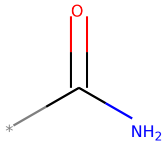

subst43

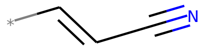

subst44

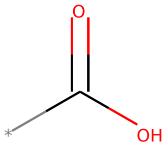

subst45

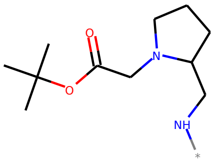

subst46

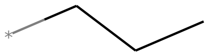

subst47

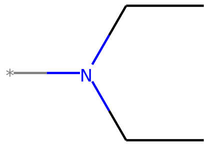

subst48

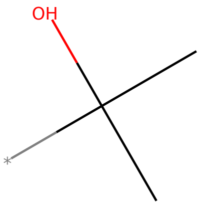

subst49

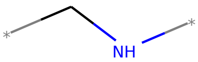

subst50

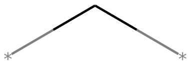

subst51

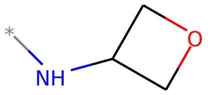

subst52

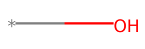

subst53

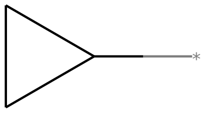

subst54

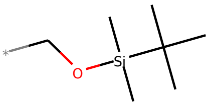

subst55

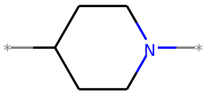

subst56

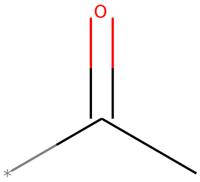

subst57

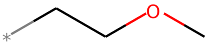

subst58

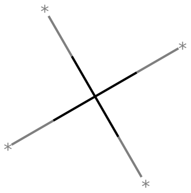

subst59

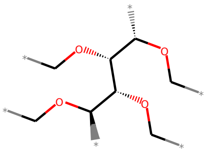

subst60

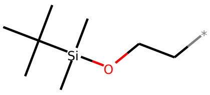

subst61

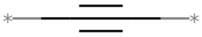

subst62

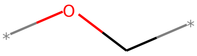

subst63

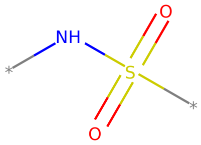

subst64

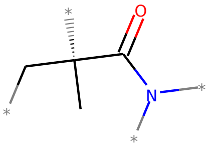

subst65

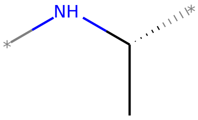

subst66

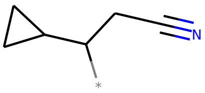

subst67

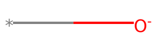

subst68

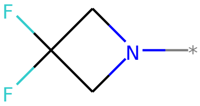

subst69

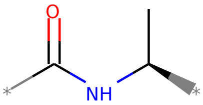

subst70

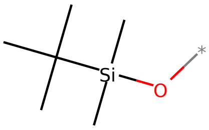

subst71

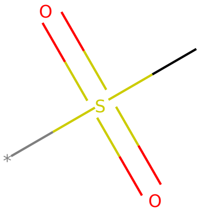

subst72

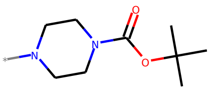

subst73

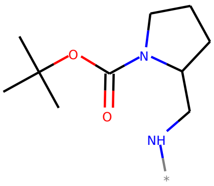

subst74

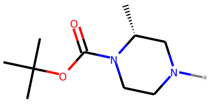

subst75

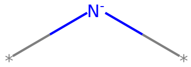

subst76

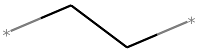

subst77

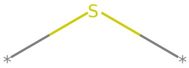

subst78

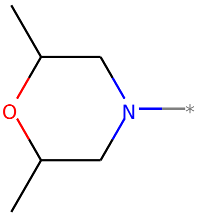

subst79

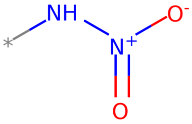

subst80

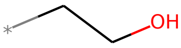

subst81

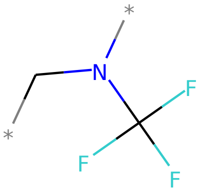

subst82

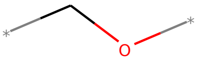

subst83

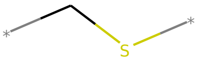

subst84

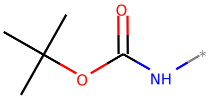

subst85

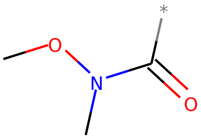

subst86

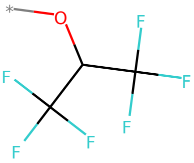

subst87

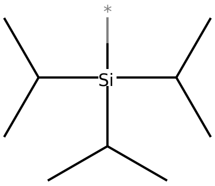

subst88

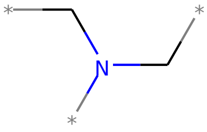

subst89

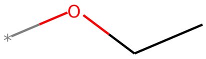

subst90

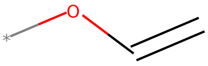

subst91

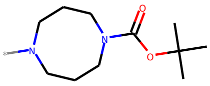

subst92

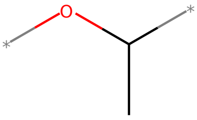

subst93

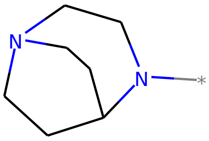

subst94

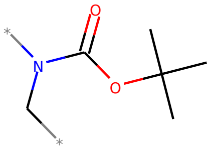

subst95

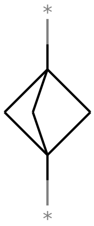

subst96

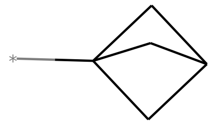

subst97

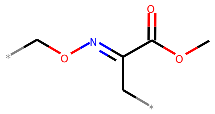

subst98

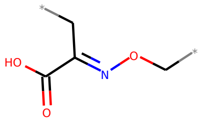

subst99

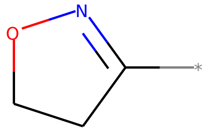

subst100

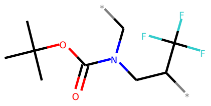

subst101

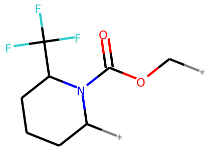

subst102

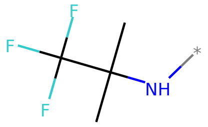

subst103

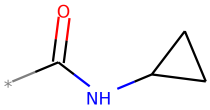

subst104

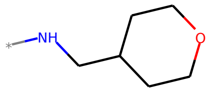

subst105

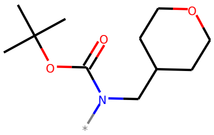

subst106

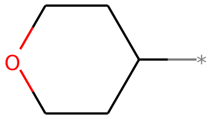

subst107

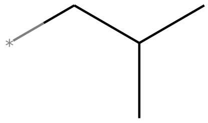

subst108

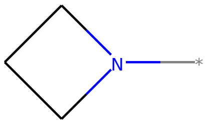

subst109

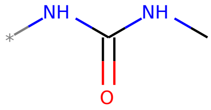

subst110

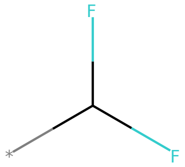

subst111

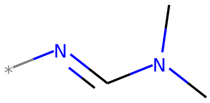

subst112

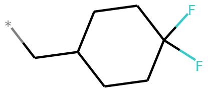

subst113

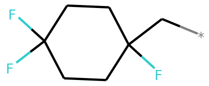

subst114

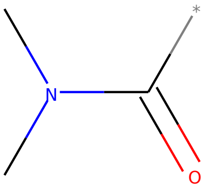

subst115

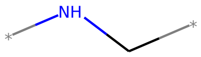

subst116

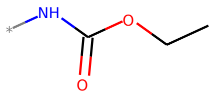

subst117

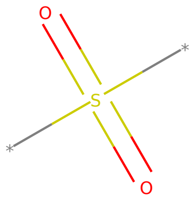

subst118

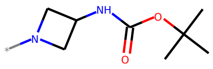

subst119

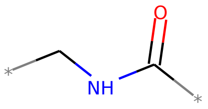

subst120

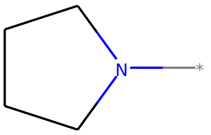

subst121

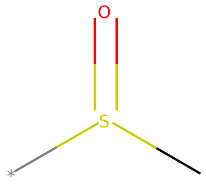

subst122

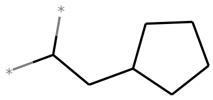

subst123

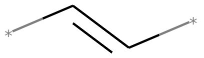

subst124

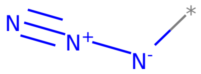

subst125

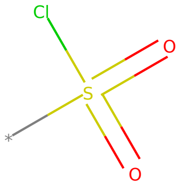

subst126

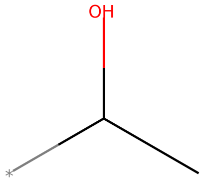

subst127

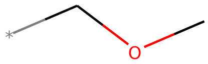

subst128

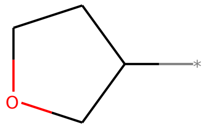

subst129

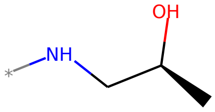

subst130

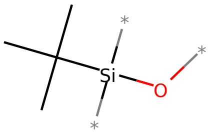

subst131

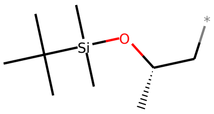

subst132

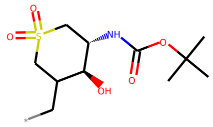

subst133

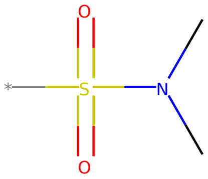

subst134

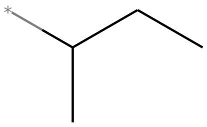

subst135

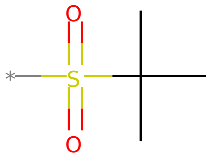

subst136

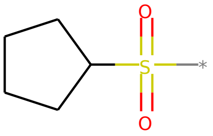

subst137

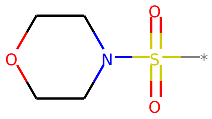

subst138

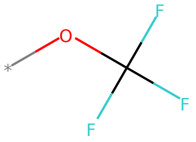

subst139

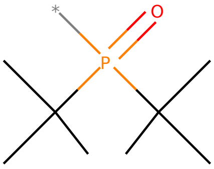

subst140

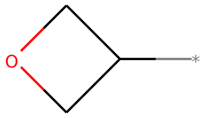

subst141

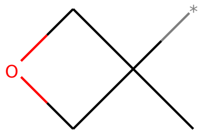

subst142

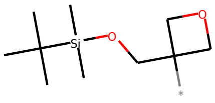

subst143

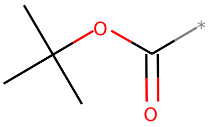

subst144

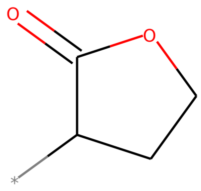

subst145

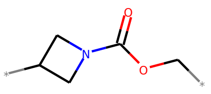

subst146

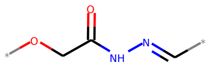

subst147

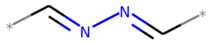

subst148

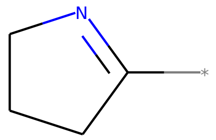

subst149

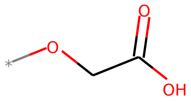

subst150

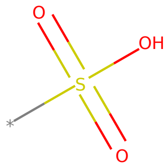

subst151

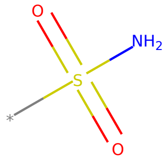

subst152

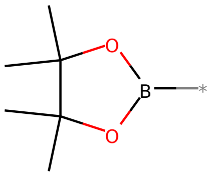

subst153

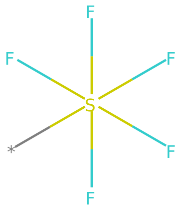

subst154

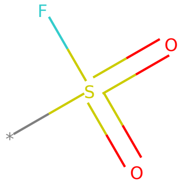

subst155

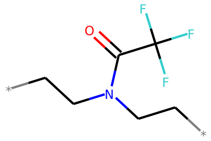

subst156

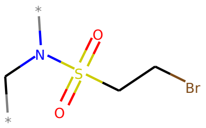

subst157

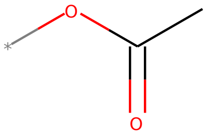

subst158

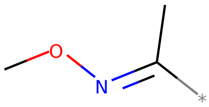

subst159

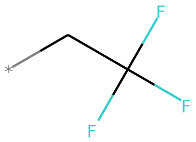

subst160

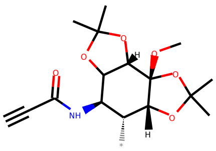

subst161

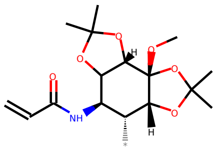

subst162

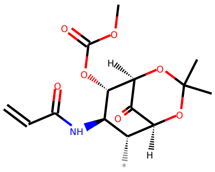

subst163

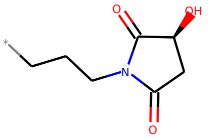

subst164

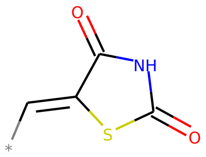

subst165

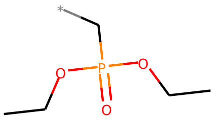

subst166

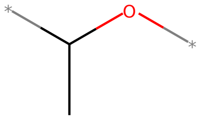

subst167

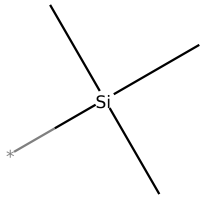

subst168

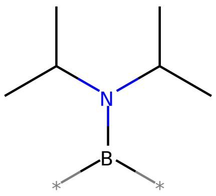

subst169

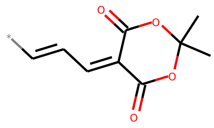

subst170

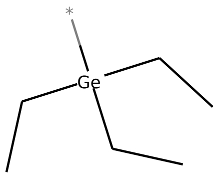

subst171

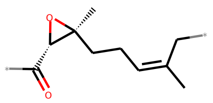

subst172

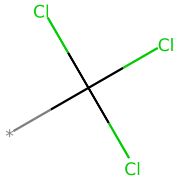

subst173

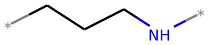

subst174

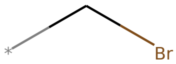

subst175

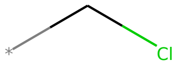

subst176

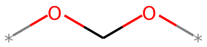

subst177

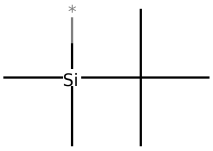

subst178

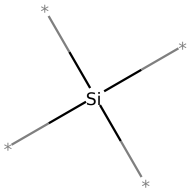

subst179

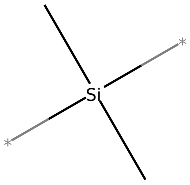

subst180

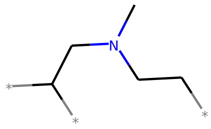

subst181

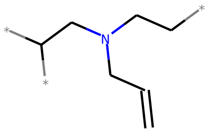

subst182

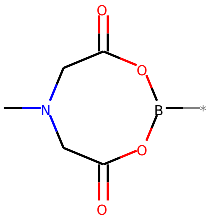

subst183

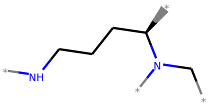

subst184

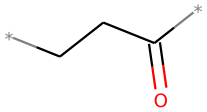

subst185

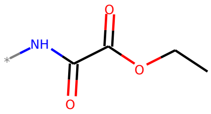

subst186

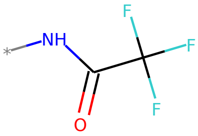

subst187

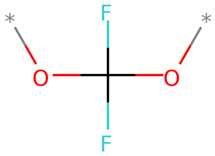

subst188

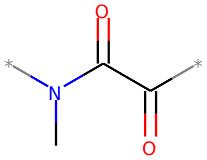

subst189

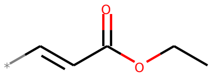

subst190

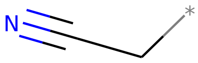

subst191

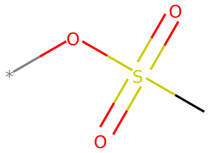

subst192

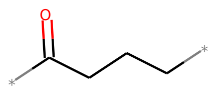

subst193

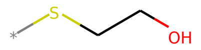

subst194

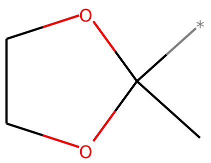

subst195

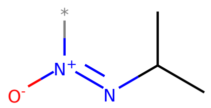

subst196

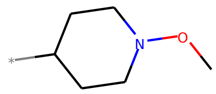

subst197

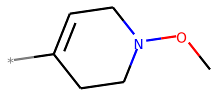

subst198

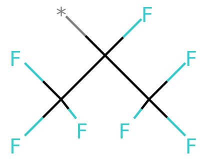

subst199

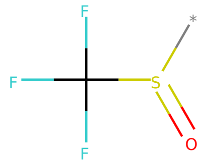

subst200

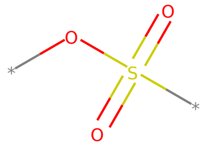

subst201

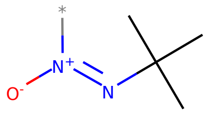

subst202

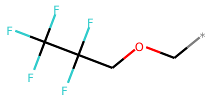

subst203

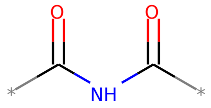

subst204

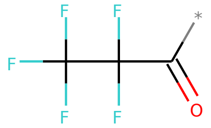

subst205

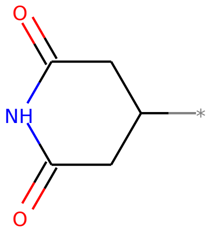

subst206

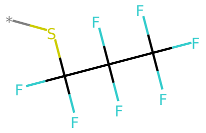

subst207

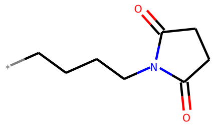

subst208

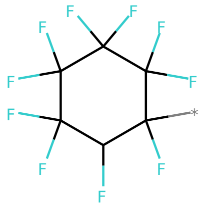

subst209

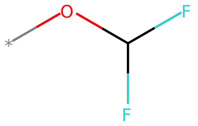

subst210

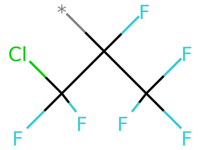

subst211

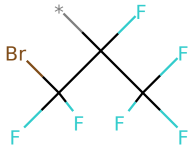

subst212

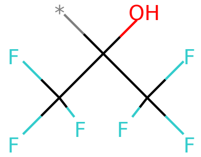

subst213

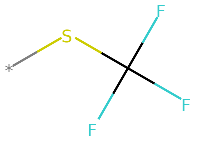

subst214

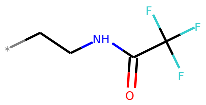

subst215

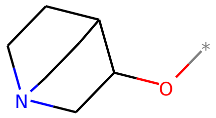

subst216

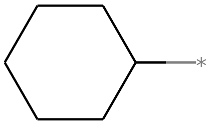

subst217

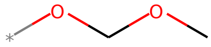

subst218

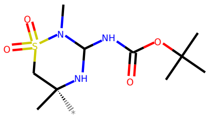

subst219

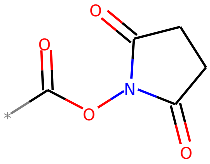

subst220

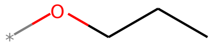

subst221

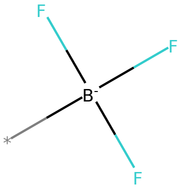

subst222

Section S4: Figure S2 Compounds sorted into EAS groups

Contents

1 Pyrroles 4

2 Furans 5

3 Thiophenes 7

4 Pyrazoles 8

5 Imidazoles 10

6 Isoxazoles 12

7 Oxazoles 13

8 Isothiazoles 14

9 Thiazoles 15

10 1,2,4-triazoles 16

11 Benzenes 17

12 Pyridines 22

13 2-Pyridones 26

14 Pyridazines 27

15 Pyrimidines 28

16 Pyrimidin-2(1H)-ones 30

17 Pyrimidin-4(3H)-ones 31

18 Pyrazines 32

19 Pyrazinones 34

20 4H-Furo[3,2-b]pyrroles 35

21 4H-Thieno[3,2-b]pyrroles 36

22 Imidazo[2,1-b][1,3,4]thiadiazoles 37

23 Indoles 38

24 Benzofurans 39

25 Benzo[b]thiophenes 40

26 Indazoles 41

27 1H-benzo[d]imidazoles 43

28 7-Azaindoles 44

29 6-azaindoles 45

30 5-azaindoles 46

31 4-azaindoles 47

32 Imidazo[1,2-a]pyridines 48

33 Furo[2,3-b]pyridines 49

34 Furo[2,3-c]pyridines 50

35 Furo[3,2-c]pyridines 51

36 furo[3,2-b]pyridines 52

37 [1,2,4]Triazolo[4,3-a]pyridines 53

38 1H-Pyrazolo[3,4-b]pyridines 54

39 1H-Imidazo[4,5-c]pyridines 55

40 Imidazo[1,2-a]pyrazines 56

41 Imidazo[1,2-c]pyrimidines 57

42 Imidazo[1,2-b]pyridazines 58

43 7H-Pyrrolo[2,3-d]pyrimidines 59

44 Pyrazolo[1,5-c]pyrimidines 60

45 Imidazo[1,5-a]pyrazines 61

46 Pyrrolo[2,1-f][1,2,4]triazines 62

47 Oxazolo[4,5-b]pyridines 63

48 Furo[2,3-d]pyrimidines 64

49 Furo[2,3-b]pyrazines 65

50 [1,2,4]Triazolo[4,3-b]pyridazines 66

51 Pyrazolo[1,5-a][1,3,5]triazines 67

52 Naphthalenes 68

53 Quinolines 69

54 Isoquinolines 70

55 Quinoxalines 71

56 1,5-Naphthyridines 72

57 Pyrido[4,3-d]pyrimidines 73

58 Pyrido[3,4-b]pyrazines 74

59 Furo[3,2-c]pyridin-4(5H)-ones 75

60 Furo[2,3-d]pyrimidin-4(3H)-ones 76

61 Imidazo[1,2-a]pyrazin-8(7H)-ones 77

62 Thiazolo[5,4-b]pyridin-5(4H)-ones 78

63 3,4-Dihydro-5H-[1,2,3]triazolo[4,5-b]pyridin-5-ones 79

64 1,3-Dihydro-2H-benzo[d]imidazol-2-ones **80**

65 Oxazolo[4,5-b]pyridin-2(3H)-ones **81**

66 Quinazolin-4(3H)-ones **82**

67 1,7-Naphthyridin-8(7H)-ones **83**

68 Pyrido[2,3-d]pyrimidin-7(8H)-ones **84**

69 2H-Chromen-2-ones **85**

1 Pyrroles

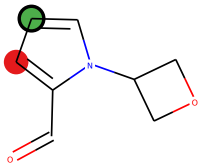

402 [111]

58% (NBS)

2 Furans

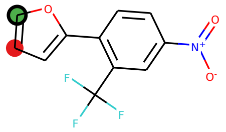

434 [457]
85% (NBS)

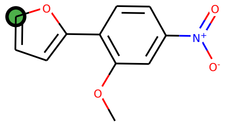

435 [457]
85% (NBS)

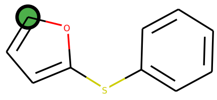

437 [264]
75% (NBS)

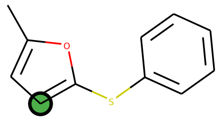

438 [436]
43% (NBS)

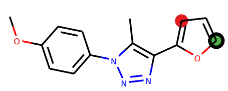

441 [343]
45% (NCS)

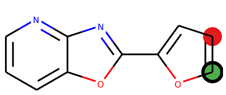

442 [170]
66% (Br_2)

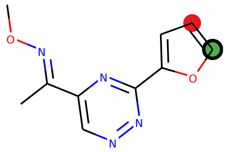

444 [84]
45% (NBS)

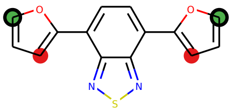

446 [444]
2xBr 65%, NBSD

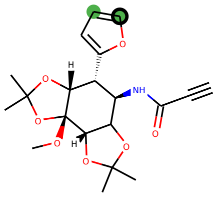

449 [320]
77% (NCS)

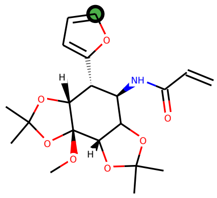

450 [320]
84% (NCS)

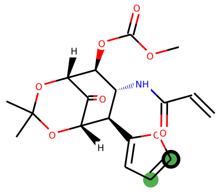

451 [321]
95% (NCS)

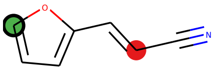

452 [376]
72% (NBS)

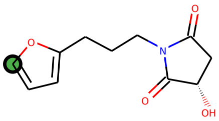

453 [376]
71% (NBS)

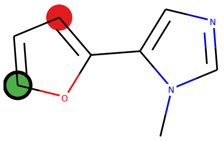

454 [1]
68% (Br_2)

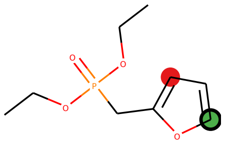

465 [344]
69% (Br_2)

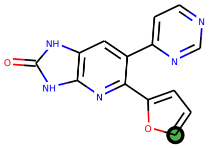

466 [383]
92% (Br_2)

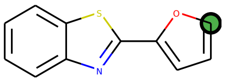

469 [300]
86% (Br_2)

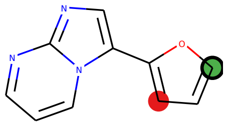

474 [365]
50% (Br_2)

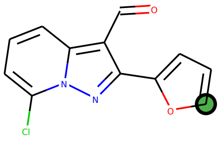

476 [190]
80% (NBS)

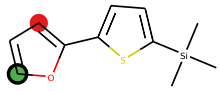

477 [308]
92% (NBS)

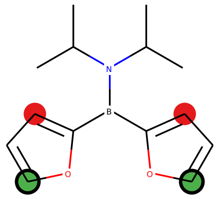

478 [237]

2xBr 70% (NBS)

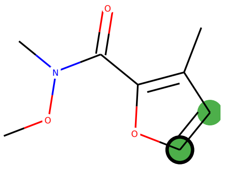

479 [401]

58% (Br_2)

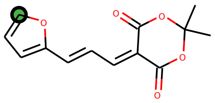

483 [265]

86% (NBS)

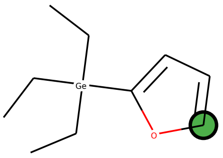

484 [289]

43% (NBS)

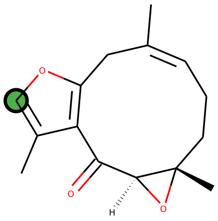

485 [379]

50% (NBS)

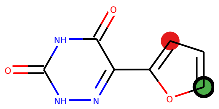

486 [3]

12, 76%

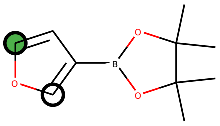

443 [241]

48% (NCS)

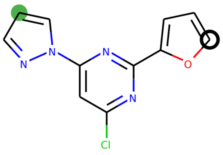

475 [137]

99% (NCS)

3 Thiophenes

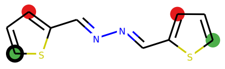

413 [303]
41% (NBS)

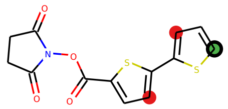

586 [47]
73% (NBS)

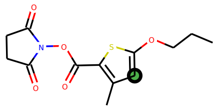

588 [182]
91% (Br_2)

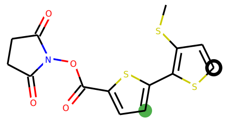

587 [447]
64% (NBS)

4 Pyrazoles

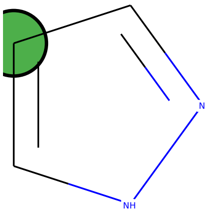

1 [313]
100% (NBS)

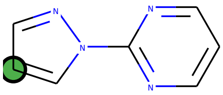

2 [393]
85% (Br_2)

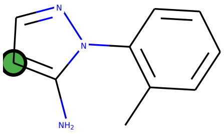

3 [49]
82% (Br_2)

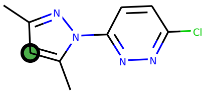

4 [397]
74% (SO_2Cl_2)

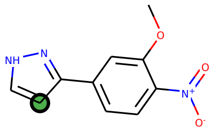

5 [12]
89% (NBS)

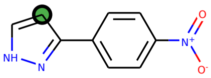

50 [13]
90% (NBS)

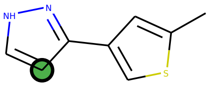

51 [388]
55% (NBS)

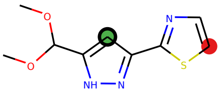

52 [219]
93% (Br_2, AcOH)

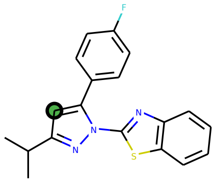

53 [345]
91% (NBS)

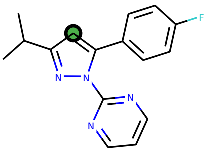

54 [345]
83% (NBS)

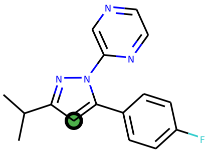

55 [345]
88% (NBS)

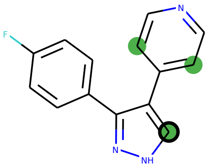

56 [255]
72% (NBS)

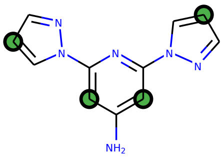

216 [103]
99% (I_2, 4xI)

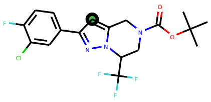

278 [407]
83% (NIS)

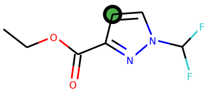

392 [85]
56% (NCS), 84% (NCS)

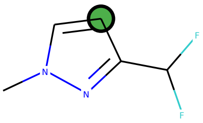

397 [459]
88% (NCS), 95% (NBS)

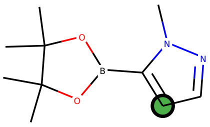

421 [127]
95% (NCS)

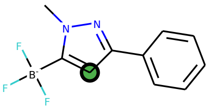

599 [178]
75% (NCS), 83% (NBS), 50% (NIS)

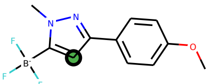

600 [178]
81% (NCS)

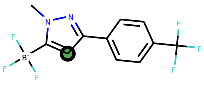

601 [178]
96% (NCS)

5 Imidazoles

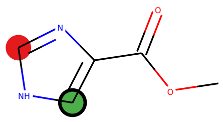

14 [120]

70% (NBS)

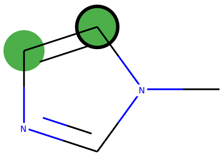

15 [168]

42% (NBS)

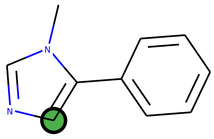

16 [61]

71% (NBS)

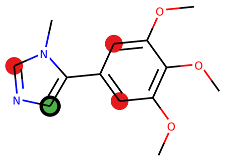

17 [59]

72% (NBS)

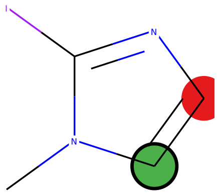

58 [76]

87% (NBS)

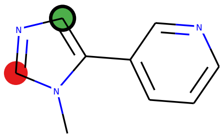

125 [59]

71% (NBS)

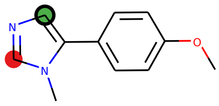

126 [58]

55% (NBS)

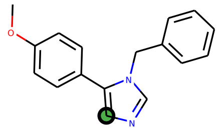

127 [60]

80% (NBS), 84% (NIS, TFA)

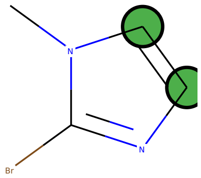

155 [167]

69% (I_2)

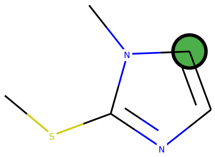

156 [121]

58% (NBS)

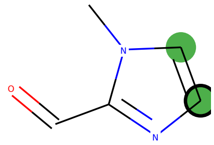

157 [382]

50% (HNO_3), 40% (NBS)

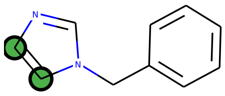

158 [16]

30% & 30% (NBS)

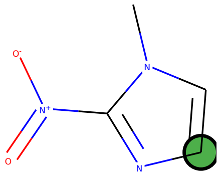

160 [336]

85% (NBS)

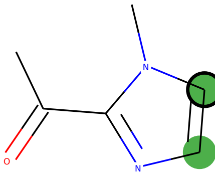

161 [380]

64% (NBS)

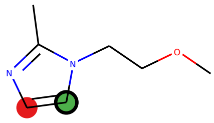

162 [208]

64% (NBS, K_2NO_3)

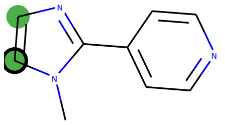

165 [251]

84% (NBS)

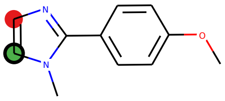

166 [306]

62% (Br_2)

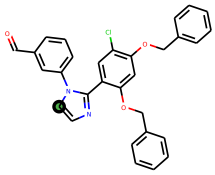

171 [254]

60% (NCS)

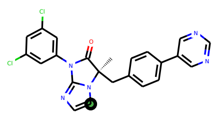

173 [425]

63% (NIS)

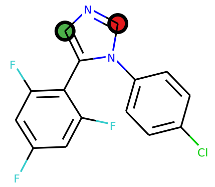

137 [270]

61% (Br_2), 68% (NCS)

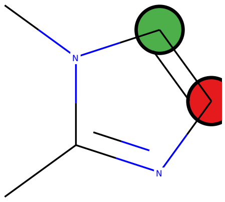

154 [62]

89% (NBS), b 50% (NBS)

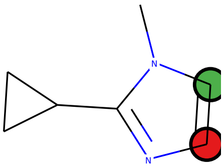

159 [181]

4-pos. 88% (NBS), 5-pos. 86% (NBS, K_2NO_3)

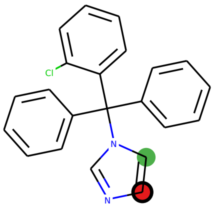

163 [228]

84% (NBS), 58% (NCS)

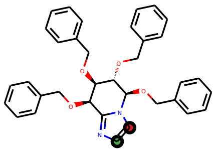

164 [402]

5-pos. 56%, 4+5-pos. 23% (NBS)

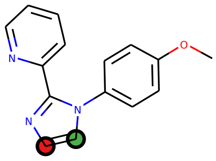

168 [342]

98% (Br_2)

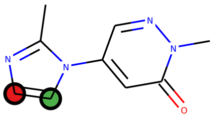

169 [222]

76% (NIS)

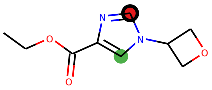

401 [71]

50% (NBS)

6 Isoxazoles

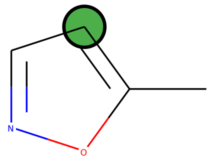

6 [455]
88% (NBS)

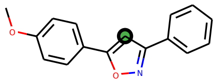

7 [387]
97% (NIS, TFA)

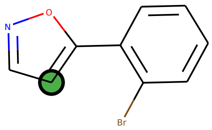

8 [276]
84% (NBS)

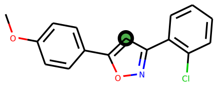

9 [150]
73% (NBS)

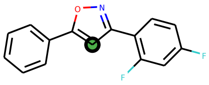

141 [377]
77% (NBS)

7 Oxazoles

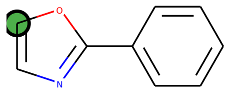

43 [89]
56% (Br_2)

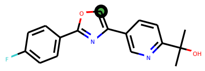

119 [114]
66% (NBS)

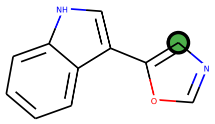

122 [450]
83% (NBS)

8 Isothiazoles

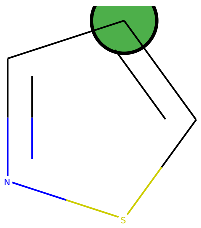

10 [351]
40% (Br_2)

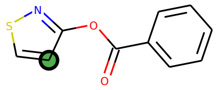

11 [235]
88% (NBS)

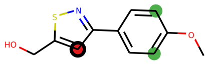

13 [396]
34% (NBS)

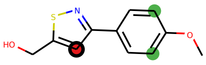

598 [?]
55% (NCS)

9 Thiazoles

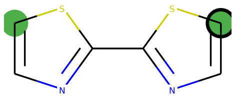

18 [395]
97% (NBS)

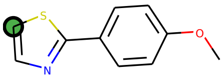

19 [333]
94% (NBS)

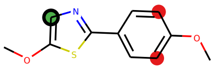

20 [398]
80% (NBS)

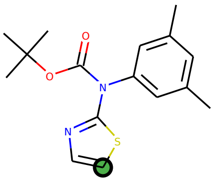

59 [372]
90% (NBS)

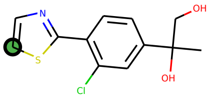

60 [399]
100% (NBS)

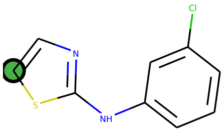

61 [177]
65% (NBS)

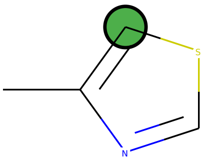

62 [110]
37% (Br_2, AcOH)

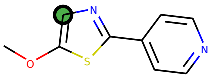

64 [454]
96% (NBS)

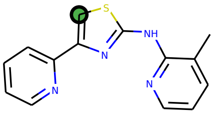

66 [211]
85% (NBS), 93% (NCS)

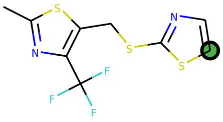

67 [149]
96% (NBS), 63% (NIS), 73% (HNO_3)

10 1,2,4-triazoles

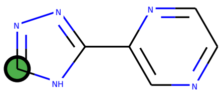

147 [346]
65% (Br_2/NaOH)

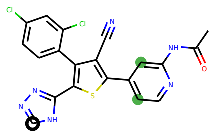

149 [46]
80% (NBS)

11 Benzenes

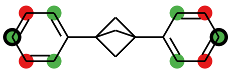

274 [146]
63% 2x para (I_2)

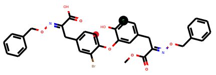

276 [134]
100% (NBS)

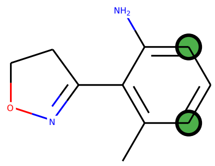

277 [285]
4-Br 75%, 6-Br 2%, 4+6-diBr 3%

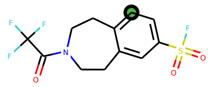

428 [189]
50% (NIS)

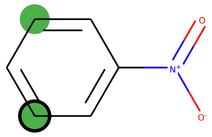

488 [281]
91% (NBS)

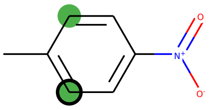

489 [421]
93% (NBS)

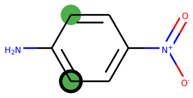

490 [141]
98% (NBS)

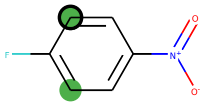

493 [6]
91% (NBS), 91% (NBS)

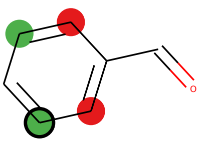

494 [159]
84% (NBS)

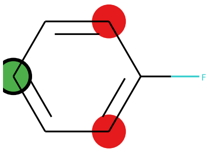

495 [349]
96% (NBS)

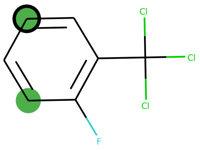

496 [400]
C12 96%

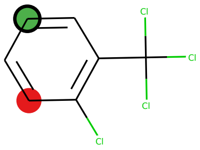

497 [400]
C12 82% 3-pos + 6-pos 8%

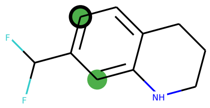

498 [359]
60% (NBS)

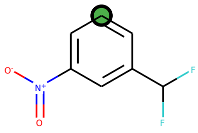

499 [25]
56% (NBS)

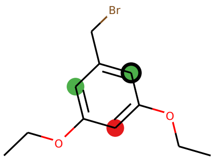

500 [442]
91% (NBS)

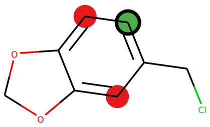

501 [249]
99% (NBS)

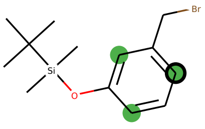

502 [277]
96% (NBS)

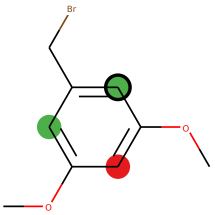

503 [117]
86% (NBS)

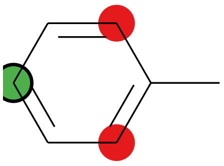

504 [247]
74% (Br_2)

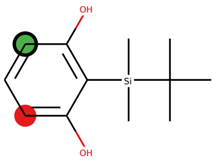

509 [215]
75% (NBS)

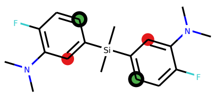

511 [95]
83% (NBS)

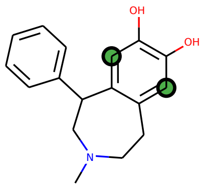

514 [318]
2xCl 55% (AcOH/SO_2Cl_2)

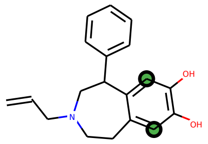

515 [318]
2xCl 55% (AcOH/SO_2Cl_2)

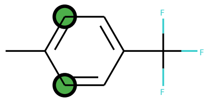

516 [126]
59% (NBS)

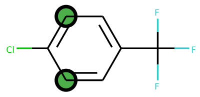

517 [159]
86% (NBS)

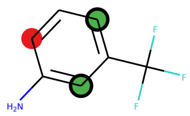

518 [169]
92% (NBS)

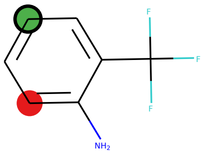

519 [112]
90% (NBS)

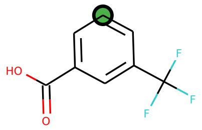

520 [288]
90% (NBS)

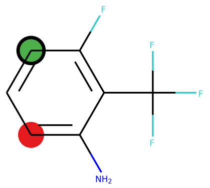

521 [96]
98% (NBS)

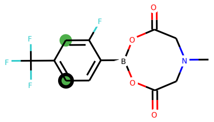

522 [124]
99% (NBS)

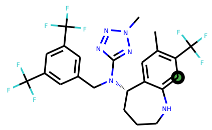

523 [109]
92% (NBS)

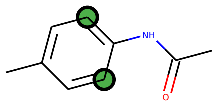

526 [5]
92% (NBS), 92% (NBS)

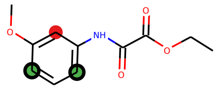

529 [65]
2xBr 98% (NBS)

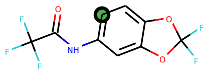

530 [97]
64% (NBS)

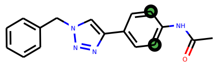

531 [185]
74% (NBS)

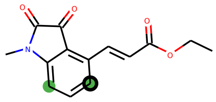

532 [391]
95% (NBS)

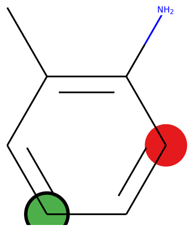

534 [327]
71% (NBS)

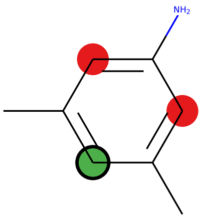

535 [460]
94% (NBS)

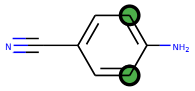

536 [390]
69% (NBS)

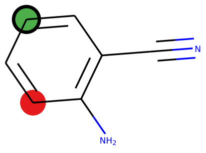

537 [238]
2xBr 77% (NBS)

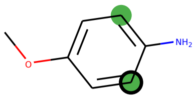

539 [290]
62% (NBS)

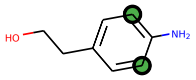

540 [363]
95% (NBS)

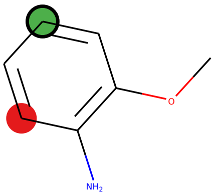

541 [422]
85% (NBS)

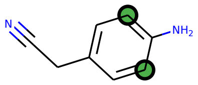

542 [176]
74% (NBS)

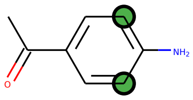

543 [315]
100% (NBS)

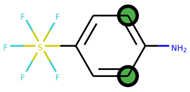

546 [180]
79% (NBS)

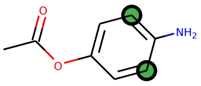

549 [30]
83% (NBS)

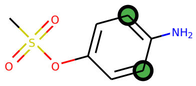

550 [322]
99% (NBS)

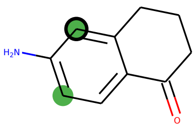

551 [358]
87% (NBS)

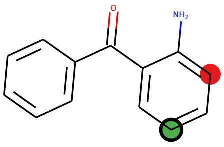

552 [287]
100% (NBS)

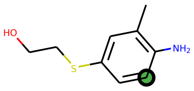

553 [4]
91% (NBS)

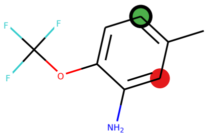

554 [451]
99% (NBS)

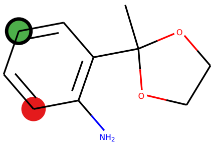

555 [259]
96% (NBS)

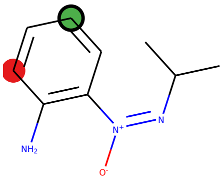

556 [282]
2xBr 92% (NBS)

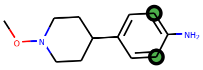

558 [43]
75% (NBS)

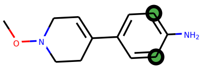

559 [216]
75% (NBS)

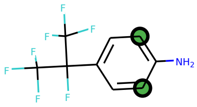

560 [260]
90% (NBS)

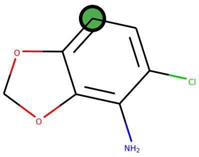

562 [386]
85% (NBS)

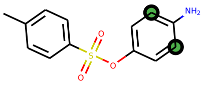

563 [123]
95% (NBS)

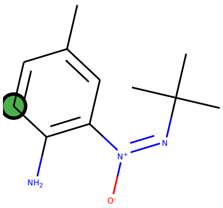

564 [282]
91% (NBS)

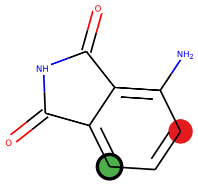

566 [27]

74% (NBS)

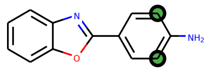

567 [307]

96% (NBS)

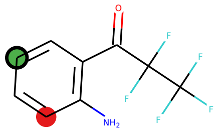

568 [452]

94% (NBS)

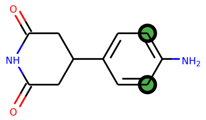

569 [217]

67% (NBS)

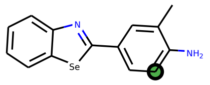

570 [8]

83% (NBS)

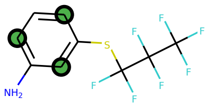

571 [325]

3xBr 89% (NBS)

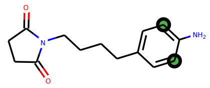

572 [256]

2xBr 90% (NBS), 1xBr 80% (NBS)

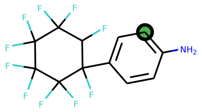

573 [304]

2xBr 96% (NBS)

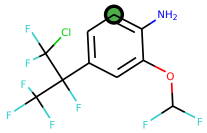

575 [385]

96% (NBS)

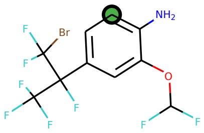

576 [304]

96% (NBS)

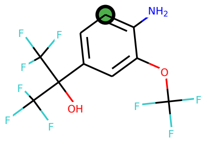

577 [324]

92% (NBS)

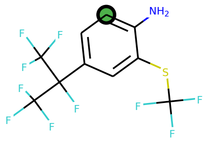

578 [326]

73% (NBS)

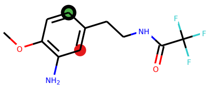

579 [252]

72% (NBS)

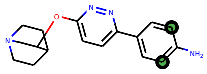

582 [226]

53% (NBS)

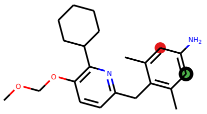

583 [244]

91% (NBS)

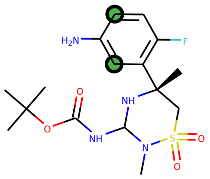

584 [373]

86% (NBS)

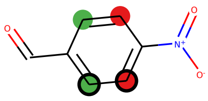

492 [352]

2xBr 96% (NBS)

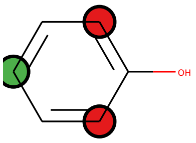

505 [341]

ortho + para 90% (Br_2), para 98% (NBS), 3xBr 97% (Br_2)

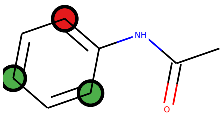

524 [5]

92% (NBS)

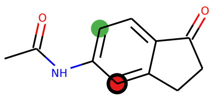

528 [428]

74% (NBS)

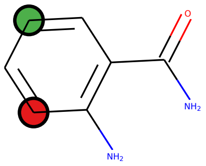

545 [55]

2xBr 91% (NBS), 2xBr 78% (NBS)

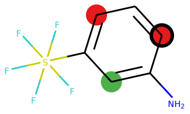

547 [180]

79% (NBS)

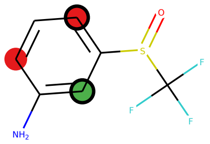

561 [66]

1xBr (1:1) 73% (NBS)

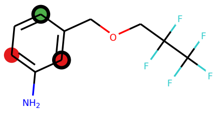

565 [239]

2xBr 77% (NBS)

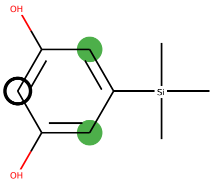

508 [278]

98% (NBS)

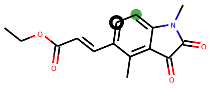

533 [391]

92% (NBS)

12 Pyridines

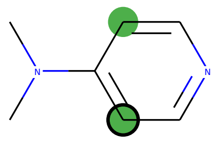

21 [340]
93% (Br_2, K_2CO_3)

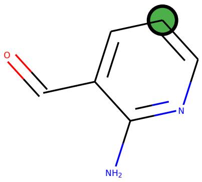

22 [158]
88% (Br_2)

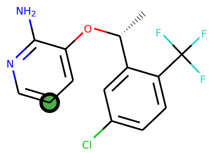

23 [424]
69% (NBS)

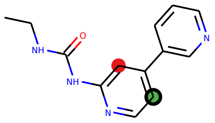

24 [54]
91% (NBS)

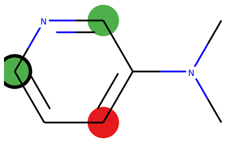

48 [347]
68% (NBS)

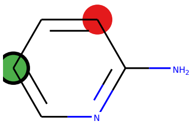

68 [271]
98% (NBS), 80% (NBS), 93% (I_2)

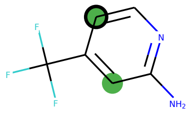

69 [93]
80% (NBS), 83% (NBS)

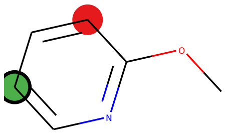

70 [295]
90% (Br_2, AcOH, NaOAc), 81% (5-Br; NBS), 73% (3,5-diBr; Br_2), 80% (NBS)

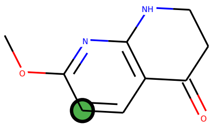

71 [100]
85% (NBS)

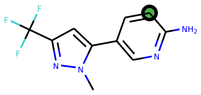

73 [173]
30% (NBS)

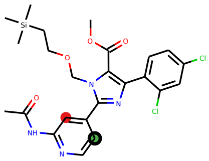

75 [106]
87% (NBS)

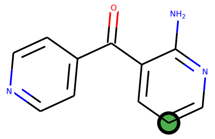

76 [195]
80% (NBS)

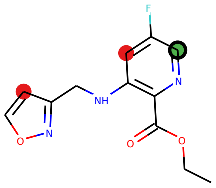

124 [72]
34% (NBS)

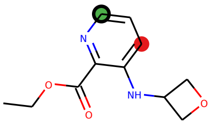

128 [72]
76% (NBS)

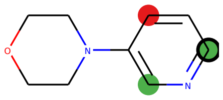

129 [207]
74% (NBS)

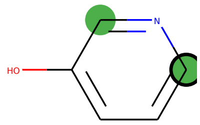

130 [164]
75% (NaI, NaOCl)

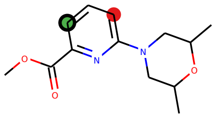

215 [430]
91% (NBS)

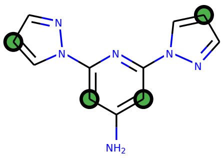

216 [103]
99% (I_2, 4xI)

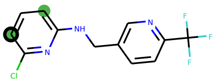

217 [448]
80% (NBS)

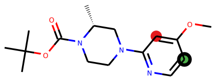

218 [136]
89% (NBS)

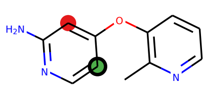

221 [17]
92% (Br_2)

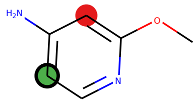

223 [311]
98% (NBS)

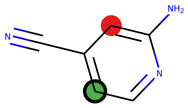

224 [39]
78% (NBS)

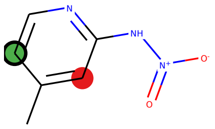

225 [148]
83% (Br_2))

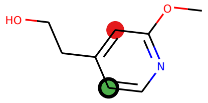

228 [426]
91% (Br_2)

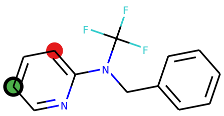

229 [242]
94% (Br_2)

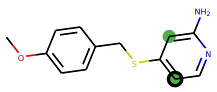

234 [19]
54% (Br_2)

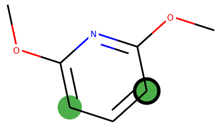

236 [98]
80% (NBS)

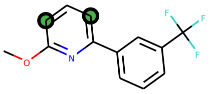

237 [240]
32% (3-Cl) + 50% (5-Cl) NCS

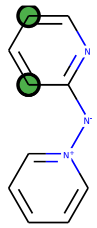

238 [94]
a 61% (2xI, NIS), 73% (2xBr, NBS), b 90% (NIS), 71% (NBS))

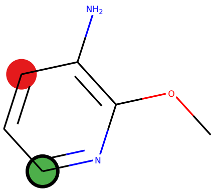

239 [243]
80% (NBS)

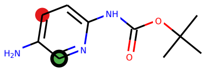

241 [101]
66% (NBS)

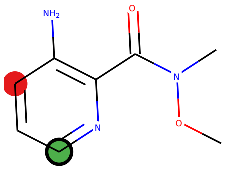

242 [138]
59% (Br_2)

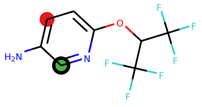

243 [323]
90% (NBS), 86% (NCS))

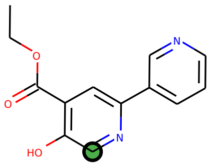

244 [63]
80% (NBS)

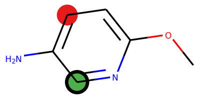

247 [81]
100% (Br_2)

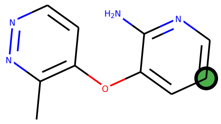

248 [18]
95% (NBS)

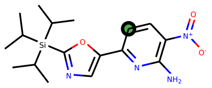

249 [414]
88% (NBS)

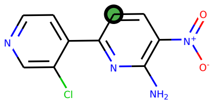

250 [414]
93% (NBS)

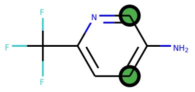

252 [233]
85% (NCS then NBS)

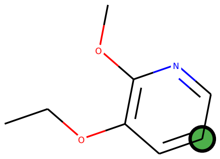

253 [67]

2-Br 56%, 3-Br (Br_2)

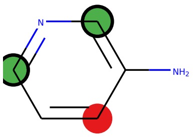

255 [98]

2-Br 40%, 6-Br 32%, 2+6-diBr 14% (NBS)

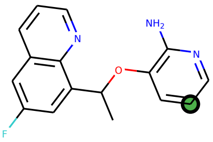

256 [91]

99% (NBS)

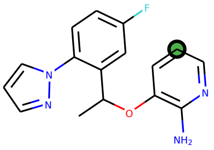

257 [91]

93% (NBS)

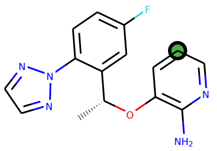

258 [91]

73% (NBS)

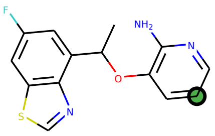

259 [91]

51% (NBS)

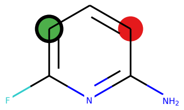

296 [51]

98% (NBS)

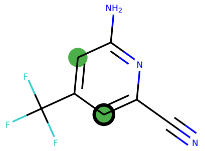

297 [431]

70% (NBS)

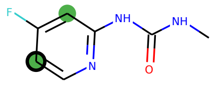

298 [157]

72% (NBS)

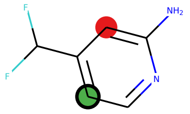

391 [125]

79% (NBS)

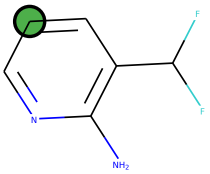

395 [174]

64% (NBS)

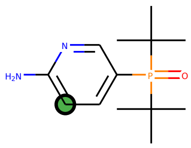

400 [4]

68% (NBS)

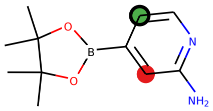

420 [283]

85% (NBS)

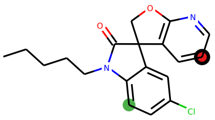

74 [80]

76% (NBS)

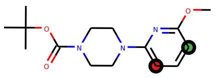

219 [136]

58% (a-to-OMe), 13% (a-to-N) NBS

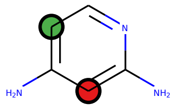

220 [319]

90% (a-to-2xN), 9% regioisomer, I_2

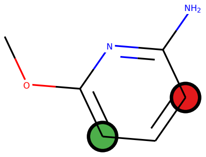

235 [52]

73% (3-pos), 11% (5-pos), NCS

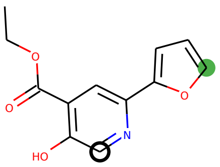

245 [63]

91% (NBS)

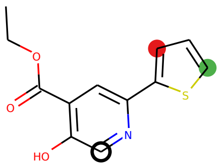

246 [63]

71% (NBS)

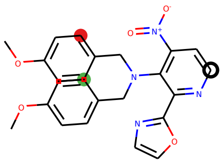

251 [415]

52% (NBS)

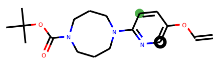

254 [38]

52% (NBS)

13 2-Pyridones

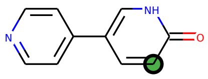

77 [378]
90% (NBS)

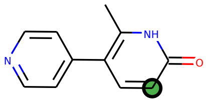

78 [236]
82% (NBS), 93% (NIS)

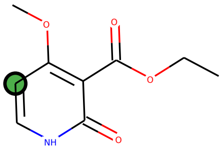

79 [225]
90% (NBS, NH_4NO_3)

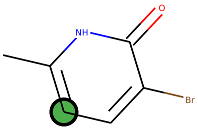

80 [368]
91% (NIS)

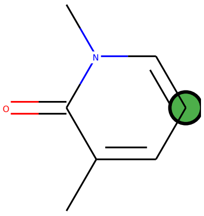

81 [187]
96% (NBS)

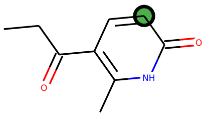

82 [381]
82% (NBS)

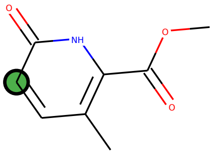

83 [155]
70% (NBS)

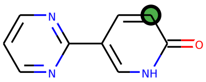

84 [330]
51% (NBS)

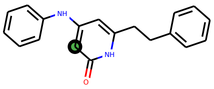

213 [99]
92%(NIS)

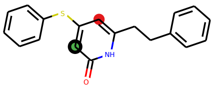

214 [99]
75% (NBS), 91% (NIS)

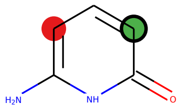

227 [317]
65% (NBS)

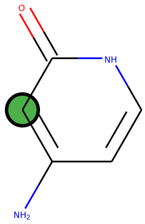

230 [299]
70% (NBS)

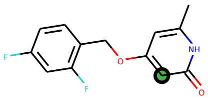

233 [26]
85% (NCS)

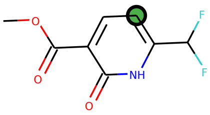

398 [458]
65% (NBS)

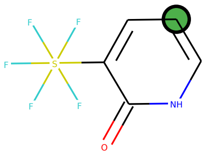

427 [230]
74% (NBS)

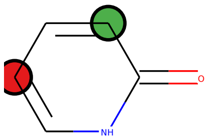

226 [369]
95% (NBS), 34% (2xBr) + 29% (4-Br) + 3% (3-Br) NBS, 76% (5-Br) + 11% (3-Br)

14 Pyridazines

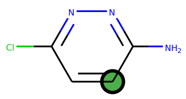

32 [272]
82% (Br_2, $NaHCO_3$)

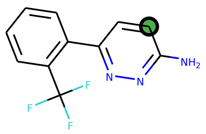

33 [102]
95% (Br_2, $NaHCO_3$)

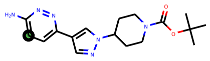

34 [7]
57% (Br_2, $NaHCO_3$)

15 Pyrimidines

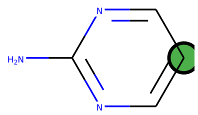

25 [198]
100% (NBS, NH_4OAC)

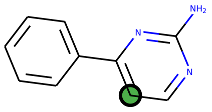

26 [56]
51% (NBS)

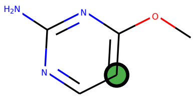

27 [93]
96% (NBS)

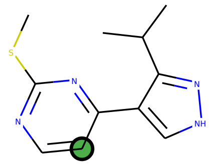

28 [113]
90% (NBS)

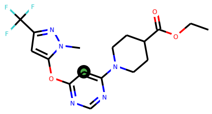

29 [234]
57% (NBS)

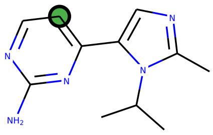

85 [231]
74% (NIS)

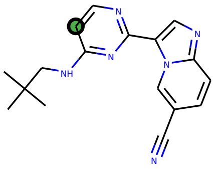

86 [162]
98% (NBS)

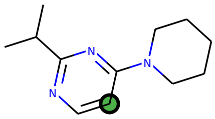

87 [87]
82% (NBS)

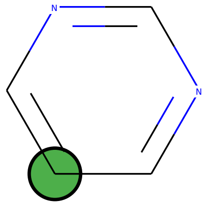

88 [266]
88% (Br_2)

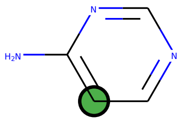

89 [130]
59% (NBS)

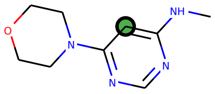

90 [246]
78% (Br_2)

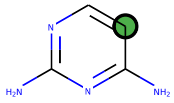

91 [93]
74% (NBS), 87% (Br_2)

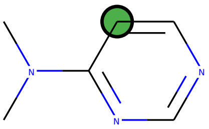

92 [340]
91% (NBS)

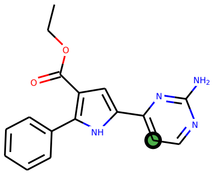

93 [406]
58% (NCS), 65% (NBS)

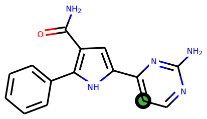

94 [406]
72% (NCS), 75% (NBS)

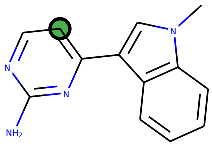

96 [360]
94% (NBS)

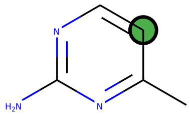

97 [93]
99% (NBS)

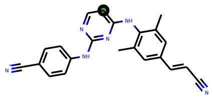

98 [191]
86% (NBS)

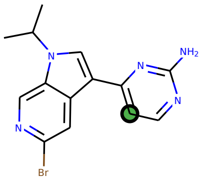

324 [213]
69% (NIS), 79% (NCS)

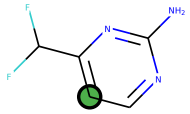

390 [125]
98% (NBS)

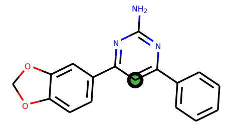

593 [405]

70% (Br_2)

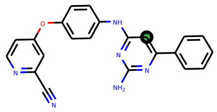

597 [153]

83% (Br_2)

16 Pyrimidin-2(1H)-ones

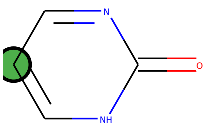

99 [416]

50% (Br_2), 84% (Br_2)

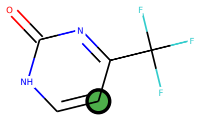

100 [331]

80% (Br_2, KOAc)

17 Pyrimidin-4(3H)-ones

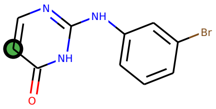

101 [293]
82% (NCS), 73% (NBS), 97% (NIS)

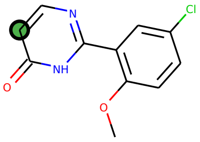

102 [355]
86% (Br_2)

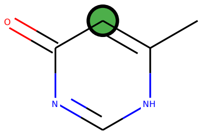

103 [143]
74% (NCS), 94% (NBS), 100% (ICl, AcOH)

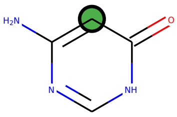

104 [356]
47% (Br_2)

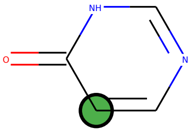

105 [70]
92% (Br_2)

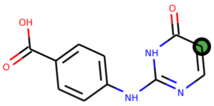

106 [446]
95% (Br_2, AcOH)

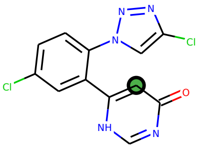

594 [133]
93% (NBS)

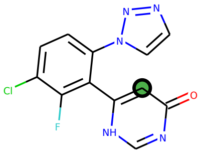

595 [132]
54% (NCS)

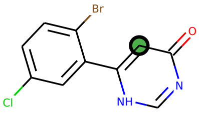

596 [133]
94% (NCS)

18 Pyrazines

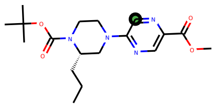

30 [253]

98% (NBS)

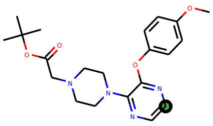

31 [129]

70% (NBS)

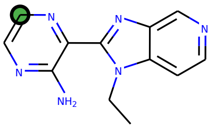

49 [21]

79% (NBS), 78% (NBS)

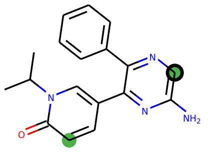

107 [439]

79% (NBS)

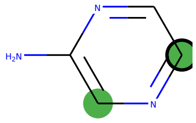

108 [275]

82% (NBS), 5-Br 62% + 3,5diBr 12% (NBS), 72% (NBS), 90% (NBS); 83% (NBS), 77% (NBS)

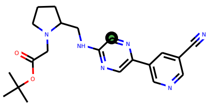

109 [129]

66% (NBS)

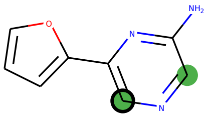

131 [410]

44% (NBS)

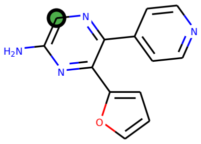

132 [411]

75% (NBS), 75% (NBS)

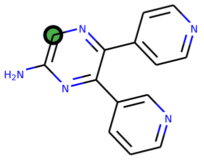

133 [412]

30% (NBS)

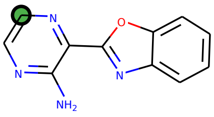

134 [50]

62% (NBS)

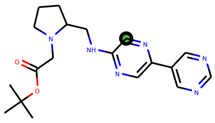

135 [129]

77% (NBS)

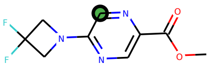

192 [68]

77% (NBS)

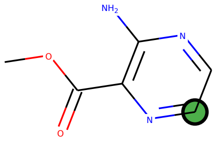

193 [267]

88% (NIS), 88% (NIS), 96% (NBS), 70% (Br_2), 72% (NCS)

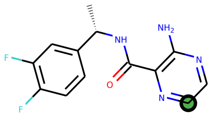

194 [33]

84% (NBS)

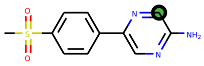

196 [104]

84% (NBS), 65% (NIS)

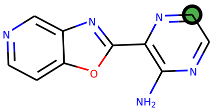

198 [15]

95% (NBS)

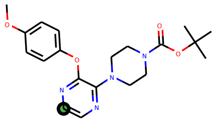

199 [128]

70% (NBS)

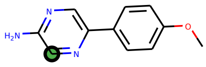

200 [184]

70% (Br_2)

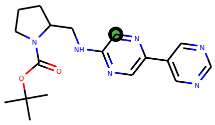

201 [128]

77% (NBS)

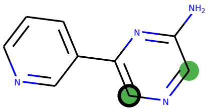

202 [161]

72% (NBS)

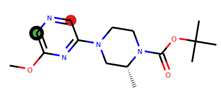

203 [136]
26%/26% (NBS) two mono-Br

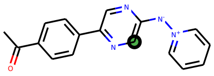

205 [10]
84% (NBS)

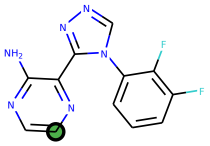

206 [34]
78% (NBS)

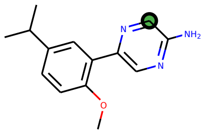

207 [20]
74% (NBS)

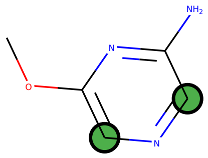

211 [52]
45% (3-Br), 16% (5-Br), 10% (3+5-Br_2)

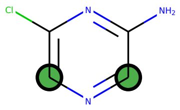

212 [166]
15% (3-Br), 43% (5-Br), 88% (NIS), 2xBr 95% (NBS)

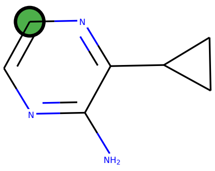

284 [188]
64% (NBS)

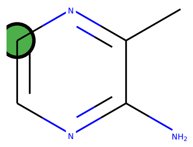

285 [429]
80% (NCS)

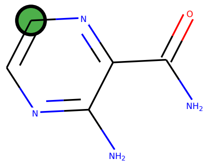

286 [438]
95% (Br_2)

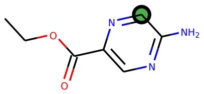

289 [210]
78% (NBS)

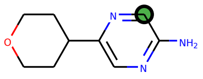

290 [40]
97% (NBS)

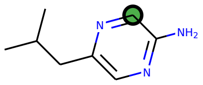

291 [314]
77% (Br_2)

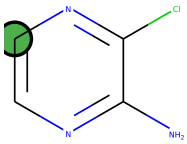

292 [186]
76% (NIS), 71% (NIS)

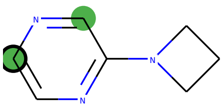

293 [232]
73% (NBS)

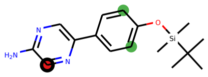

195 [218]
81% (NBS)

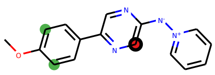

209 [10]
86% (NBS)

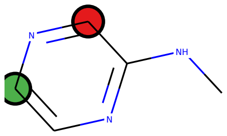

283 [165]
65% (NIS)

19 Pyrazinones

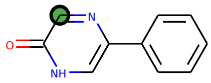

190 [370]

59% (Br_2), 76% also p-phenyl (Br_2)

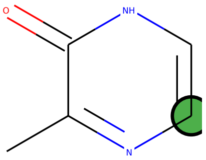

197 [108]

77% (NBS)

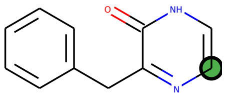

204 [23]

74% (NBS)

20 4H-Furo[3,2-b]pyrroles

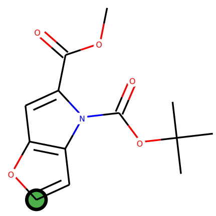

472 [205]

65% (NBS)

21 4H-Thieno[3,2-b]pyrroles

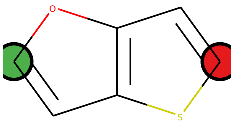

471 [206]

2xBr 92% (NBS), 67% (96:4)

mono-O over mono-S

22 Imidazo[2,1-b][1,3,4]thiadiazoles

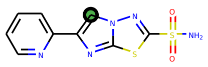

418 [224]
84% (Br_2)

23 Indoles

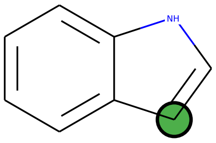

35 [364]

96% (Br_2)

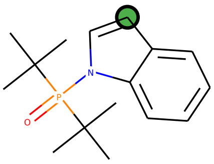

399 [437]

I2, 1xI, 3pos, 48%, NCS, 1xCl 3 pos, 81% + 2,3-diCl, 10%

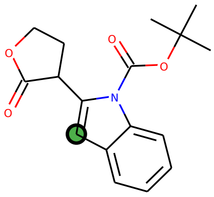

407 [78]

95% (NBS)

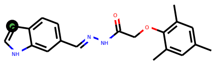

412 [115]

32% (NBS)

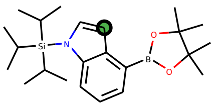

425 [245]

98% (NCS)

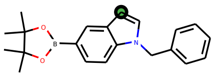

426 [11]

82% (NCS)

24 Benzofurans

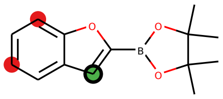

424 [241]
39% (NCS)

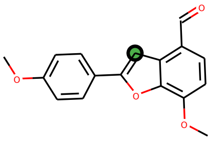

430 [417]
60% (NBS)

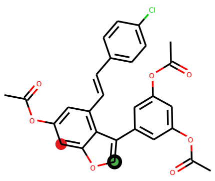

431 [417]
70% (NBS)

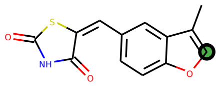

461 [361]
66% (Br_2)

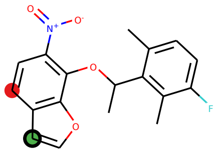

468 [274]
84% (Br_2)

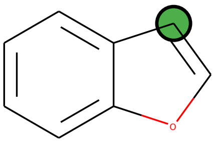

470 [212]
3xBr 76% (Br_2),2xBr (2+3 positions), 84% (Br_2)

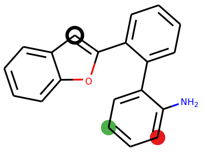

433 [196]
12, 94%

25 Benzo[b]thiophenes

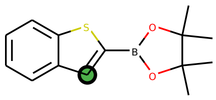

423 [241]
70% (NCS)

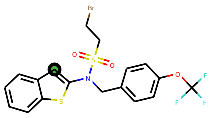

429 [291]
96% (NBS), 100% (NCS)

26 Indazoles

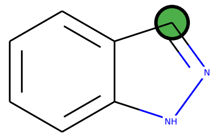

353 [294]
82% (NBS), 60% (NCS)

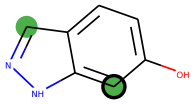

354 [44]
54% (NCS), 95% (HNO_3)

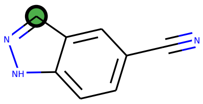

355 [443]
89% (NBS)

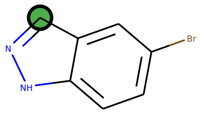

356 [367]
91% (NCS)

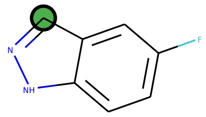

357 [192]
59% (NBS)

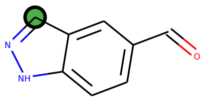

358 [302]
97% (NCS), 82% (NBS)

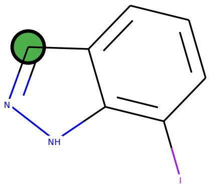

359 [310]
79% (NBS)

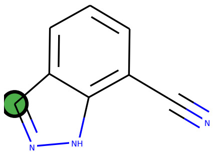

360 [310]
74% (NBS)

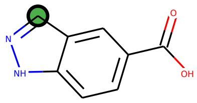

361 [131]
84% (NCS)

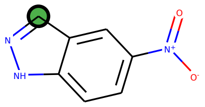

362 [353]
90% (NBS)

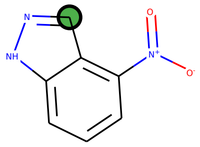

363 [9]
84% (NBS), 90% (NBS)

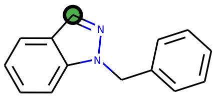

364 [440]
90% (NBS)

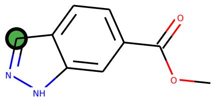

365 [413]
96% (NBS)

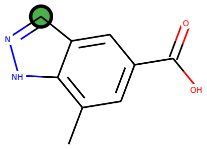

366 [131]
98% (NCS)

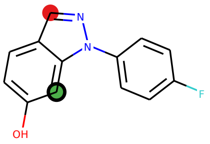

370 [179]
63% (NBS)

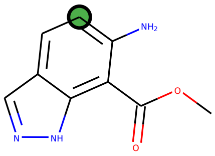

371 [286]
90% (NIS), 82% (NBS)

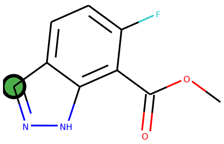

372 [83]
69% (NBS)

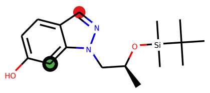

374 [298]
77% (NBS)

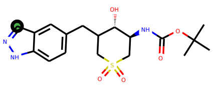

375 [362]
97% (NBS)

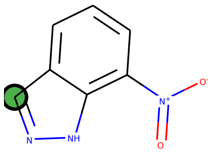

383 [353]
90% (NBS), 84% (NBS), 90% (NBS)

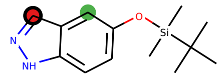

367 [261]

98% (NIS)

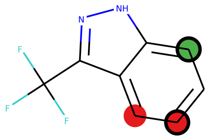

368 [456]

5-Br 35%, 7-Br 6%

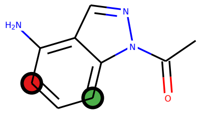

369 [90]

5-Br 66%, 7-Br 11%

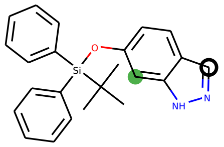

373 [420]

57% (NCS)

27 1H-benzo[d]imidazoles

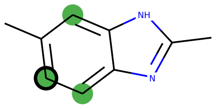

377 [250]
58% (NCS)

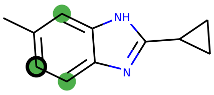

378 [250]
68% (NCS)

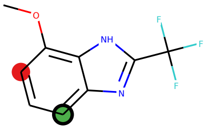

381 [328]
63% (NBS)

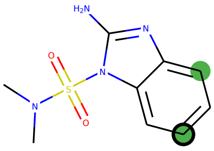

382 [75]
99% (NIS)

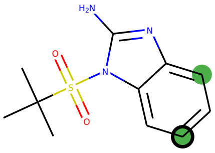

385 [145]
72% (NIS)

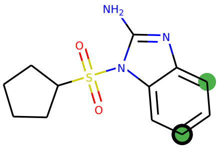

387 [145]
94% (NIS)

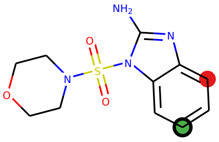

388 [79]
89% (NIS)

28 7-Azaindoles

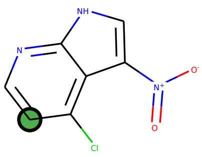

334 [194]
90% (NBS)

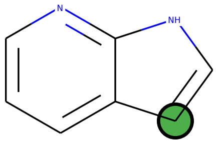

335 [44]
NBS

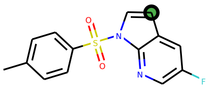

336 [175]
97% (NBS)

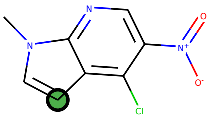

337 [69]
100% (NIS)

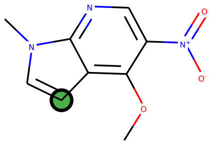

338 [69]
63% (NIS)

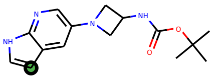

339 [220]
99% (NIS)

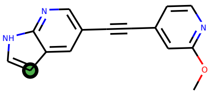

340 [280]
80% (NIS)

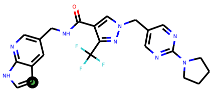

341 [384]
80% (NCS)

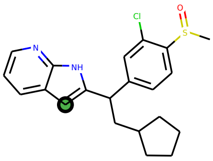

342 [334]
98% (NBS)

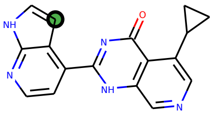

343 [156]
48% (NBS)

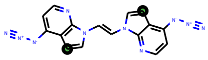

344 [427]
2xBr 49% (NBS), 2xCl 78% (NCS)

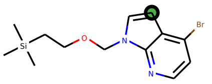

345 [171]
76% (NBS)

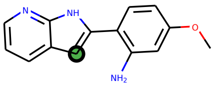

346 [337]
84% (NBS)

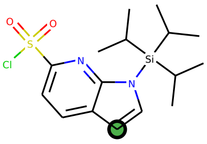

347 [151]
84% (NBS)

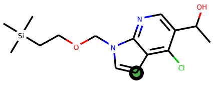

348 [203]
54% (NBS)

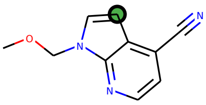

349 [32]
80% (NIS)

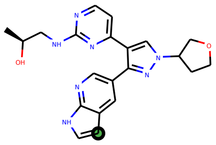

351 [139]
40% (NCS)

29 6-azaindoles

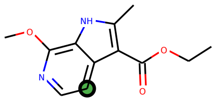

316 [14]
80% (NBS)

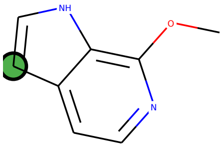

322 [202]
93% (NIS)

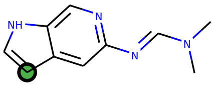

323 [73]
52% (NIS)

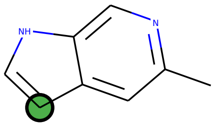

325 [248]
92% (NBS)

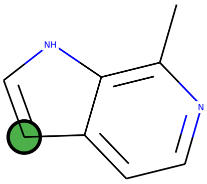

326 [248]
78% (NBS)

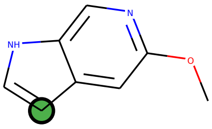

327 [154]
100% (NIS)

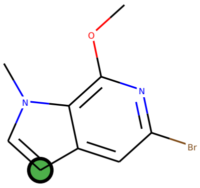

328 [423]
94% (NIS)

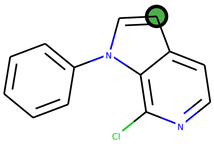

329 [116]
90% (NBS)

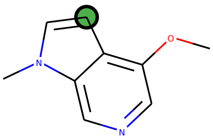

330 [36]
91% (NIS)

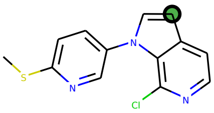

331 [389]
97% (NIS)

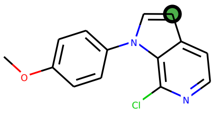

332 [389]
88% (NBS)

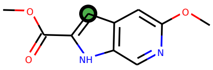

333 [29]
86% (NIS)

30 5-azaindoles

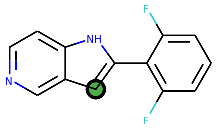

317 [419]
86% (NBS)

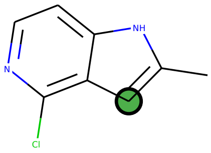

318 [409]
91% (NIS)

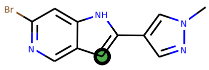

319 [37]
76% (NCS)

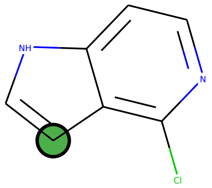

320 [229]
68% (NBS)

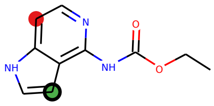

321 [77]
72% (NIS)

31 4-azaindoles

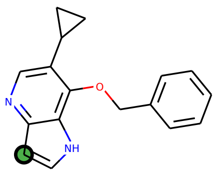

300 [445]
68% (NIS)

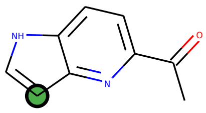

301 [273]
96% (NCS)

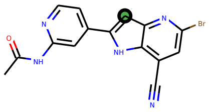

302 [339]
83% (NIS)

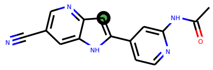

303 [339]
71% (NIS)

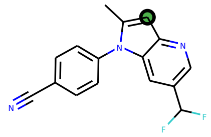

304 [140]
94% (NIS), 94% (NIS)

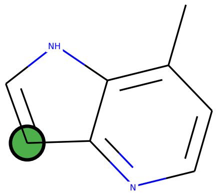

305 [118]
94% (NIS)

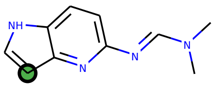

306 [73]
100% (NIS)

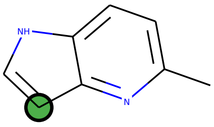

307 [24]
92% (NBS)

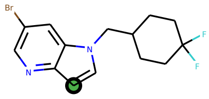

308 [449]
98% (NIS)

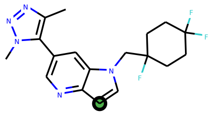

309 [449]
70% (NBS)

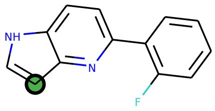

310 [305]
73% (NIS)

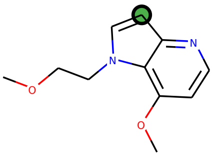

311 [119]
66% (NIS)

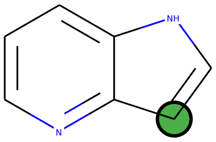

312 [31]
89% (NBS), 67% (NBS)

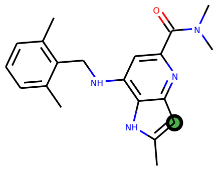

313 [335]
71% (NBS)

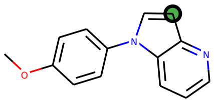

314 [389]
95% (NBS)

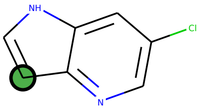

315 [142]
86% (NBS)

32 Imidazo[1,2-a]pyridines

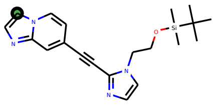

170 [64]
100% (NIS)

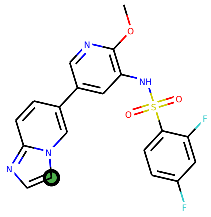

172 [432]
90% (NIS)

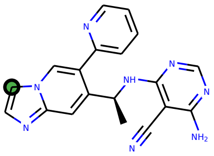

174 [284]
69% (NCS)

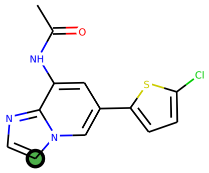

176 [160]
98% (NBS)

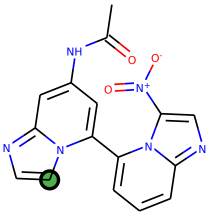

177 [197]
98% (HNO_3)

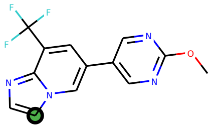

178 [375]
79% (NBS)

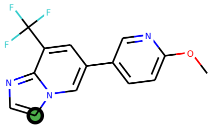

179 [375]
75% (NBS)

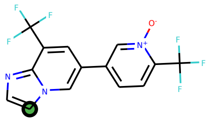

180 [375]
77% (NBS)

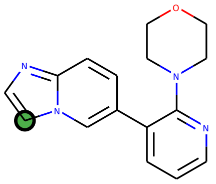

182 [375]
81% (NBS)

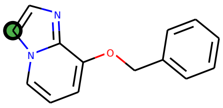

184 [163]
83% (NBS)

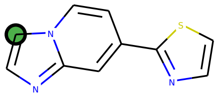

187 [48]
67% (NBS)

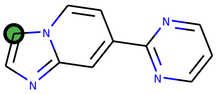

188 [371]
96% (NIS)

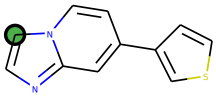

189 [172]
98% (NBS)

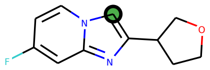

408 [199]
85% (NIS)

33 Furo[2,3-b]pyridines

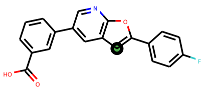

455 [348]
53% (NBS)

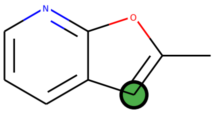

462 [316]
54% (Br_2)

34 Furo[2,3-c]pyridines

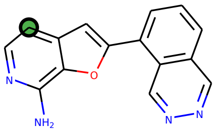

268 [209]
85% (NIS)

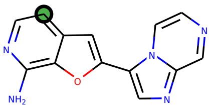

269 [209]
67% (NIS)

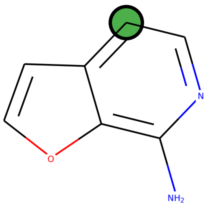

270 [209]
86% (NIS)

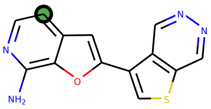

271 [209]
88% (NIS)

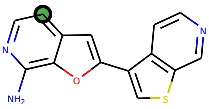

272 [209]
77% (NIS)

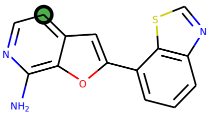

273 [209]
92% (NIS)

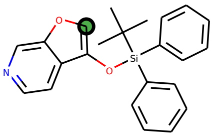

473 [90]
90% (Br_2)

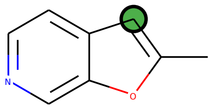

480 [435]
57% (Br_2)

35 Furo[3,2-c]pyridines

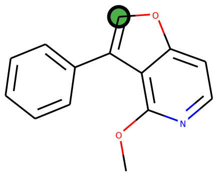

459 [57]

86% (Br_2)

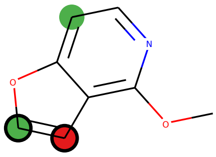

460 [57]

2xBr 99% (Br_2)

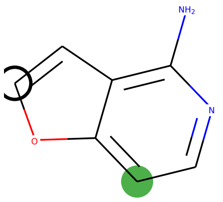

482 [404]

12, 68%

36 furo[3,2-b]pyridines

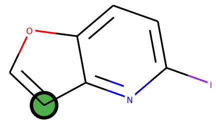

439 [338]
72% (Br_2)

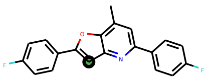

440 [436]
43% (NBS)

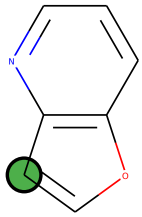

447 [357]
46% (Br_2)

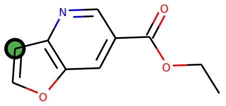

448 [357]
51% (Br_2)

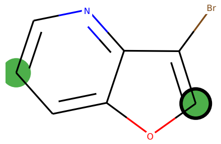

481 [434]
85% (Br_2)

37 [1,2,4]Triazolo[4,3-a]pyridines

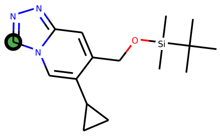

143 [262]

80% (NBS)

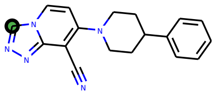

150 [122]

78% (NBS)

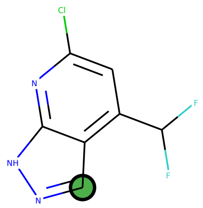

394 [22]
70% (NIS)

39 1H-Imidazo[4,5-c]pyridines

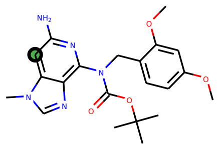

265 [350]
82% (NIS)

40 Imidazo[1,2-a]pyrazines

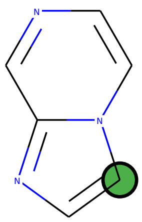

38 [200]
86% (NBS), 95% (Br_2)

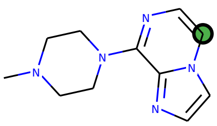

39 [301]
55% (NBS), 59% (NBS)

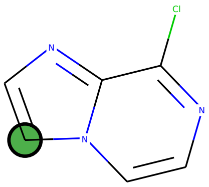

40 [53]
99% (NBS)

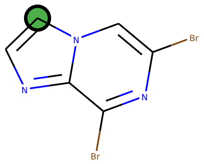

111 [82]
75% (NIS)

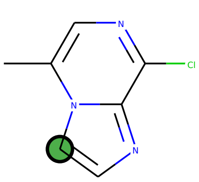

112 [292]
69% (NBS)

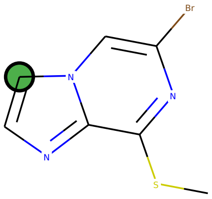

186 [258]
96% (NIS)

41 Imidazo[1,2-c]pyrimidines

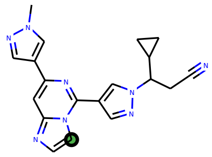

175 [392]
75% (NIS)

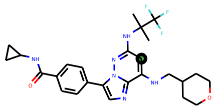

280 [268]
68% (NCS)

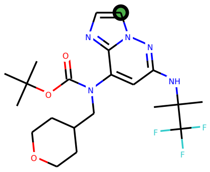

281 [214]
95% (NIS)

43 7H-Pyrrolo[2,3-d]pyrimidines

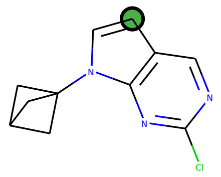

275 [134]
72% (NIS)

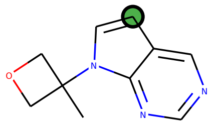

403 [28]
78% (NIS)

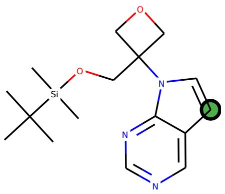

404 [28]
75% (NIS)

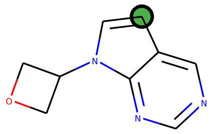

405 [28]
49% (NIS)

44 Pyrazolo[1,5-c]pyrimidines

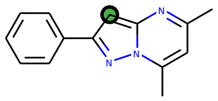

36 [193]
87% (Br_2)

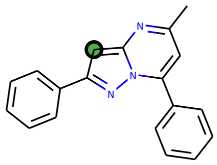

115 [279]
87% (NBS), 60% (NIS)

45 Imidazo[1,5-a]pyrazines

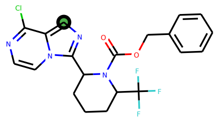

279 [144]
100% (NIS)

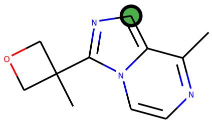

406 [296]
78% (NBS)

46 Pyrrolo[2,1-f][1,2,4]triazines

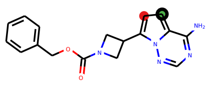

411 [11]

Br_2Me_2-hydantion, 100%

47 Oxazolo[4,5-b]pyridines

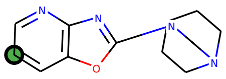

264 [329]
78% (Br_2)

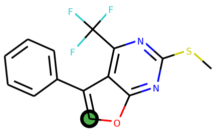

457 [57]

93% (Br_2)

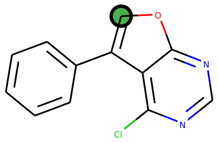

463 [135]

68% (NBS)

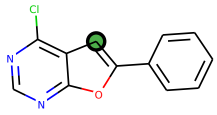

464 [135]

68% (NBS)

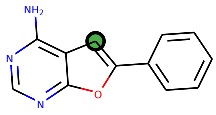

467 [403]

78% (NBS)

49 Furo[2,3-b]pyrazines

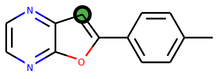

487 [2]
80% (Br_2)

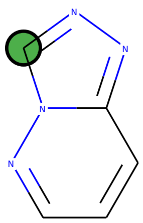

41 [263]
100% (Br_2)

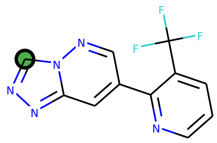

116 [88]
100% (Br_2, AcOH), 100% (Br_2, NaOAc, AcOH)

51 Pyrazolo[1,5-a][1,3,5]triazines

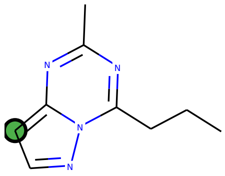

113 [374]
41% (NBS)

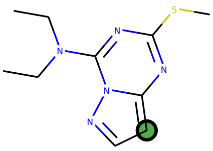

114 [374]
75% (NBS)

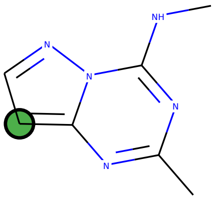

117 [418]
73% (Br_2, AcOH)

52 Naphthalenes

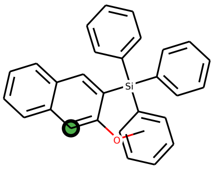

510 [257]

96% (NBS)

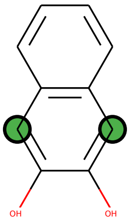

512 [453]

81% (Br_2)

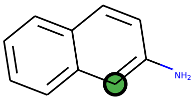

538 [332]

98% (NBS)

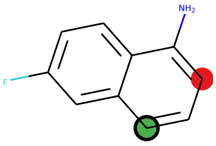

544 [433]

70% (NBS)

53 Quinolines

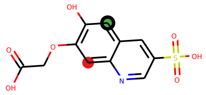

415 [152]

70% (Br_2)

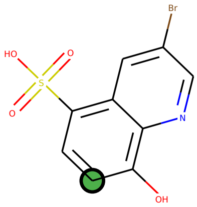

416 [183]

56% (NBS)

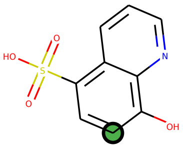

417 [183]

95% (NBS)

54 Isoquinolines

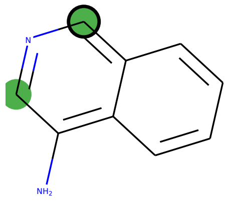

266 [42]

83% (NBS)

55 Quinoxalines

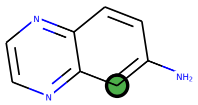

548 [45]

90% (NBS)

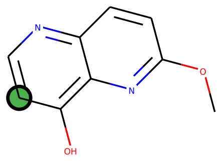

260 [91]

97% (NBS)

57 Pyrido[4,3-d]pyrimidines

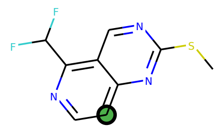

396 [92]

85% (NIS)

58 Pyrido[3,4-b]pyrazines

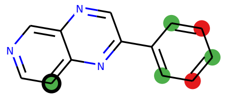

262 [297]
75% (NBS)

59 Furo[3,2-c]pyridin-4(5H)-ones

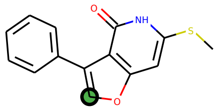

456 [57]
68% (Br_2)

60 Furo[2,3-d]pyrimidin-4(3H)-ones

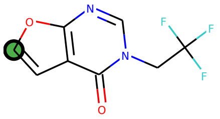

445 [201]

77% (Br_2)

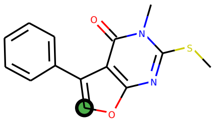

458 [57]

84% (Br_2)

61 Imidazo[1,2-a]pyrazin-8(7H)-ones

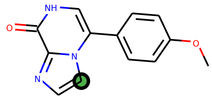

181 [223]
96% (NIS)

62 Thiazolo[5,4-b]pyridin-5(4H)-ones

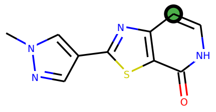

585 [74]
84% (NCS)

63 3,4-Dihydro-5H-[1,2,3]triazolo[4,5-b]pyridin-5-ones

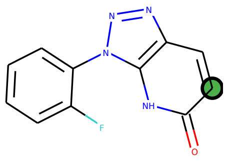

267 [86]

91% (Br_2)

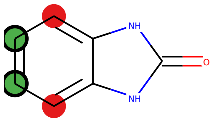

376 [147]
2xCl 97% (NCS)

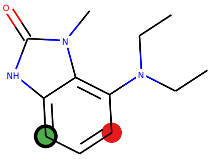

386 [309]
67% (NBS)

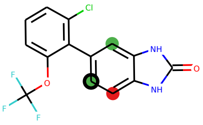

389 [354]
37% (NBS)

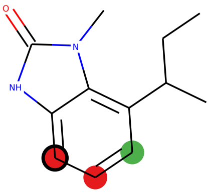

384 [35]
68% (NCS)

65 Oxazolo[4,5-b]pyridin-2(3H)-ones

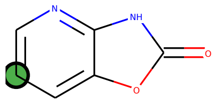

261 [408]
90% (Br_2)

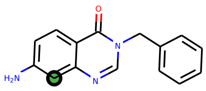

580 [204]
84% (NBS)

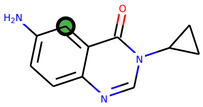

581 [441]
93% (NBS), 76% (NBS)

67 1,7-Naphthyridin-8(7H)-ones

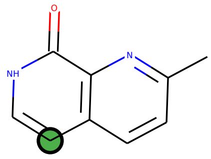

263 [221]
80% (NBS)

68 Pyrido[2,3-d]pyrimidin-7(8H)-ones

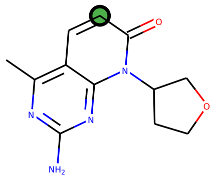

409 [41]

100% (Br_2)

69 2H-Chromen-2-ones

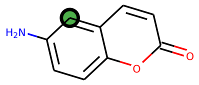

557 [107]
83% (NBS)

Section S5: Figure S3 Screenshot of webinterface

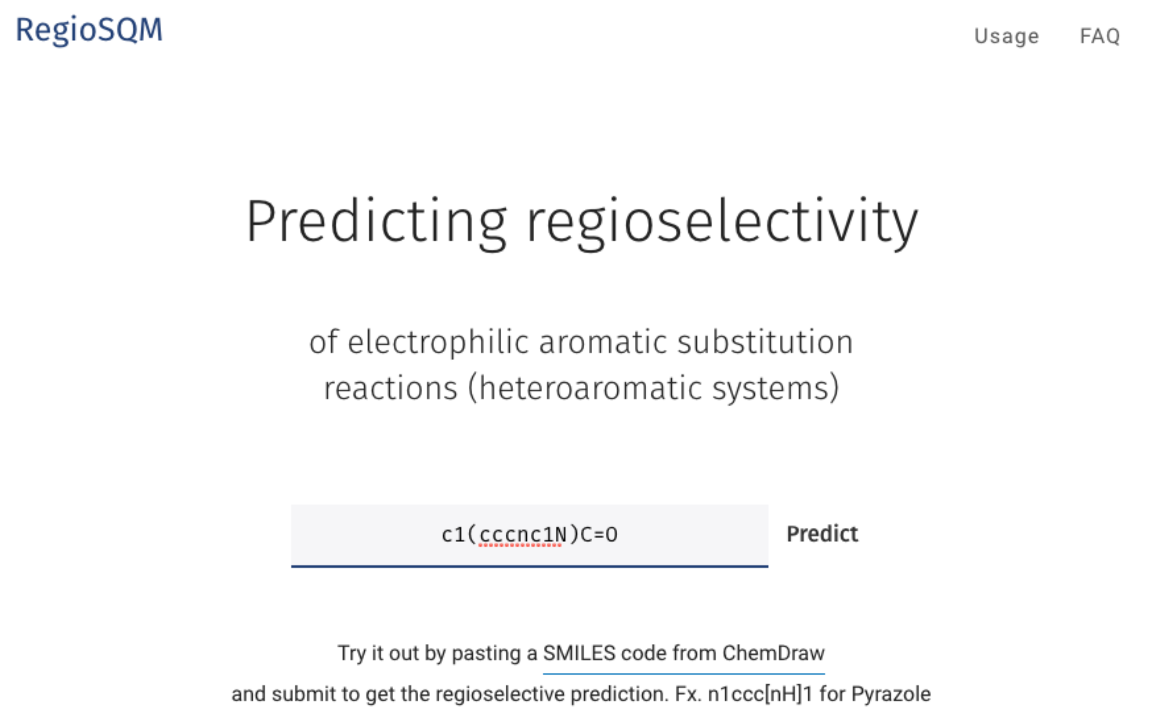

Figure 93: Screenshot of the RegioSQM webserver. The user simply enters the SMILES string of a molecule and pressed the Enter key. The SMILES string can be obtained from ChemDraw by highlighting the molecule and selecting Edit → Copy As → SMILES, which can then be pasted directly into the web interface.

References

[1] Acylamine or carbamate and amiiie or hydrazine. In *Chemistry of Heterocyclic Compounds: A Series Of Monographs*, pages 683–689. John Wiley & Sons, Inc.

[2] *Recueil des Travaux Chimiques des Pays-Bas*, 97(6):151–155, 1978.

[3] *Zhurnal Organicheskoi Khimii*, 15(12):2547–2554, 1979.

[4] *Bulletin de la Societe Chimique de France*, 132(3):290–305, 1995.

[5] *Youji Huaxue*, 27(3):370–721, 2010.

[6] Method for preparing 3-bromo-4-methoxyaniline, December 18 2013. CN Patent 102,199,099.

[7] Amino heterocyclic aromatic compound serving as c-met inhibitor, and preparation method thereof, January 8 2014. CN Patent App. CN 201,310,464,100.

[8] Compound for treatment or prevention of breast cancer, September 21 2016. CN Patent App. CN 201,610,299,350.

[9] N. Abbassi, E. M. Rakib, L. Bouissane, A. Hannioui, M. Khouili, A. El Malki, M. Benchidmi, and E. M. Essassi. Studies on the reduction of the nitro group in 4-nitroindazoles by anhydrous SnCl2in different alcohols. *Synthetic Communications*, 41(7):999–1005, mar 2011.

[10] Valentina Abet, Araceli Nunẽz, Francisco Mendicuti, Carolina Burgos, and Julio Alvarez-Builla. A new class of pyrazolopyridine nucleus with fluorescent properties, obtained through either a radical or a pd arylation pathway fromN-AzinylpyridiniumN-aminides. *The Journal of Organic Chemistry*, 73(22):8800–8807, nov 2008.

[11] F. Achebe, J. Burke, D. Cantin, J. Dixon, J. Dumas, F. Ehrgott, D. Gunn, C. Iwuagwu, W. Lee, M. Lobell, et al. Pyrrolo[2,1-f] [1,2,4] triazin-4-ylamines igf-1r kinase inhibitors for the treatment of cancer and other hyperproliferative diseases, January 3 2008. WO Patent App. PCT/US2006/043,001.

[12] J.L. Adams, T.H. Faitg, J.M. Ralph, and D.J. Silva. Pyrimidinyl-pyrazole inhibitors of aurora kinases, August 9 2007. WO Patent App. PCT/US2006/032,746.

[13] Nicholas D. Adams, Jerry L. Adams, Joelle L. Burgess, Amita M. Chaudhari, Robert A. Copeland, Carla A. Donatelli, David H. Drewry, Kelly E. Fisher, Toshihiro Hamajima, Mary Ann Hardwicke, William F. Huffman, Kristin K. Koretke-Brown, Zhihong V. Lai, Octerloney B. McDonald, Hiroko Nakamura, Ken A. Newlander, Catherine A. Oleykowski, Cynthia A. Parrish, Denis R. Patrick, Ramona Plant, Martha A. Sarpong, Kosuke Sasaki, Stanley J. Schmidt, Domingos J. Silva, David Sutton, Jun Tang, Christine S. Thompson, Peter J. Tummino, Jamin C. Wang, Hong Xiang, Jingsong Yang, and Dashyant Dhanak. Discovery of GSK1070916, a potent and selective inhibitor of aurora b/c kinase. *Journal of Medicinal Chemistry*, 53(10):3973–4001, may 2010.

[14] M. Adler, D.J. Burdick, T. CRAWFORD, M. Duplessis, S. Magnuson, C.G. Nasveschuk, F.A. Romero, Y. Tang, V.H.W. Tsui, and S. Wang. Composés thérapeutiques et leurs utilisations, August 4 2016. WO Patent App. PCT/US2016/015,450.

[15] K. ÅHLIN, P.I. Arvidsson, F. Huerta, and U. Yngve. Oxazolo[4,5-c]pyridine substituted pyrazine, May 19 2011. WO Patent App. PCT/SE2010/051,243.

[16] T. Ahlnäs. An antimicrobial composition, a method for the preparation of same, January 29 1998. WO Patent App. PCT/FI1997/000,452.

[17] T.D. Aicher, S.A. Boyd, M.J. Chicarelli, K.R. Condroski, R.F. Garrey, R.J. Hinklin, A. Singh, and T.M. Turner. 2-aminopyridine derivatives as glucokinase activators, July 31 2008. WO Patent App. PCT/US2008/051,194.

[18] T.D. Aicher, S.A. Boyd, M.J. Chicarelli, K.R. Condroski, R.J. Hinklin, and A. Singh. 2-aminopyridine analogs as glucokinase activators, November 13 2008. WO Patent App. PCT/US2008/057,543.

[19] T.D. Aicher, S.A. Boyd, M.J. Chicarelli, K.R. Condroski, R.J. Hinklin, A. Singh, T.M. Turner, and F.G. Rustam. Activateurs de glucokinases, August 9 2007. WO Patent App. PCT/US2007/001,956.

[20] R.J. Albers, R.L. Harris, D. Huang, B.G. Lee, M.M.D. Mederos, D.S. Mortensen, G.K. Packard, P.W. Papa, J.S. Parnes, S. Perrin-Ninkovic, et al. Heteroaryl compounds, compositions thereof, and their use as protein kinase inhibitors, July 3 2008. WO Patent App. PCT/US2007/022,374.

[21] M.J. Alberti, P. Bamborough, D.H. Drewry, and D.D. Miller. Chemical compounds, February 17 2005. WO Patent App. PCT/US2004/008,301.

[22] C. Alcouffe. Dérivés de pyrazolopyridine à utiliser dans le traitement du cancer de la vessie, December 18 2014. WO Patent App. PCT/EP2014/062,456.

[23] Jo Alen and Liliana Dobrzańska. 3-benzyl-5-bromopyrazin-2(1h)-one. *Acta Crystallographica Section E Structure Reports Online*, 64(3):o547–o547, feb 2008.

[24] Juan Antonio Alonso, Miriam Andrés, Mónica Bravo, Maria Antonia Buil, Marta Calbet, Jordi Castro, Paul R. Eastwood, Cristina Esteve, Manel Ferrer, Pilar Forns, Elena Gómez, Jacob González, Estrella Lozoya, Marta Mir, Imma Moreno, Silvia Petit, Richard S. Roberts, Sara Sevilla, Bernat Vidal, Laura Vidal, and Pere Vilaseca. Structure–activity relationships (SAR) and structure–kinetic relationships (SKR) of bicyclic heteroaromatic acetic acids as potent CRTh2 antagonists III: The role of a hydrogen-bond acceptor in long receptor residence times. *Bioorganic & Medicinal Chemistry Letters*, 24(21):5127–5133, nov 2014.

[25] M.D. Altman, B.M. Andresen, K.L. Arrington, K.K. Childers, F.M.E. Di, A. Donofrio, J.M. Ellis, C. Fischer, D.J. Guerin, A.M. Haidle, et al. Aminopyrimidines as syk inhibitors, June 23 2011. WO Patent App. PCT/US2010/060,454.

[26] E. Alvira, R.M. Blevis-Bal, T.L. Boehm, B. Devadas, R. Devraj, R.C. Durley, B.S. Hickory, J. Hitchcock, K.D. Jerome, S. Liu, et al. Substituted pyridinones, August 4 2005. WO Patent App. PCT/US2004/026,193.

[27] N. Amishiro, T. Atsumi, T. Nakazato, H. Umehara, H. Fujihara, F. Shinohara, J. Funahashi, J. Yamamoto, and K. Yagi. Jak inhibitor, April 24 2008. WO Patent App. PCT/JP2007/070,245.

[28] M.D. Andrews, S.K. Bagal, K.R. Gibson, K. OMOTO, T. Ryckmans, S.E. Skerratt, and P.A. Stupple. Pyrrolo [2, 3 -d] pyrimidine derivatives as inhibitors of tropomyosin- related kinases, October 11 2012. WO Patent App. PCT/IB2012/051,363.

[29] G.N. Anilkumar, S.B. Rosenblum, S. Venkatraman, F.G. Njoroge, and J.A. Kozlowski. 2,3-substituted azaindole derivatives for treating viral infections, March 12 2009. WO Patent App. PCT/US2008/010,149.

[30] Atsushi Aoyama, Kaori Endo-Umeda, Kenji Kishida, Kenji Ohgane, Tomomi Noguchi-Yachide, Hiroshi Aoyama, Minoru Ishikawa, Hiroyuki Miyachi, Makoto Makishima, and Yuichi Hashimoto. Design, synthesis, and biological evaluation of novel transrepression-selective liver x receptor (LXR) ligands with 5,11-dihydro-5-methyl-11-methylene-6h-dibenz[b,e]azepin-6-one skeleton. *Journal of Medicinal Chemistry*, 55(17):7360–7377, sep 2012.

[31] N. Arai and I. Hiroshi. Memory function body, particle forming method therefor and, memory device, semiconductor device, and electronic equipment having the memory function body, January 3 2008. US Patent App. 11/674,529.

[32] C. Arendt, C. Barberis, M. Levit, and T.N. Majid. Azaindole derivatives, their preparation and their therapeutic application, June 23 2011. WO Patent App. PCT/US2010/060,910.

[33] J. Arndt, T. Chan, K. Guckian, G. Kumaravel, W.C. Lee, E.Y.S. Lin, D. Scott, L. Sun, J. Thomas, V.K. Van, et al. Heterocyclic compounds useful as pdk1 inhibitors, April 14 2011. WO Patent App. PCT/US2010/051,517.

[34] A. Aronov, R.S. Bethiel, R.J. Davies, D.D. Deininger, L. Farmer, R. Grey, D. Jacobs, D.J. Lauffer, B. Ledford, P. Li, et al. C-met protein kinase inhibitors for the treatment of proliferative disorders, December 13 2007. WO Patent App. PCT/US2007/007,016.

[35] K. Aso, S.Y. Cho, C.P. Corrette, A.C. Gyorkos, M. Gyoten, S.A. Pratt, and C.S. Siedem. Fused heterocyclic compounds, March 1 2007. WO Patent App. PCT/US2006/015,646.

[36] M. Atobe, K. Nakabayashi, K. Machida, and S. Takeda. Polymerization fluid, method for producing the polymerization fluid, transparent film and transparent electrode made from the polymerization fluid, February 14 2013. US Patent App. 13/582,282.

[37] B. Atrash, V. Bavetsias, J. Blagg, S.G.A. NAUD, and P.W. Sheldrake. Pyrrolopyridineamino derivatives as mps1 inhibitors, September 20 2012. WO Patent App. PCT/GB2012/050,564.

[38] Karine Audouze, Elsebet Østergaard Nielsen, Gunnar M. Olsen, Philip Ahring, Tino Dyhring Jørgensen, Dan Peters, Tommy Liljefors, and Thomas Balle. New ligands with affinity for the α4β2subtype of nicotinic acetylcholine receptors. synthesis, receptor binding, and 3d-QSAR modeling. *Journal of Medicinal Chemistry*, 49(11):3159–3171, jun 2006.

[39] D.K. Baeschlin, N. Ostermann, F. Gessier, F. Sirockin, and K. Namoto. Organic compounds, October 11 2007. WO Patent App. PCT/EP2007/053,064.

[40] J.T. BAGDANOFF, Y. Ding, W. Han, Z. Huang, Q. JIANG, J.X. Jin, X. KOU, P. Lee, M. Lindvall, Z. MIN, et al. Aminoheteroaryl benzamides as kinase inhibitors, May 7 2015. WO Patent App. PCT/US2014/062,913.

[41] T.G. Baik, S. Ma, C.A. Buhr, and J.M. Nuss. Inhibiteurs de pyridopyrimidinone de la pi3 kinase α et de la mtor, April 8 2010. WO Patent App. PCT/US2009/058,881.

[42] J. M. Bakke and J. Riha. Preparation of 4-substituted 3-amino-2-chloropyridines, synthesis of a nevirapine analogue. *Journal of Heterocyclic Chemistry*, 38(1):99–104, jan 2001.

[43] S.K. Ballentine, J. Chen, C.R. Illig, S.K. Meegalla, M.J. Rudolph, M.J. Wall, and K.J. Wilson. Method of inhibiting c kit kinase, March 6 2008. WO Patent App. PCT/US2007/066,985.

[44] Christine B. Baltus, Radek Jorda, Christophe Marot, Karel Berka, Václav Bazgier, Vladimír Kryštof, Gildas Prié, and Marie-Claude Viaud-Massuard. Synthesis, biological evaluation and molecular modeling of a novel series of 7-azaindole based tri-heterocyclic compounds as potent CDK2/cyclin e inhibitors. *European Journal of Medicinal Chemistry*, 108:701–719, jan 2016.

[45] Babasaheb Pandurang Bandgar, Sanjay Suresh Sawant, and S. M. Salman Jaweed Mukarram. *Indian Pat. Appl.*, chapter A process for the preparation of brimonidine., page 27pp. Number IN2011MU03306A. India ., Jun 2013. Copyright (C) 2017 American Chemical Society (ACS). All Rights Reserved.

[46] H. Banno, M. Hirose, O. Kurasawa, S.P. Langston, H. Mizutani, Z. Shi, I. Visiers, T.J. Vos, and S. Vyskocil. Heteroaryls and their use as pi3k inhibitors, August 12 2010. WO Patent App. PCT/US2010/000,234.

[47] Giovanna Barbarella, Massimo Zambianchi, Alfredo Ventola, Eduardo Fabiano, Fabio Della Sala, Giuseppe Gigli, Marco Anni, Andrea Bolognesi, Letizia Polito, Marina Naldi, and Massimo Capobianco. Bright OligothiopheneN-succinimidyl esters for efficient fluorescent labeling of proteins and oligonucleotides. *Bioconjugate Chemistry*, 17(1):58–67, jan 2006.

[48] D.A. Barda, T.P. Burkholder, J.R. Clayton, Y. Hao, P.C. Heath, J.R. Henry, J.M. Knobeloch, D. Mendel, J.A. Mclean, D.M. Remick, et al. Imidazo (1, 2-a) pyridine compounds as vegf-r2 inhibitors, August 31 2006. WO Patent App. PCT/US2006/006,283.

[49] L. Bärfacker, R. Kast, N. Griebenow, H. Meier, P. Kolkhof, B. Albrecht-Küpper, A. Nitsche, N. Teusch, J.P. Stasch, and D. Schneider. Substituted 5-aminopyrazoles and use thereof, April 22 2010. US Patent App. 12/540,657.

[50] B.C. Barlaam, J.F. Bower, B. Delouvrie, G. Fairley, C.S. Harris, C. Lambert, G. Ouvry, and J.J.G. Winter. Pyridine and pyrazine derivatives useful in the treatment of cell proliferative disorders, April 30 2009. WO Patent App. PCT/GB2008/050,974.

[51] B.C. Barlaam and K.G. Pike. Imidazo[4,5-c]quinolin-2-one compounds and their use in treating cancer, November 12 2015. WO Patent App. PCT/GB2015/051,312.

[52] Bernard Barlaam, Mike Fennell, Hervé Germain, Tim Green, Laurent Hennequin, Rémy Morgentin, Annie Olivier, Patrick Plé, Michel Vautier, and Gerard Costello. New heterocyclic analogues of 4-(2-chloro-5-methoxyanilino)quinazolines as potent and selective c-src kinase inhibitors. *Bioorganic & Medicinal Chemistry Letters*, 15(24):5446–5449, dec 2005.

[53] José Manuel Bartolomé-Nebreda, Francisca Delgado, María Luz Martín-Martín, Carlos M. Martínez-Viturro, Joaquín Pastor, Han Min Tong, Laura Iturrino, Gregor J. Macdonald, Wendy Sanderson, Anton Megens, Xavier Langlois, Marijke Somers, Greet Vanhoof, and Susana Conde-Ceide. Discovery of a potent, selective, and orally active phosphodiesterase 10a inhibitor for the potential treatment of schizophrenia. *Journal of Medicinal Chemistry*, 57(10):4196–4212, may 2014.

[54] Gregory S Basarab, John I Manchester, Shanta Bist, P Ann Boriack-Sjodin, Brian Dangel, Ruth Illingworth, Brian A Sherer, Shubha Sriram, Maria Uria-Nickelsen, and Ann E Eakin. Fragment-to-hit-to-lead discovery of a novel pyridylurea scaffold of atp competitive dual targeting type ii topoisomerase inhibiting antibacterial agents. *Journal of medicinal chemistry*, 56(21):8712–8735, 2013.

[55] M. Bastos, J. Lata, and J.D. Edelson. Uses of natriuretic peptide constructs, December 29 2011. WO Patent App. PCT/US2011/033,366.

[56] M. Bejugam, S. Hosahalli, and N. Mahalingam. Trisubstituted heterocyclic derivatives as ror gamma modulators, August 21 2014. WO Patent App. PCT/IB2014/058,957.

[57] M.P. Bell, C.R. O'dowd, J.S.S. Rountree, G.P. Trevitt, T. Harrison, and M.M. Mcfarland. Akt / pkb inhibitors, May 12 2011. WO Patent App. PCT/GB2010/002,033.

[58] Fabio Bellina, Silvia Cauteruccio, Annarita Di Fiore, Chiara Marchetti, and Renzo Rossi. Highly selective synthesis of 4 (5)-aryl-, 2, 4 (5)-diaryl-, and 4, 5-diaryl-1h-imidazoles via pd-catalyzed direct c-5 arylation of 1-benzyl-1h-imidazole. *Tetrahedron*, 64(26):6060–6072, 2008.

[59] Fabio Bellina, Silvia Cauteruccio, Annarita Di Fiore, and Renzo Rossi. Regioselective synthesis of 4, 5-diaryl-1-methyl-1h-imidazoles including highly cytotoxic derivatives by pd-catalyzed direct c-5 arylation of 1-methyl-1h-imidazole with aryl bromides. *European Journal of Organic Chemistry*, 2008(32):5436–5445, 2008.

[60] Fabio Bellina, Silvia Cauteruccio, Annarita Di Fiore, Chiara Marchetti, and Renzo Rossi. Highly selective synthesis of 4(5)-aryl-, 2,4(5)-diaryl-, and 4,5-diaryl-1h-imidazoles via pd-catalyzed direct c-5 arylation of 1-benzyl-1h-imidazole. *Tetrahedron*, 64(26):6060–6072, jun 2008.

[61] Fabio Bellina, Silvia Cauteruccio, Annarita Di Fiore, and Renzo Rossi. Regioselective synthesis of 4,5-diaryl-1-methyl-1h-imidazoles including highly cytotoxic derivatives by pd-catalyzed direct c-5 arylation of 1-methyl-1h-imidazole with aryl bromides. *European Journal of Organic Chemistry*, 2008(32):5436–5445, nov 2008.

[62] Fabio Bellina, Marco Lessi, Giulia Marianetti, and Alessandro Panattoni. Highly regioselective c-5 alkynylation of imidazoles by one-pot sequential bromination and sonogashira cross coupling. *Tetrahedron Letters*, 56(25):3855–3857, jun 2015.

[63] K.E. Benenato, A.L. Choy, M.R. Hale, P. Hill, V. Marone, and M. Miller. Hydroxamic acid derivatives as gram-negative antibacterial agents, September 10 2010. WO Patent App. PCT/GB2010/050,351.

[64] V. Berdini, M.G. Carr, M.S. Congreve, M. Frederickson, C.M. Griffiths-Jones, C.C.F. Hamlett, A. Madin, C.W. Murray, R.K. Benning, G. Saxty, et al. Imidazopyridine derivatives as inhibitors of receptor tyrosine kinases, December 17 2009. WO Patent App. PCT/EP2009/057,318.

[65] Jan Bergman, Jeffrey Mason, and Tomasz Janosik. A new approach to methoxyisatins leading to the total synthesis of ophiuroidine and other hydroxytryptanthrins. *Synthesis*, 2009(21):3642–3648, sep 2009.

[66] J.A. Bilotta, Z. Chen, E. Chin, Q. Ding, S.D. Erickson, S.D. Gabriel, K. Klumpp, H. Ma, E. Mertz, J.M. Plancher, et al. Triazole compounds as antivirals, January 9 2014. WO Patent App. PCT/EP2013/063,980.

[67] A. Binggeli, A.D. Christ, L.G.G. Green, W. Guba, H.P. Maerki, R.E. Martin, and P. Mohr. Benzothiazole, thiazolopyridine, benzooxazole and oxazolopyridine derivatives as antidiabetic compounds, September 14 2006. WO Patent App. PCT/EP2006/001,843.

[68] C. Bissantz, B. DHURWASULU, U. Grether, A. HAZRA, P. Hebeisen, S. Roever, and M. Rogers-Evans. Novel pyrazine derivatives, May 2 2013. WO Patent App. PCT/EP2012/071,093.

[69] R.K. Blackman, K.P. Foley, and D. Proia. Cancer therapy using a combination of a hsp90 inhibitory compounds and a egfr inhibitor, May 5 2016. US Patent App. 14/930,782.

[70] J.F. Blake, S.A. Boyd, F. Cohen, M.J. De, K.C. Fong, J.J. Gaudino, T. Kaplan, A.L. Marlow, J. Seo, A.A. Thomas, et al. Heterobicyclic pyrazole compounds and methods of use, September 13 2007. WO Patent App. PCT/US2007/005,583.

[71] J. Blank, G. Bold, S. Cotesta, V. Guagnano, H. Rueeger, and A. Vaupel. Imidazopyrrolidinone derivatives and their use in the treatment of disease, December 4 2014. WO Patent App. PCT/IB2014/061,715.

[72] N. Blaquiere, J. Burch, G. Castanedo, J.A. FENG, B. Hu, X. LIN, S. Staben, G. Wu, and P.W. Yuen. Alkynyl alcohols and methods of use, February 26 2015. WO Patent App. PCT/EP2014/067,873.

[73] J.R. BLINN, A.C. FLICK, G.M. WENNERSTÅL, P. Jones, N. Kaila, J.R. Kiefer, R.G. Kurumbail, S.R. Mente, M.J. Meyers, M.E. Schnute, et al. Heterobicycloaryl rorc2 inhibitors and methods of use thereof, May 14 2015. WO Patent App. PCT/IB2014/063,383.

[74] D. Bonafoux, H.M. Davis, K.E. Frank, M.M. Friedman, J.M. HEROLD, M.Z. Hoemann, R. Huntley, A. Osuma, G. Sheppard, G.K. SOMAL, et al. Primary carboxamides as btk inhibitors, December 31 2014. WO Patent App. PCT/US2014/044,247.

[75] R. Bonjouklian, R.D. Dally, D.A. De, P.C.M.F. Del, C. Dominguez-Fernandez, A.C. Jaramillo, D.U.G.B. Lopez, S.C. Montero, and T.A. Shepherd. Kinase inhibitors, September 1 2005. WO Patent App. PCT/US2005/000,024.

[76] M El Borai, A. H. Moustafa, M Anwar, and F. I. Abdel Hay. *Polym. J. Chem.*, 55:1659 – 1665, 1981.

[77] R. Borzilleri, K. Kim, and Z. Cai. Bicyclic heterocycles as kinase inhibitors, October 27 2005. US Patent App. 11/113,838.

[78] Maxence Bos and Emmanuel Riguet. Synthesis of chiral γ-lactones by one-pot sequential enantioselective organocatalytic michael addition of boronic acids and diastereoselective intramolecular passerini reaction. *The Journal of Organic Chemistry*, 79(22):10881–10889, nov 2014.

[79] J. Botyanszki, S.H. Dickerson, M.R. Leivers, X. Li, R.B. Mcfadyen, A.M. REDMAN, J.B. SHOTWELL, and J. Xue. Benzimidazole derivatives as antiviral agents, February 28 2013. WO Patent App. PCT/US2012/042,558.

[80] Abderrahman El Bouakher, Hassan Allouchi, Isabelle Abrunhosa-Thomas, Yves Troin, and Gérald Guillaumet. Suzuki-miyaura reactions of halospirooxindole derivatives. *European Journal of Organic Chemistry*, 2015(16):3450–3461, apr 2015.

[81] B. BOUBIA, M. Barth, J. Binet, P. Dodey, C. Legendre, and O. Poupardin-Olivier. Pyrrolopyridine derivatives and use of same as ppar receptor modulators, March 8 2007. WO Patent App. PCT/FR2006/050,827.

[82] Nathalie Bouloc, Jonathan M. Large, Magda Kosmopoulou, Chongbo Sun, Amir Faisal, Mizio Matteucci, Jóhannes Reynisson, Nathan Brown, Butrus Atrash, Julian Blagg, Edward McDonald, Spiros Linardopoulos, Richard Bayliss, and Vassilios Bavetsias. Structure-based design of imidazo[1,2-a]pyrazine derivatives as selective inhibitors of aurora-a kinase in cells. *Bioorganic & Medicinal Chemistry Letters*, 20(20):5988–5993, oct 2010.

[83] M.L. Boys, R.K. Delisle, E.J. Hicken, A.L. Kennedy, D.A. Mareska, F.P. Marmsater, M.C. Munson, B. Newhouse, B. Rast, J.P. Rizzi, et al. Substituted n-(1h-indazol-4-yl)imidazo[1,2-a]pyridine-3-carboxamide compounds as type iii receptor tyrosine kinase inhibitors, June 21 2012. WO Patent App. PCT/US2011/064,549.

[84] Danuta Branowska, Ewa Olender, and Andrzej Rykowski. Palladium-catalyzed cross-coupling of 5-acyl and 5-formyl-1,2,4-triazines and their derivatives with heteroaromatic tin compounds. *Tetrahedron*, 70(31):4697–4702, aug 2014.

[85] M.A. Brodney, E.M. BECK, C.R. BUTLER, L. Zhang, B.T. O'neill, G. BARREIRO, E.A. LACHAPELLE, and B.N. Rogers. 2-amino-6-methyl-4,4a,5,6-tetrahydropyrano[3,4-d][1,3]thiazin-8a(8h)-yl-1,3-thiazol-4-yl amides, October 15 2015. WO Patent App. PCT/IB2015/052,279.

[86] H.B. Broughton, P.J.L. Castro, I.J. Collins, and L.J. Street. Triazolo-pyridine derivatives as ligands for gaba receptors, December 23 1999. WO Patent App. PCT/GB1999/001,838.

[87] D. J. Brown and P. Waring. *Aust. J. Chem.*, 31(6):1391–1395, 1978.

[88] R.E. Brown, F. Burkamp, V.A. Doughty, S.R. Fletcher, G.J. Hollingworth, B.A. Jones, and T.J. Sparey. Substituted amino heterocycles as vr-1 antagonists for treating pain, September 2 2004. WO Patent App. PCT/GB2004/000,702.

[89] B.O. Buckman, J.B. Nicholas, J.Y. Ramphal, K. Emayan, and S.D. Seiwert. Anti-fibrotic pyridinones, April 3 2014. US Patent App. 13/799,727.

[90] A.J. Buckmelter, J.P. Lyssikatos, G. Miknis, L. Ren, and S.M. Wenglowsky. Raf inhibitor compounds and methods of use thereof, April 10 2008. WO Patent App. PCT/US2007/077,411.

[91] M.E. Bunnage, A.S. Cook, J.J. Cui, K.N. Dack, J.G. Deal, D. Gu, M. He, P.S. Johnson, T.W. Johnson, P.T.Q. Le, et al. Heterocyclic derivatives as alk inhibitors, November 10 2011. WO Patent App. PCT/IB2011/051,981.

[92] L. Burgdorf, D. Kuhn, T. Ross, and C. Deutsch. Pyridopyrimidine derivatives as protein kinase inhibitors, February 13 2014. WO Patent App. PCT/EP2013/002,032.

[93] Matthew T. Burger, Sabina Pecchi, Allan Wagman, Zhi-Jie Ni, Mark Knapp, Thomas Hendrickson, Gordana Atallah, Keith Pfister, Yanchen Zhang, Sarah Bartulis, Kelly Frazier, Simon Ng, Aaron Smith, Joelle Verhagen, Joshua Haznedar, Kay Huh, Ed Iwanowicz, Xiaohua Xin, Daniel Menezes, Hanne Merritt, Isabelle Lee, Marion Wiesmann, Susan Kaufman, Kenneth Crawford, Michael Chin, Dirksen Bussiere, Kevin Shoemaker, Isabel Zaror, Sauveur-Michel Maira, and Charles F. Voliva. Identification of NVP-BKM120 as a potent, selective, orally bioavailable class i PI3 kinase inhibitor for treating cancer. *ACS Medicinal Chemistry Letters*, 2(10):774–779, oct 2011.

[94] Carolina Burgos, Francisca Delgado, J. Luis García-Navío, M. Luisa Izquierdo, and Julio Alvarez-Builla. Halogenation of pyridinium-n-(2′-pyridyl)aminide: An easy synthesis of halo-2-aminopyridines. *Tetrahedron*, 51(31):8649–8654, jul 1995.

[95] Alexey N. Butkevich, Gyuzel Yu. Mitronova, Sven C. Sidenstein, Jessica L. Klocke, Dirk Kamin, Dirk N. H. Meineke, Elisa D'Este, Philip-Tobias Kraemer, Johann G. Danzl, Vladimir N. Belov, and Stefan W. Hell. Fluorescent rhodamines and fluorogenic carbopyronines for super-resolution STED microscopy in living cells. *Angewandte Chemie International Edition*, 55(10):3290–3294, feb 2016.

[96] Z.R. Cai, Z. Du, M. Ji, H. Jin, C.U. Kim, M.R. Mish, B.W. Phillips, H.J. Pyun, X.C. Sheng, Q. Wu, et al. Quinoline and isoquinoline derivatives as inhibitors of hepatitis c virus, January 26 2012. WO Patent App. PCT/US2011/039,813.

[97] C.G. Caldwell, Y.C. Chiang, C. Dorn, P. Finke, J. Hale, M. MacCoss, S. Mills, and A. Robichaud. Phenyl spiroethercycloalkyl tachykinin receptor antagonists, March 2 1999. US Patent 5,877,191.

[98] Victoria Cañibano, Justo F. Rodríguez, Mercedes Santos, M. Ascensión Sanz-Tejedor, M. Carmen Carreño, Gema González, and José L. García-Ruano. Mild regioselective halogenation of activated pyridines with n-bromosuccinimide. *Synthesis*, 2001(14):2175–2179, 2001.

[99] Yuanyuan Cao, Yu Zhang, Shaotong Wu, Quanzhi Yang, Xuefeng Sun, Jianxiong Zhao, Fen Pei, Ying Guo, Chao Tian, Zhili Zhang, Haining Wang, Liying Ma, Junyi Liu, and Xiaowei Wang. Synthesis and biological evaluation of pyridinone analogues as novel potent HIV-1 NNRTIs. *Bioorganic & Medicinal Chemistry*, 23(1):149–159, jan 2015.

[100] S. Carboni, A. Da Settimo, P. L. Ferrarini, G. Primofiore, O. Livi, V. Menichetti, M. Del Tacca, E. Martinotti, C. Bernardini, and Bertelli. *A. Eur. J. Med. Chem.*, 17(2):159–164, 1984.

[101] R.M. Carrascal, F.J. Gracia, V.G. Matassa, S.L.M. Pages, and B.E. Terricabras. Imidazopyridine derivatives as pi3k inhibitors, November 1 2012. WO Patent App. PCT/EP2012/057,674.

[102] R.L. Casaubon, R. Narayan, C. Oalmann, and C.B. Vu. Substituted bicyclic azaheterocycles and analogues as sirtuin modulators, October 23 2014. WO Patent App. PCT/US2012/061,015.

[103] Rajadurai Chandrasekar, Supratim Basak, and Pramiti Hui. Regioselective, one-pot syntheses of symmetrically and unsymmetrically halogenated 2′,6′-bispyrazolylpyridines. *Synthesis*, 2009(23):4042–4048, oct 2009.

[104] J.D. Charrier, S.J. Durrant, J. Pinder, P.M. Reaper, P.H. Storck, A.N. Virani, and S.C. Young. Compounds useful as inhibitors of atr kinase, March 1 2012. WO Patent App. PCT/US2011/036,243.

[105] R.W. CHAU, C.A. Cullis, M.O. Duffey, K.E. GIPSON, Y. Hu, G. Li, M.D. Sintchak, and T.J. Vos. Heteroaryls and uses thereof, June 27 2013. WO Patent App. PCT/US2012/070,988.

[106] R.W. CHAU, C.A. Cullis, M.O. Duffey, K.E. GIPSON, Y. Hu, G. Li, M.D. Sintchak, and T.J. Vos. Heteroaryls and uses thereof, June 27 2013. WO Patent App. PCT/US2012/070,988.

[107] Lei Chen, Tai-Shan Hu, and Zhu-Jun Yao. Development of new pyrrolocoumarin derivatives with satisfactory fluorescent properties and notably large stokes shifts. *European Journal of Organic Chemistry*, 2008(36):6175–6182, dec 2008.

[108] T. Chen, D. Levin, and S. Puppali. Pharmaceutical compositions comprising monoterpenes, January 9 2013. EP Patent App. EP20,110,751,368.

[109] X. Chen, C.L. Cioffi, S.R. Dinn, A.M. Escribano, M.C. Fernandez, T. Fields, R.J. Herr, N.B. Mantlo, D.L.N.E.M. Martin, A.I. Mateo-Herranz, et al. Compounds and methods for treating dyslipidemia, January 5 2006. WO Patent App. PCT/US2005/022,389.

[110] Z. Chen, C.H. Hamdouchi, E.J. Hembre, P.A. Hipskind, J.K. Myers, T. Takakuwa, and J.L. Toth. Thiazole pyrazolopyrimidines as crf1 receptor antagonists, March 27 2008. WO Patent App. PCT/US2007/078,605.

[111] Zhilong Chen, Zhaobin Wang, and Jianwei Sun. Catalytic enantioselective synthesis of tetrahydroisoquinolines and their analogues bearing a c4 stereocenter: Formal synthesis of (+)-(8s,13r)-cyclocelabenzine. *Chemistry - A European Journal*, 19(26):8426–8430, may 2013.

[112] Chi-Jui Chiang, Jyh-Chien Chen, Hsiang-Yen Tsao, Kuan-Yi Wu, and Chien-Lung Wang. Ambient-stable, annealing-free, and ambipolar organic field-effect transistors based on solution-processable poly(2,2′-bis(trifluoromethyl)biphenyl-alt-2,5-divinylthiophene) without long alkyl side chains. *Advanced Functional Materials*, 25(4):606–614, dec 2014.

[113] Young Shin Cho, Maria Borland, Christopher Brain, Christine H.-T. Chen, Hong Cheng, Rajiv Chopra, Kristy Chung, James Groarke, Guo He, Ying Hou, Sunkyu Kim, Steven Kovats, Yipin Lu, Marc O'Reilly, Junqing Shen, Troy Smith, Gary Trakshel, Markus Vögtle, Mei Xu, Ming Xu, and Moo Je Sung. 4-(pyrazol-4-yl)-pyrimidines as selective inhibitors of cyclin-dependent kinase 4/6. *Journal of Medicinal Chemistry*, 53(22):7938–7957, nov 2010.

[114] Harry R. Chobanian, Yan Guo, Ping Liu, Marc D. Chioda, Selena Fung, Thomas J. Lanza, Linda Chang, Raman K. Bakshi, James P. Dellureficio, Qingmei Hong, Mark McLaughlin, Kevin M. Belyk, Shane W. Krska, Amanda K. Makarewicz, Elliot J. Martel, Joseph F. Leone, Lisa Frey, Bindhu Karanam, Maria Madeira, Raul Alvaro, Joyce Shuman, Gino Salituro, Jenna L. Terebetski, Nina Jochnowitz, Shruti Mistry, Erin McGowan, Richard Hajdu, Mark Rosenbach, Catherine Abbadie, Jessica P. Alexander, Lin-Lin Shiao, Kathleen M. Sullivan, Ravi P. Nargund, Matthew J. Wyvratt, Linus S. Lin, and Robert J. DeVita. Discovery of MK-4409, a novel oxazole FAAH inhibitor for the treatment of inflammatory and neuropathic pain. *ACS Medicinal Chemistry Letters*, 5(6):717–721, jun 2014.

[115] H. Choi, J. Lee, C.G. LEE, N. Ha, S.K. Seo, S. Lee, and S.M. Lee. N-acylhydrazone derivatives for selective t cell inhibitor and anti-lymphoid malignancy drug, March 6 2014. WO Patent App. PCT/KR2013/007,751.

[116] T.J. Choi and K.M. Youn. Organic electroluminescent compound and organic electroluminescent device using same, July 4 2013. WO Patent App. PCT/KR2012/011,340.

[117] Young Lok Choi, Bum Tae Kim, and Jung-Nyoung Heo. Total synthesis of laetevirenol a. *The Journal of Organic Chemistry*, 77(19):8762–8767, oct 2012.

[118] Y.M. Choi-Sledeski, G. Liang, and G.B. Poli. Spiropiperidine benzylamines as beta-tryptase inhibitors, June 30 2011. WO Patent App. PCT/US2010/061,464.

[119] Y.M. Choi-Sledeski, T.R. Nieduzak, G.B. Poli, P.W.K. Shum, G.T. Stoklosa, and Z. Zhao. [4[4-(5-aminomethyl-2-fluoro-phenyl)-piperidin-1-yl]-(1h-pyrrolo-pyridin-yl)-methanones and synthesis thereof, June 30 2011. WO Patent App. PCT/US2010/060,006.

[120] Pek Chong, Paul Sebahar, Michael Youngman, Dulce Garrido, Huichang Zhang, Eugene L Stewart, Robert T Nolte, Liping Wang, Robert G Ferris, Mark Edelstein, et al. Rational design of potent non-nucleoside inhibitors of hiv-1 reverse transcriptase. *Journal of medicinal chemistry*, 55(23):10601–10609, 2012.

[121] Tominari Choshi, Shiho Yamada, Eiichi Sugino, Takeshi Kuwada, and Satoshi Hibino. Total synthesis of grossularines-1 and -2. *The Journal of Organic Chemistry*, 60(18):5899–5904, sep 1995.

[122] J.M. Cid-Núñez, D. Oehlrlch, A.A. TRABANCO-Suarez, G.J. Tresadern, R.J.A. Vega, and G.J. Macdonald. 1,2,3-triazolo [4,3-a] pyridine derivatives and their use for the treatment or prevention of neurological and psychiatric disorders, November 18 2010. WO Patent App. PCT/EP2010/002,910.

[123] Martin Cigl, Alexej Bubnov, Miroslav Kašpar, František Hampl, Věra Hamplová, Oliva Pacherová, and Jiří Svoboda. Photosensitive chiral self-assembling materials: significant effects of small lateral substituents. *J. Mater. Chem. C*, 4(23):5326–5333, 2016.

[124] Adam J. Close, Paul Kemmitt, S. Mark Roe, and John Spencer. Regioselective routes to orthogonally-substituted aromatic MIDA boronates. *Org. Biomol. Chem.*, 14(28):6751–6756, 2016.

[125] V. Cmiljanovic, P. Hebeisen, F. Beaufils, T. Bohnacker, D. RAGEOT, A. SELE, M. Wymann, and J. LANGLOIS. Difluorométhyl-aminopyridines et difluorométhyl-aminopyrimidines, May 19 2016. WO Patent App. PCT/EP2015/076,192.

[126] A.E. Colvin, M.A. Mortellaro, and A. Modzelewska. Oxidation resistant indicator molecules, June 5 2008. WO Patent App. PCT/US2007/024,704.

[127] A.P. Combs, R.B. Sparks, T.P. Maduskuie, and J.D. Rodgers. Tricyclic heterocycles as bet protein inhibitors, September 18 2014. WO Patent App. PCT/US2014/027,872.

[128] P.J. Connolly, S.C. Lin, M.J. Macielag, and Y.M. Zhang. Pyrazines as delta opioid receptor modulators, May 5 2011. WO Patent App. PCT/US2010/054,469.

[129] P.J. Connolly, S.C. Lin, M.J. Macielag, and Y.M. Zhang. Pyrazines as delta opioid receptor modulators, May 5 2011. WO Patent App. PCT/US2010/054,469.

[130] Thomas C. Coombs, Cordelle Tanega, Min Shen, Jenna L. Wang, Douglas S. Auld, Samuel W. Gerritz, Frank J. Schoenen, Craig J. Thomas, and Jeffrey Aubé. Small-molecule pyrimidine inhibitors of the cdc2-like (clk) and dual specificity tyrosine phosphorylation-regulated (dyrk) kinases: Development of chemical probe ML315. *Bioorganic & Medicinal Chemistry Letters*, 23(12):3654–3661, jun 2013.

[131] J.W. Corbett, R.L. Elliott, K.D. Freeman-Cook, D.A. Griffith, and D.P. Phillion. Pyrazolospiroketone acetyl-c0a carboxylase inhibitors, December 3 2009. WO Patent App. PCT/IB2009/005,649.

[132] J.R. Corte, L.I. De, T. Fang, W. Yang, Y. Wang, A.K. Dilger, K.B. PABBISETTY, W.R. Ewing, Y. Zhu, R.R. Wexler, et al. Macrocycles with aromatic p2' groups as factor xia inhibitors, August 6 2015. WO Patent App. PCT/US2015/013,652.

[133] J.R. Corte, L.I. De, T. Fang, W. Yang, Y. Wang, A.K. Dilger, K.B. PABBISETTY, W.R. Ewing, Y. Zhu, R.R. Wexler, et al. Macrocycles with hetrocyclic p2' groups as factor xia inhibitors, August 6 2015. WO Patent App. PCT/US2015/013,654.

[134] Elias A. Couladouros, Emmanuel N. Pitsinos, Vassilios I. Moutsos, and Georgios Sarakinos. A general method for the synthesis of bastaranes and isobastaranes: First total synthesis of bastadins 5, 10, 12, 16, 20, and 21. *Chemistry - A European Journal*, 11(1):406–421, jan 2005.

[135] Mohane Selvaraj Coumar, Ming-Tsung Tsai, Chang-Ying Chu, Biing-Jiun Uang, Wen-Hsing Lin, Chun-Yu Chang, Teng-Yuan Chang, Jiun-Shyang Leou, Chi-Huang Teng, Jian-Sung Wu, Ming-Yu Fang, Chun-Hwa Chen, John T.-A. Hsu, Su-Ying Wu, Yu-Sheng Chao, and Hsing-Pang Hsieh. Identification, SAR studies, and x-ray co-crystallographic analysis of a novel furanopyrimidine aurora kinase a inhibitor. *ChemMedChem*, 5(2):255–267, feb 2010.

[136] J.J. Crawford, A.G. Dossetter, J.E. Finlayson, and N.M. Heron. Cyanocyclopropylcarboxamides as cathepsin inhibitors, December 31 2008. WO Patent App. PCT/GB2008/050,483.

[137] C.M.I. Crespo, Q.M. PRAT, R.S. Gual, P.L.J.C. Castro, and D.H. Slee. 2, 6 bisheteroaryl-4-aminopyrimidines as adenosine receptor antagonists, June 30 2005. WO Patent App. PCT/US2004/041,970.

[138] A.P. Crew, H. Dong, C. Ferraro, D. Sherman, and K.W. Siu. Macrocyclic kinase inhibitors, June 7 2012. WO Patent App. PCT/US2011/062,290.

[139] J.J. Cui, J.G. Deal, D. Gu, C. Guo, M.C. Johnson, R.S. Kania, S.E. Kephart, M.A. Linton, I.J. Mcalpine, M.A. Pairish, et al. Pyrazole compounds and their use as raf inhibitors, October 15 2009. WO Patent App. PCT/IB2008/001,952.

[140] M. Cullen, S. Hauck, B. Geng, M. FOLEY, C.M. Bastos, B. Munoz, and M. Haeberlein. Composés renforçant l'activité des protéasomes, May 21 2015. WO Patent App. PCT/US2014/065,204.

[141] Biswanath Das, Katta Venkateswarlu, Anjoy Majhi, Vidavalur Siddaiah, and Kongara Ravinder Reddy. A facile nuclear bromination of phenols and anilines using NBS in the presence of ammonium acetate as a catalyst. *Journal of Molecular Catalysis A: Chemical*, 267(1-2):30–33, apr 2007.

[142] Sanjib Das, Dnyaneshwar E. Shelke, Rajendra L. Harde, Vijayshree B. Avhad, Neelima Khairatkar-Joshi, Srinivas Gullapalli, Praveen K. Gupta, Maulik N. Gandhi, Deepak K. Bhateja, Malini Bajpai, Ashwini A. Joshi, Megha Y. Marathe, Girish S. Gudi, Satyawan B. Jadhav, Mahamad Yunnus A. Mahat, and Abraham Thomas. Design, synthesis and pharmacological evaluation of novel polycyclic heteroarene ethers as PDE10a inhibitors: Part II. *Bioorganic & Medicinal Chemistry Letters*, 24(15):3238–3242, aug 2014.

[143] Jennifer Davoren, Zhihan Wang, Yushi Chi, Anthony Harris, and David Gray. Scalable synthesis of 4-substituted 5-bromo-6-methylpyrimidines. *Synthesis*, 2011(10):1529–1531, mar 2011.

[144] M.A.P.A. De, J.G. Sterrenburg, H.C.A. Raaijmakers, A. Kaptein, A. Oubrie, J.B.M. Rewinkel, C.G.J.M. JANS, J.C.H.M. WIJKMANS, T.A. Barf, A.B. Cooper, et al. Btk inhibitors, January 24 2013. WO Patent App. PCT/CN2012/000,971.

[145] Alfonso de Dios, Chuan Shih, Beatriz López de Uralde, Concepción Sánchez, Miriam del Prado, Luisa M. Martín Cabrejas, Sehila Pleite, Jaime Blanco-Urgoiti, María José Lorite, C. Richard Nevill, Rosanne Bonjouklian, Jeremy York, Michal Vieth, Yong Wang, Nicholas Magnus, Robert M. Campbell, Bryan D. Anderson, Denis J. McCann, Deborah D. Giera, Paul A. Lee, Richard M. Schultz, Li, Lea M. Johnson, and Jeffrey A. Wolos. Design of potent and selective 2-aminobenzimidazole-based p38α MAP kinase inhibitors with excellent in vivo efficacy. *Journal of Medicinal Chemistry*, 48(7):2270–2273, apr 2005.

[146] Armin de Meijere, Ligang Zhao, Vladimir N. Belov, Mariano Bossi, Matthias Noltemeyer, and Stefan W. Hell. 1, 3-bicyclo[1.1.1]pentanediyl: The shortest rigid linear connector of phenylated photochromic units and a 1, 5-dimethoxy-9, 10-di(phenylethynyl)anthracene fluorophore. *Chemistry - A European Journal*, 13(9):2503–2516, mar 2007.

[147] Emmanuel Deau, Elodie Robin, Raluca Voinea, Nathalie Percina, Grzegorz Satała, Adriana-Luminita Finaru, Agnès Chartier, Gilles Tamagnan, David Alagille, Andrzej J. Bojarski, Séverine Morisset-Lopez, Franck Suzenet, and Gérald Guillaumet. Rational design, pharmacomodulation, and synthesis of dual 5-hydroxytryptamine 7 (5-HT7)/5-hydroxytryptamine 2a (5-HT2a) receptor antagonists and evaluation by [18f]-PET imaging in a primate brain. *Journal of Medicinal Chemistry*, 58(20):8066–8096, oct 2015.

[148] P. Deshpande, J. Grosso, A. Bhattacharya, and V. Purohit. Method of preparation of nitroaminopyridine compounds, February 8 2007. US Patent App. 11/492,730.

[149] H.D. Dietrich, J.D. Dittgen, M.W.D. Drewes, D.D. Feucht, H.D. Helmke, M. Hills, M.G.D. Hoffmann, H.D. Kehne, A.D. Martelletti, C.D. Rosinger, et al. New thiazole derivatives useful as herbicides and plant growth regulators, September 18 2008. DE Patent App. DE200,710,012,168.

[150] F.A. Dijcks, S.J. Lusher, H.T. Stock, and G.H. Veeneman. N-substituted azetidine derivatives, June 28 2012. WO Patent App. PCT/EP2011/073,041.

[151] T. Dineen, I.E. MARX, H.N. Nguyen, and M. Weiss. Heteroaryl sodium channel inhibitors, February 21 2013. WO Patent App. PCT/US2012/051,100.

[152] Nguyen Huu Dinh, LeVan Co, Nguyen Manh Tuan, Le Thi Hong Hai, and Luc Van Meervelt. New route to novel polysubstituted quinolines starting with eugenol, the main constituent ocimum sanctum l. oil. *HETEROCYCLES*, 85(3):627, 2012.

[153] J.A. Dixon, D. Nagarathnam, L. Zhang, C. Wang, L. Yi, Y. Chen, J. Chen, B. Bear, M. Brands, A. Hillisch, et al. 4-aminopyrimidine derivatives for treatment of hyperproliferative disorders, April 21 2005. WO Patent App. PCT/US2004/033,430.

[154] Robert Dodd, Kevin Cariou, Stephanie Gourdain, Jean Maurice Delabar, Nathalie Janel, Fernando Rodrigues Lima, Julien Dairou, and Clement Denhez. *Fr. Demande*, chapter Preparation of 3,5-diarylazaindoles as inhibitors of protein Dyrk1A for the treatment of cognitive deficiencies associated with Down syndrome and Alzheimer's disease., page 47pp. Number FR2999575A1. Centre National de la Recherche Scientifique CNRS , Fr.; Universite de Reims Champagne-Ardenne; Universite Paris Diderot Paris 7 ., Jun 2014. Copyright (C) 2017 American Chemical Society (ACS). All Rights Reserved.

[155] Timothy J. Donohoe, John F. Bower, José A. Basutto, Lisa P. Fishlock, Panayiotis A. Procopiou, and Cedric K.A. Callens. Ring-closing metathesis for the synthesis of heteroaromatics: evaluating routes to pyridines and pyridazines. *Tetrahedron*, 65(44):8969–8980, oct 2009.

[156] B.D. Dorsey, B.J. Dugan, K.M. Fowler, R.L. Hudkins, E.F. Mesaros, N.J. Monck, E.L. MORRIS, I. OLOWOYE, G.R. Ott, G.A. Pave, et al. Inhibiteurs azaquinazoline de la protéine kinase c atypique, October 1 2015. WO Patent App. PCT/US2015/022,368.

[157] Paul J. Dransfield, Vatee Pattaropong, Sujen Lai, Zice Fu, Todd J. Kohn, Xiaohui Du, Alan Cheng, Yumei Xiong, Renee Komorowski, Lixia Jin, Marion Conn, Eric Tien, Walter E. DeWolf, Ronald J. Hinklin, Thomas D. Aicher, Christopher F. Kraser, Steven A. Boyd, Walter C. Voegtli, Kevin R. Condroski, Murielle Veniant-Ellison, Julio C. Medina, Jonathan Houze, and Peter Coward. Novel series of potent glucokinase activators leading to the discovery of AM-2394. *ACS Medicinal Chemistry Letters*, 7(7):714–718, jul 2016.

[158] D.H. Drewry and R.N. Hunter. Chemical compounds, March 24 2005. WO Patent App. PCT/US2004/002,076.

[159] Jianxin Duan, Lian Hao Zhang, and Jr. William R. Dolbier. A convenient new method for the bromination of deactivated aromatic compounds. *Synlett*, 1999(8):1245–1246, aug 1999.

[160] M.P. Dwyer, T.J. Guzi, K. Paruch, R.J. Doll, K.M. Keertikar, and V.M. Girijavallabhan. Imidazopyridines as cyclin dependent kinase inhibitors, April 1 2004. WO Patent App. PCT/US2003/029,498.

[161] Paul Eastwood, Cristina Esteve, Jacob Gonza'lez, Silvia Fonquerna, Josep Aiguade', Ine's Carranco, Teresa Dome'nech, Mo'nica Aparici, Montserrat Miralpeix, Joan Alberti', Mo'nica Co'rdoba, Raquel Ferna'ndez, Merce' Pont, Nu'ria Godessart, Neus Prats, Mari'a Isabel Loza, Mari'a Isabel Cadavid, Arsenio Nueda, and Bernat Vidal. Discovery of LAS101057: A potent, selective, and orally efficacious a2badenosine receptor antagonist. *ACS Medicinal Chemistry Letters*, 2(3):213–218, mar 2011.

[162] P.R. Eastwood, R.J. Gonzalez, T.J. Bach, S.L.M. Pages, M.J. Taltavull, J.J.F. Caturla, and V.G. Matassa. Imidazopyridine derivatives as jak inhibitors, June 30 2011. WO Patent App. PCT/EP2010/007,913.

[163] F.H. Ebetino, A. Mazur, M.W. Lundy, and R.G.G. Russell. Imidazo[1,2-α]pyridinyl bisphosphonates, July 1 2010. WO Patent App. PCT/US2009/057,817.

[164] Kevin J Edgar and Stephen N Falling. An efficient and selective method for the preparation of iodophenols. *The Journal of Organic Chemistry*, 55(18):5287–5291, 1990.

[165] A. Edmunds, M. Muehlebach, A. Stoller, O. Loiseleur, A. Buchholz, O.F. Hueter, A. BIGOT, R.G. Hall, D. EMERY, P.J.M. Jung, et al. Pesticidally active bi- or tricyclic heterocycles with sulfur containing substituents, January 8 2015. WO Patent App. PCT/EP2014/062,946.

[166] D.D. Edney and A. Kennedy. Process for the preparation of pyrazine compounds, November 15 2001. WO Patent App. PCT/GB2001/001,929.

[167] M. El-Borai, A. H. Moustafa, M. Anwar, and F. I. Abdel Hay. *Polish Journal of Chemistry*, 55:1659, 1981.

[168] M El Borai, AJ MOUSTAFA, M Anwar, and FI Abdel Hay. Synthesis of halogen derivatives of n-methylimidazole. *Chemischer Informationsdienst*, 14(34), 1983.

[169] R. Elancheran, K. Saravanan, Bhaswati Choudhury, S. Divakar, S. Kabilan, M. Ramanathan, Babulal Das, R. Devi, and Jibon Kotoky. Design and development of oxobenzimidazoles as novel androgen receptor antagonists. *Medicinal Chemistry Research*, 25(4):539–552, jan 2016.

[170] M. M. El'chaninov, A. A. Aleksandrov, and E. V. Illenzeer. Synthesis and properties of 2-(2-furyl)[1,3]oxazolo[4,5-b]pyridine. *Russian Journal of Organic Chemistry*, 50(12):1826–1828, dec 2014.

[171] Fred Elustando, Mat Calder, and Cathy Lucas. *Brit. UK Pat. Appl.*, chapter Fused-ring heterocyclic compounds as BTK inhibitors, preparation and use in therapy., page 71pp. Number GB2515785A. Redx Pharma Limited, UK ., Jan 2015. Copyright (C) 2017 American Chemical Society (ACS). All Rights Reserved.

[172] Cécile Enguehard-Gueiffier, Simone Musiu, Nicolas Henry, Jean-Baptiste Véron, Sylvie Mavel, Johan Neyts, Pieter Leyssen, Jan Paeshuyse, and Alain Gueiffier. 3-biphenylimidazo[1,2-a]pyridines or [1,2-b]pyridazines and analogues, novel flaviviridae inhibitors. *European Journal of Medicinal Chemistry*, 64:448–463, jun 2013.

[173] Cristina Esteve, Jacob González, Sílvia Gual, Laura Vidal, Soledad Alzina, Sonia Sentellas, Irene Jover, Raquel Horrillo, Jorge De Alba, Montserrat Miralpeix, Gema Tarrasón, and Bernat Vidal. Discovery of 7-azaindole derivatives as potent orai inhibitors showing efficacy in a preclinical model of asthma. *Bioorganic & Medicinal Chemistry Letters*, 25(6):1217–1222, mar 2015.

[174] A. Estrada, W. Liu, S. PATEL, and M. Siu. Pyrazoles 3 substitués et utilisation en tant qu'inhibiteurs de dlk, July 24 2014. WO Patent App. PCT/EP2014/050,860.

[175] L.J. Farmer, M.J. Boyd, D.B. Miller, and V.A. CWYNAR. Methods of preparing inhibitors of influenza viruses replication, November 17 2016. WO Patent App. PCT/US2016/031,705.

[176] S.D. FIDANZE, D. Liu, R.A. Mantei, K.F. Mcdaniel, J. Pratt, G.S. Sheppard, L. Wang, A. Bogdan, J.H. Holms, J.D. DIETRICH, et al. Tetracyclic bromodomain inhibitors, September 18 2014. WO Patent App. PCT/CN2014/000,258.

[177] Luciano FORLANI, Alessandro MEDICI, Maurizio RICCI, and Paolo E. TODESCO. Reaction of 2-amino-5-bromo-1,3-thiazoles with sodium 2-hydroxyethoxide; preparation of 2-amino-3a,5,6,7a-tetrahydro[1,3]thiazolo[1,4]dioxins. *Synthesis*, 1977(05):320–322, 1977.

[178] Prisca Fricero, Laurent Bialy, Andrew W. Brown, Werngard Czechtizky, María Méndez, and Joseph P. A. Harrity. Synthesis and modular reactivity of pyrazole 5-trifluoroborates: Intermediates for the preparation of fully functionalized pyrazoles. *The Journal of Organic Chemistry*, 82(3):1688–1696, jan 2017.

[179] M.M. Friedman, T.D. Gordon, D.C. Ihle, S.G.Y. LO, M.J. Morytko, K.D. Mullen, L. Wang, K.P. Cusack, and A. Burchat. Heterocyclic nuclear hormone receptor modulators, March 13 2014. WO Patent App. PCT/US2013/058,397.

[180] Annette Frischmuth, Andreas Unsinn, Klaus Groll, Heinz Stadtmüller, and Paul Knochel. Preparations and reactions of SF5-substituted aryl and heteroaryl derivatives via mg and zn organometallics. *Chemistry - A European Journal*, 18(33):10234–10238, jun 2012.

[181] R.P. Frutos, D. Krishnamurthy, N.D. Patel, S. Rodriguez, and C.H. Senanayake. Process for making cytokine inhibiting compounds containing 4- and 5- imidazolyl rings and the intermediates thereof, October 25 2007. WO Patent App. PCT/US2007/066,684.

[182] A.M. Gelormini, A.W. Sledeski, and J.J. Shay. Use of 4-bromo-3-methyl-5-propoxythiophene-2-carboxylic acid 2,5-dioxo-pyrrolidin-1-yl ester for preparing the tryptase inhibitor [4-(5-aminomethyl-2-fluoro-phenyl)-piperidin-1-yl]-(4-bromo-3-methyl-5-propoxy-thiophen-2-yl)-methanone, October 9 2008. WO Patent App. PCT/US2008/058,341.

[183] Herman Gershon, Muriel Gershon, and Donald D. Clarke. Effect of n-halosucinimides on 5-, 7- and 5,7-8-quinolinol sulfonic acids. *Trends in Heterocyclic Chemistry*, 12:67 – 7, 2007.

[184] G. Giuliani, A. Cappelli, S. Vomero, and M. Anzini. Imidazo[1,2-α]pyrazin-3(7h)-one derivatives bearing a new electron-rich structure, January 20 2011. WO Patent App. PCT/IB2010/053,187.

[185] Asier Goitia, Enrique Gómez-Bengoa, and Arkaitz Correa. Selective c(sp2)–h halogenation of "click" 4-aryl-1,2,3-triazoles. *Organic Letters*, 19(4):962–965, feb 2017.

[186] Sonia Martínez González, Ana Isabel Hernández, Carmen Varela, Sonsoles Rodríguez-Arístegui, Milagros Lorenzo, Antonio Rodríguez, Virginia Rivero, José Ignacio Martín, Carl Gustav Saluste, Francisco Ramos-Lima, Elena Cendón, David Cebrián, Enara Aguirre, Elena Gomez-Casero, Maribel Albarrán, Patricia Alfonso, Beatriz García-Serelde, Julen Oyarzabal, Obdulia Rabal, Francisca Mulero, Teresa Gonzalez-Granda, Wolfgang Link, Jesús Fominaya, Mariano Barbacid, James R. Bischoff, Pilar Pizcueta, and Joaquín Pastor. Identification of ETP-46321, a potent and orally bioavailable PI3k α, δ inhibitor. *Bioorganic & Medicinal Chemistry Letters*, 22(10):3460–3466, may 2012.

[187] Vytautas. Grakauskas. Reactions of fluorodinitroethoxyacetyl chloride and fluorodinitroethoxyacetic anhydride with friedel-crafts catalysts. *The Journal of Organic Chemistry*, 41(11):2064–2065, may 1976.

[188] L. Green, E. Pinard, H. Ratni, and P. Williamson. Imidazo[1,2-a]pyrazin-1yl-benzamide compounds for treating spinal muscular atrophy, December 30 2015. WO Patent App. PCT/EP2015/063,894.

[189] A.D. Gribble, I.T. Forbes, A. Lightfoot, A.H. Payne, G. Walker, and V. Garzya. Composes, December 4 2003. WO Patent App. PCT/EP2003/005,727.

[190] K. Gudmundsson and B.A. Johns. Pyrazolo-pyridine derivatives as antiherpes agents, June 19 2003. WO Patent App. PCT/US2002/037,052.

[191] J.E.G. GUILLEMONT, M. PAUGAM, and B.F.M. DELEST. Hiv inhibiting 5-amido substituted pyrimidines, October 11 2007. WO Patent App. PCT/EP2007/053,111.

[192] V.R. Gummadi and S. Samajdar. Substituted indazole compounds as irak4 inhibitors, December 23 2015. WO Patent App. PCT/IB2015/054,620.

[193] Hamdy A. Hammouda, Ahmed A. El-Barbary, and Mohie A. F. Sharaf. Reactions with 5-aminopyrazoles. i. synthesis of halogen-containing fused pyrazoles. *Journal of Heterocyclic Chemistry*, 21(4):945–947, jul 1984.

[194] Chong Han, Keena Green, Eugen Pfeifer, and Francis Gosselin. Highly regioselective and practical synthesis of 5-bromo-4-chloro-3-nitro-7-azaindole. *Organic Process Research & Development*, 21(4):664–668, mar 2017.

[195] Fangbin Han, Songwen Lin, Peng Liu, Xiujie Liu, Jing Tao, Xiaobing Deng, Chongqin Yi, and Heng Xu. Discovery of a novel series of thienopyrimidine as highly potent and selective PI3k inhibitors. *ACS Medicinal Chemistry Letters*, 6(4):434–438, apr 2015.

[196] S.I. HAN, Y. Kim, and H.M. Kim. Organic light emitting compound and organic electroluminescent element using same, September 22 2016. WO Patent App. PCT/KR2015/014,035.

[197] E. Smakula Hand and William W. Paudler. Teleamination of the imidazo[1,2-a]pyridine system. *The Journal of Organic Chemistry*, 43(14):2900–2906, jul 1978.

[198] Jason J. Hanthorn, Luca Valgimigli, and Derek A. Pratt. Preparation of highly reactive pyridine- and pyrimidine-containing diarylamine antioxidants. *The Journal of Organic Chemistry*, 77(16):6908–6916, aug 2012.

[199] M.J. HARGREAVES, M. LEVINE, J.Z. MOR, A. Ngo, T.A. REEVES, G.L. RHYS-JONES, M.A. TARRANT, and M.E. WILDENAUER. Electronic data generation methods, January 17 2013. US Patent App. 13/524,118.

[200] Anthony R. Harris, Deane M. Nason, Elizabeth M. Collantes, Wenjian Xu, Yushi Chi, Zhihan Wang, Bingzhi Zhang, Qingjian Zhang, David L. Gray, and Jennifer E. Davoren. Synthesis of 5-bromo-6-methyl imidazopyrazine, 5-bromo and 5-chloro-6-methyl imidazopyridine using electron density surface maps to guide synthetic strategy. *Tetrahedron*, 67(47):9063–9066, nov 2011.

[201] T. Harrison, C. O'dowd, S. SHEPHERD, G. TREVITT, L. Zhang, and F. Burkamp. 6-(4- (1 -amino- 3 -hydroxycyclobutyl) phenyl) - 5 - phenyl (furo, thieno or pyrrolo) [2, 3-d] pyrimidin- 4 - one derivatives for the treatment of cancer, September 26 2013. WO Patent App. PCT/GB2013/050,771.

[202] Lisa A. Hasvold, George S. Sheppard, Le Wang, Steven D. Fidanze, Dachun Liu, John K. Pratt, Robert A. Mantei, Carol K. Wada, Robbert Hubbard, Yu Shen, Xiaoyu Lin, Xiaoli Huang, Scott E. Warder, Denise Wilcox, Leiming Li, F. Greg Buchanan, Lauren Smithee, Daniel H. Albert, Terrance J. Magoc, Chang H. Park, Andrew M. Petros, Sanjay C. Panchal, Chaohong Sun, Peter Kovar, Nirupama B. Soni, Steven W. Elmore, Warren M. Kati, and Keith F. McDaniel. Methylpyrrole inhibitors of BET bromodomains. *Bioorganic & Medicinal Chemistry Letters*, 27(10):2225–2233, may 2017.

[203] K. Hayashi, T. Watanabe, K. Toyama, J. Kamon, M. Minami, M. UNI, and M. Nasu. Composés hétérocycliques tricycliques et inhibiteurs de jak, February 21 2013. WO Patent App. PCT/JP2012/070,876.

[204] Damien Hédou, Rémi Guillon, Charlotte Lecointe, Cédric Logé, Elizabeth Chosson, and Thierry Besson. Novel synthesis of angular thiazolo[5,4-f] and [4,5-h]quinazolines, preparation of their linear thiazolo[4,5-g] and [5,4-g]quinazoline analogs. *Tetrahedron*, 69(15):3182–3191, apr 2013.

[205] M. Heffernan, J. Dorsey, Q. Fang, R. Foglesong, S. Hopkins, M. Jones, S. Jones, C. Ogbu, J. Perales, M. Soukri, et al. Fused heterocyclic inhibitors of d-amino acid oxidase, March 6 2008. US Patent App. 11/825,093.

[206] John T. Henssler and Adam J. Matzger. Facile and scalable synthesis of the fused-ring heterocycles thieno[3,2-b]thiophene and thieno[3,2-b]furan. *Organic Letters*, 11(14):3144–3147, jul 2009.

[207] W.J. Hoekstra, S.W. Rafferty, C.M. YATES, R.J. Schotzinger, M. Loso, and M. Sullenberger. Metalloenzyme inhibitor compounds, December 27 2012. WO Patent App. PCT/US2012/043,094.

[208] S. Hoelder, J. Blagg, S. SOLANKI, H. Woodward, S. NAUD, V. Bavetsias, P. SHELDRAKE, P. INNOCENTI, J. CHEUNG, and B. Atrash. Inhibitor compounds, March 13 2014. WO Patent App. PCT/GB2013/052,360.

[209] K.R. Hornberger, D.M. Berger, X. Chen, A.P. Crew, H. Dong, A. Kleinberg, A.H. Li, L. Ma, M.J. Mulvihill, B. Panicker, et al. 7-aminofuropyridine derivatives, September 22 2011. WO Patent App. PCT/US2011/024,449.

[210] Takamitsu Hosoya, Rie Iimori, Suguru Yoshida, Yuto Sumida, Yuiko Sahara-Miura, Jun ichi Sato, and Satoshi Inouye. Concise synthesis ofv-coelenterazines. *Organic Letters*, 17(15):3888–3891, aug 2015.

[211] D. Hung, M. Serrano-Wu, S. GRANT, and T. Kawate. Substituted aminothiazoles for the treatment of tuberculosis, October 2 2014. WO Patent App. PCT/US2014/025,488.

[212] Munawar Hussain, Nguyen Thai Hung, and Peter Langer. Efficient synthesis of functionalized dibenzofurans by domino 'twofold heck/6π-electrocyclization' reactions of 2,3-di- and 2,3,5-tribromobenzofuran. *Tetrahedron Letters*, 50(27):3929–3932, jul 2009.

[213] G. Hynd, P. Tisselli, J.J. Kulagowski, C. Macleod, S.E. MANN, S.C. PRICE, and J.G. Montana. New compounds as nik inhibitors, April 28 2016. WO Patent App. PCT/EP2015/074,437.

[214] Ken ichi Kusakabe, Nobuyuki Ide, Yataro Daigo, Takeshi Itoh, Takahiko Yamamoto, Hiroshi Hashizume, Kohei Nozu, Hiroshi Yoshida, Genta Tadano, Sachie Tagashira, Kenichi Higashino, Yousuke Okano, Yuji Sato, Makiko Inoue, Motofumi Iguchi, Takayuki Kanazawa, Yukichi Ishioka, Keiji Dohi, Yasuto Kido, Shingo Sakamoto, Shigeru Ando, Masahiro Maeda, Masayo Higaki, Yoshiyasu Baba, and Yusuke Nakamura. Discovery of imidazo[1, 2-b]pyridazine derivatives: Selective and orally available mps1 (TTK) kinase inhibitors exhibiting remarkable antiproliferative activity. *Journal of Medicinal Chemistry*, 58(4):1760–1775, feb 2015.

[215] Takashi Ikawa, Shigeaki Masuda, Akira Takagi, and Shuji Akai. 1,3- and 1,4-benzdiyne equivalents for regioselective synthesis of polycyclic heterocycles. *Chem. Sci.*, 7(8):5206–5211, 2016.

[216] C. Illig, S. Ballentine, J. Chen, S. Meegalla, M. Rudolph, M. Wall, K. Wilson, R. Desjarlais, C. Manthey, C. Flores, et al. Inhibitors of c-fms kinase, August 24 2006. US Patent App. 11/408,096.

[217] C.R. Illig, S.K. Ballentine, J. Chen, R.L. DesJarlais, S.K. Meegalla, M. Wall, and K. Wilson. Inhibitors of c-fms kinase, October 25 2007. US Patent App. 11/736,617.

[218] S. Inouye, Y. Sahara, and T. Hosoya. Coelenterazine analogues and coelenteramide analogues, October 6 2011. US Patent App. 13/080,385.

[219] J. Iso-Sipilä, H. LEE, J. BALEY, and J. Osborne. System and method for inputting text into electronic devices, June 18 2015. WO Patent App. PCT/GB2014/053,665.

[220] E.J. Jacobsen, J.R. BLINN, J.R. Springer, and S.K. HOCKERMAN. Heterocyclic itk inhibitors for treating inflammation and cancer, July 28 2016. WO Patent App. PCT/US2016/014,729.

[221] G. Jaeschke, S. Kolczewski, R. Porter, P. Schnider, and E. Vieira. Naphthyridin derivatives, April 5 2007. US Patent App. 11/529,992.

[222] G. Jaeschke, L. Lindemann, E. Vieira, and J. Wichmann. Imidazole derivatives as mglur5 antagonists, January 20 2011. WO Patent App. PCT/EP2010/060,097.

[223] Josef Jansa, Antonín Lyčka, Aleš Růžička, Martin Grepl, and Jan Vaněček. Synthesis, structure and rearrangement of iodinated imidazo[1,2-c]pyrimidine-5(6h)-ones derived from cytosine. *Tetrahedron*, 71(1):27–36, jan 2015.

[224] J.B. Jaquith, G. Villeneuve, A. Boudreault, S. Morris, J. Durkin, J.W. Gillard, K. Hewitt, and H.N. Marsh. Imidazo [2,1-b]-1,3,4-thiadiazole suflonamides, June 26 2003. WO Patent App. PCT/CA2002/001,942.

[225] Henning Jacob Jessen, Andreas Schumacher, Travis Shaw, Andreas Pfaltz, and Karl Gademann. A unified approach for the stereoselective total synthesis of pyridone alkaloids and their neuritogenic activity. *Angewandte Chemie International Edition*, 50(18):4222–4226, mar 2011.

[226] J. Ji, T. Li, and Y. Wang. 3-quinuclidinyl amino-substituted biaryl derivatives, July 21 2005. US Patent App. 11/015,158.

[227] Pan-Pan Jiang and Xian-Jin Yang. A quick, mild and efficient bromination using a CFBSA/KBr system. *RSC Adv.*, 6(93):90031–90034, 2016.

[228] Pan-Pan Jiang and Xian-Jin Yang. A quick, mild and efficient bromination using a CFBSA/KBr system. *RSC Adv.*, 6(93):90031–90034, 2016.

[229] J.M. Jimenez and P. Collier. Pyrrolo(3,2-c) pyridines useful as inhibitors of protein kinases, August 23 2007. WO Patent App. PCT/US2007/003,767.

[230] Adrien Joliton, Jean-Marc Plancher, and Erick M. Carreira. Formation of α-SF5 -enolate enables preparation of 3-SF5 -quinolin-2-ones, 3-SF5 -quinolines, and 3-SF5 -pyridin-2-ones: Evaluation of their physicochemical properties. *Angewandte Chemie International Edition*, 55(6):2113–2117, jan 2016.

[231] C. Jones and Z.S. Matusiak. Chemical compounds, December 27 2007. WO Patent App. PCT/GB2007/002,279.

[232] Darin E. Jones and Michael S. South. Synthesis of a versatile 2 (1h)-pyrazinone core for the preparation of tissue factor-factor VIIa inhibitors. *Tetrahedron*, 66(14):2570–2581, apr 2010.

[233] P. Jones, D.C. Pryde, and T.D. Tran. 3 -deazapurine derivatives as tlr7 modulators, August 23 2007. WO Patent App. PCT/IB2007/000,368.

[234] R.M. Jones, G. Semple, B. Fioravanti, G. Pereira, I. Calderon, J. Uy, K. Duvvuri, J.S.K. Choi, Y. Xiong, and V. Dave. 1,2,3-trisubstituted aryl and heteroaryl derivatives as modulators of metabolism and the prpphylaxis and treatment of disorders related thereto such as diabetes and hyperglycemia, October 21 2004. WO Patent App. PCT/US2004/001,267.

[235] Charlotte G. Jørgensen, Rasmus P. Clausen, Kasper B. Hansen, Hans Bräuner-Osborne, Birgitte Nielsen, Bjørn Metzler Bjørn Metzler, Jan Kehler, Povl Krogsgaard-Larsen, and Ulf Madsen. Synthesis and pharmacology of glutamate receptor ligands: new isothiazole analogues of ibotenic acid. *Org. Biomol. Chem.*, 5(3):463–471, 2007.

[236] Janusz Jurczak, Ryszard Ostaszewski, Giovanni D. Andreetti, and Gianluca Calestani. The synthesis and structure of chiral di-n-p-toluenesulphonyl diazacoronands derived from l-tartaric acid. *HETEROCYCLES*, 31(3):397, 1990.

[237] Thomas K" ohler, Jürgen Faderl, Hans Pritzkow, and Walter Siebert. Synthesis and properties of bis(2-heteroaryl)borane derivatives. *European Journal of Inorganic Chemistry*, 2002(11):2942–2946, nov 2002.

[238] Youssef Kabri, Maxime D. Crozet, Thierry Terme, and Patrice Vanelle. Efficient access to 2,6,8-trisubstituted 4-aminoquinazolines through microwave-assisted one-pot chemoselective tris-suzuki-miyaura or SNAr/bis-suzuki-miyaura reactions in water. *European Journal of Organic Chemistry*, 2015(17):3806–3817, may 2015.

[239] V.J. Kalish, F.A. Brookfield, S.M. Courtney, and L.M. Frost. N-hydroxymethanesulfonamide nitroxyl donors, July 23 2015. WO Patent App. PCT/US2015/011,792.

[240] Dipannita Kalyani, Allison R. Dick, Waseem Q. Anani, and Melanie S. Sanford. Scope and selectivity in palladium-catalyzed directed c–h bond halogenation reactions. *Tetrahedron*, 62(49):11483–11498, dec 2006.

[241] Toshiyuki Kamei, Aoi Ishibashi, and Toyoshi Shimada. Metal-free halogenation of arylboronate with n-halosuccinimide. *Tetrahedron Letters*, 55(30):4245–4247, jul 2014.

[242] Kiyoshi Kanie, Katsuya Mizuno, Manabu Kuroboshi, and Tamejiro Hiyama. A facile synthesis of trifluoromethylamines by oxidative desulfurization–fluorination of dithiocarbamates. *Bulletin of the Chemical Society of Japan*, 71(8):1973–1991, aug 1998.

[243] Takanori Kawaguchi, Kengo Watatani, Keiko Fushigi, Masahiro Bohno, Hajime Asanuma, Shoichi Kuroda, Yudai Imai, Tomomichi Chonan, Nagaaki Sato, Shigeru Tokita, Shigemasa Sasako, Takumi Okada, Keiji Hayashi, Susumu Ito, Noriko Saito, Rui Jibiki, Kiyoshi Ishiyama, and Hiroshi Ota. *Jpn. Kokai Tokkyo Koho*, chapter Preparation of 2-pyridone compounds as glucokinase activators., page 417pp. Number JP2013010750A. Taisho Pharmaceutical Co., Ltd., Japan; Nissan Chemical Industries, Ltd. ., Jan 2013. Copyright (C) 2017 American Chemical Society (ACS). All Rights Reserved.

[244] S. Kawata, K. Matsumoto, Sachi Matsumoto Seoul, M. Niijima, Niijima Ma Gui, and T. achi Takahashi Takahashi. Novel thyroid hormone β receptor agonist, February 17 2011. WO Patent App. PCT/JP2010/056,936.

[245] M. Kelly, Y. Lee, B. Liu, T. Fujimoto, J. Freundlich, B.D. Dorsey, G.A. Flynn, A. Husain, and W.R. Moore. Anti-cancer agents and uses thereof, November 13 2008. US Patent App. 11/819,494.

[246] C.P. Kenyon, L.C. Oldfield, D.W.C.W. Van, A.L. Rousseau, and C.J. Parkinson. Modulation of phosphoryl transferase activity of glutamine synthetase, September 20 2007. WO Patent App. PCT/IB2006/000,565.

[247] Brian D. Kesling, Bjørn C. Søderberg, and Terry Gullion. Synthesis of isotopically labeled tri-p-tolylamine. *Journal of Labelled Compounds and Radiopharmaceuticals*, 43(10):1059–1068, 2000.

[248] Jason G. Kettle, Peter Ballard, Catherine Bardelle, Mark Cockerill, Nicola Colclough, Susan E. Critchlow, Judit Debreczeni, Gary Fairley, Shaun Fillery, Mark A. Graham, Louise Goodwin, Sylvie Guichard, Kevin Hudson, Richard A. Ward, and David Whittaker. Discovery and optimization of a novel series of dyrk1b kinase inhibitors to explore a MEK resistance hypothesis. *Journal of Medicinal Chemistry*, 58(6):2834–2844, mar 2015.

[249] Gyo Chang Keum, Eun Gyeong Kim, Seon Hui Seo, Jae Uk Lee, Ju Hyeon Lee, and Sang Cheol Shin. *Repub. Korean Kongkae Taeho Kongbo*, chapter Preparation of pyrrolopyrimidine derivatives as anticancer agents., page 39pp. Number KR2016059366A. Korea Institute of Science and Technology, S. Korea ., May 2016. Copyright (C) 2017 American Chemical Society (ACS). All Rights Reserved.

[250] Abdellatif El Kihel, Mohamed Benchidmi, El Mokhtar Essassi, and Renée Danion-Bougot. Halogenation of substituted benzimidazoles. nitration of the resulting halobenzimidazoles. *Synthetic Communications*, 29(3):387–397, feb 1999.

[251] H.J. Kim, M.Y. Chae, W. Kim, N.H. Lee, D.H. Huh, et al. Compound for organic optoelectronic device, organic light-emitting diode comprising same, and display device comprising organic light emitting diode, December 11 2014. WO Patent App. PCT/KR2013/006,588.

[252] P. KIM, H.R. Kim, S.Y. Cho, J. Du Ha, H.J. Jung, et al. N2-(2-methoxyphenyl)pyrimidine derivative, method for preparing same, and pharmaceutical composition for cancer prevention or treatment containing same as active ingredient, December 15 2016. WO Patent App. PCT/KR2016/003,597.

[253] S.H. Kim, G.N. Anilkumar, M.K.C. Wong, Q. Zeng, S.B. Rosenblum, J.A. Kozlowski, Y. Shao, G.B.F. Mc, and D.W. Hobbs. Heterocyclic substituted piperazines with cxcr3 antagonist activity, August 24 2006. WO Patent App. PCT/US2006/005,123.

[254] Yong Ju Kim, Tae Gyo Park, Seong Ho Woo, Hyang Suk Lee, Seon Yeong Kim, Jong Un Cho, Hong Beom Lee, Yeong Un Kim, and Du Hwan Jung. *Repub. Korean Kongkae Taeho Kongbo*, chapter Preparation of imidazole derivatives as HSP90 inhibitors., page 110pp. Number KR2012109667A. Regochem Bioscience Co., Ltd., S. Korea ., Oct 2012. Copyright (C) 2017 American Chemical Society (ACS). All Rights Reserved.

[255] T. Kimura, N. Ohkawa, A. Nakao, T. Nagasaki, and T. Shimozato. Cyclic tertiary amine compound, March 1 2007. US Patent App. 11/301,296.

[256] Frank D. King, Abil E. Aliev, Stephen Caddick, and D. A. Tocher. A novel synthesis of (di)-benzazocinones via an endocyclic n-acyliminium ion cyclisation. *Organic & Biomolecular Chemistry*, 9(5):1547, 2011.

[257] Evgueni Kirillov, Thierry Roisnel, Abbas Razavi, and Jean-Francçis Carpentier. Chromium(III) complexes of sterically crowded bidentante {ONR} and tridentate {ONNR} naphthoxy-imine ligands: Syntheses, structures, and use in ethylene oligomerization‡. *Organometallics*, 28(8):2401–2409, apr 2009.

[258] U. Klar, M. Koppitz, D. Kosemund, R. Bohlmann, B. Bader, P. Lienau, G. Siemeister, S. Prechtl, D. Nguyen, and W. Scott. Imidazopyrazines, September 22 2011. WO Patent App. PCT/EP2011/053,967.

[259] H.P. Klein, R.A. Grigsby, J. Zhou, P. Holub, A. Gaspar, and Z. Gaspar. Use of 2-(3-aminopropoxy)ethan-1-ol as an absorbent to remove acidic gazes, May 3 2012. WO Patent App. PCT/US2011/056,610.

[260] Y. Kobayashi, H. Daido, H. Katsuta, M. Nomura, H. Tsukada, A. Hirabayashi, Y. Takahashi, Y. Aoki, A. Kawahara, Y. FUKAZAWA, et al. Amide derivative, pest control agent containing the amide derivative, and use of the amide derivative, August 18 2011. US Patent App. 13/016,604.

[261] J.T. KOBERTSTEIN, S. SAMANTA, and S.C. DE. Biodegradable thermo-responsive polymers and uses thereof, June 9 2016. WO Patent App. PCT/US2015/063,669.

[262] Judit Kosáry and Zoltán Gabányi. Über den pyridazinring enthaltende verbindungen, 16. elektrophile substitution des [1, 2, 4] triazolo [4, 3-b] pyridazins. *Chemische Berichte*, 116(10):3513–3515, 1983.

[263] Judit Kosáry and Zoltán Gabányi. Über den pyridazinring enthaltende verbindungen, 16. elektrophile substitution des [1,2,4]triazolo[4,3-b]pyridazins. *Chemische Berichte*, 116(10):3513–3515, oct 1983.

[264] Vasiliki Kotzabasaki, Georgios Vassilikogiannakis, and Manolis Stratakis. Regiocontrolled synthesis of γ-hydroxybutenolides via singlet oxygen-mediated oxidation of 2-thiophenyl furans. *The Journal of Organic Chemistry*, 81(10):4406–4411, may 2016.

[265] G. D. Krapivin, N. I. Val'ter, V. E. Zavodnik, T. Ya. Kaklyugina, and V. G. Kul'nevich. 5-[5-r-furfurylidene]-2,2-dimethyl-1,3-dioxane-4,6-diones 6. synthesis, structure, and properties of substituted furylimino- and furfurylidenephosphoranes. molecular and crystal structure of n-[5-(2,2-dimethyl-4,6-dioxo-1,3-dioxan-5-ylidene)methyl-2-furylimino(triphenyl)phosphorane. *Chemistry of Heterocyclic Compounds*, 30(3):296–304, mar 1994.

[266] T. Kress and S. M. Costantino. *J. Heterocycl. Chem.*, 10(3):409–410, 1973.

[267] A. Kruczynski, L. Creancier, E.B. Kaloun, K. Bedjeguelal, and R. RABOT. Derivatives of azaindazole or diazaindazole type for treating a cancer overexpressing trk, January 30 2014. WO Patent App. PCT/EP2013/065,913.

[268] K. Kusakabe, H. Yoshida, K. Nozu, H. Hashizume, G. Tadano, J. Sato, Y. Tamura, and Y. Mitsuoka. Fused imidazole derivative having ttk inhibitory action, March 8 2012. US Patent App. 13/192,369.

[269] Clemens Lamberth, Raphael Dumeunier, Stephan Trah, Sebastian Wendeborn, Jeremy Godwin, Peter Schneiter, and Andy Corran. Synthesis and fungicidal activity of tubulin polymerisation promoters. part 3: Imidazoles. *Bioorganic & Medicinal Chemistry*, 21(1):127–134, jan 2013.

[270] Clemens Lamberth, Raphael Dumeunier, Stephan Trah, Sebastian Wendeborn, Jeremy Godwin, Peter Schneiter, and Andy Corran. Synthesis and fungicidal activity of tubulin polymerisation promoters. part 3: Imidazoles. *Bioorganic & Medicinal Chemistry*, 21(1):127–134, jan 2013.

[271] Tlabo C. Leboho, Sandy F. van Vuuren, Joseph P. Michael, and Charles B. de Koning. The acid-catalysed synthesis of 7-azaindoles from 3-alkynyl-2-aminopyridines and their antimicrobial activity. *Org. Biomol. Chem.*, 12(2):307–315, 2014.

[272] L.C.L. LECLERCQ, J.M. BARTOLOMÉ-NEBREDA, S. Conde-Ceide, and G.M.L.M. Van. Inhibitors of phosphodiesterase 10 enzyme, January 16 2014. WO Patent App. PCT/EP2013/064,355.

[273] Wendy Lee, James J. Crawford, Ignacio Aliagas, Lesley J. Murray, Suzanne Tay, Weiru Wang, Christopher E. Heise, Klaus P. Hoeflich, Hank La, Simon Mathieu, Robert Mintzer, Sreemathy Ramaswamy, Lionel Rouge, and Joachim Rudolph. Synthesis and evaluation of a series of 4-azaindole-containing p21-activated kinase-1 inhibitors. *Bioorganic & Medicinal Chemistry Letters*, 26(15):3518–3524, aug 2016.

[274] A.H. Li, A.G. Steinig, A. Kleinberg, Q. Weng, M.J. Mulvihill, J. Wang, X. Chen, T. Wang, H. Dong, and M. Jin. Furo- and thieno [3,2-c] pyridines, August 6 2009. US Patent App. 12/366,743.

[275] G. Li, Q. Li, T. Li, N.H. Lin, R. Mantei, H. Sham, and G. Wang. Urea kinase inhibitors, February 19 2004. US Patent App. 10/218,121.

[276] Guo Li, Ramesh Kakarla, and Samuel W. Gerritz. A fast and efficient bromination of isoxazoles and pyrazoles by microwave irradiation. *Tetrahedron Letters*, 48(26):4595–4599, jun 2007.

[277] Hui-Jing Li, Yan-Chao Wu, Jian-Hong Dai, Yan Song, Runjiao Cheng, and Yuanyuan Qiao. Regioselective electrophilic aromatic bromination: Theoretical analysis and experimental verification. *Molecules*, 19(3):3401–3416, mar 2014.

[278] Lu Li, Dachuan Qiu, Jiarong Shi, and Yang Li. Vicinal diamination of arenes with domino aryne precursors. *Organic Letters*, 18(15):3726–3729, aug 2016.

[279] Jürgen Liebscher and Lunxiang Yin. Convenient synthesis of substituted 3-alkenylpyrazolo[1,5-a]pyrimidines via heck cross-coupling reaction. *Synthesis*, 2004(14):2329–2334, 2004.

[280] J. Lin, P. Pham, J. BUELL, K. Dong, P. Ibrahim, W. Spevak, G. TSANG, G. Wu, and Y. Zhang. Compounds and methods for kinase modulation, and indications therefor, October 13 2016. WO Patent App. PCT/US2016/026,523.

[281] Yuan-bin Lin, Yi-sha Deng, Hong-biao Chen, Jian-xiang Yin, Chun-ming Li, and Ru-lai Liu. A study on bromination of deactivated aromatics. *Xiangtan Daxue Ziran Kexue Xuebao*, 30(4):85–88, 2008. Copyright (C) 2017 American Chemical Society (ACS). All Rights Reserved.

[282] D. L. Lipilin, A. M. Churakov, Yu. A. Strelenko, and V. A. Tartakovsky. *Russian Chemical Bulletin*, 51(2):311–318, 2002.

[283] Z. Liu, N. Hundal-Jabal, M. Wong, D. Yapp, K.-S. Lin, F. Bénard, and D. M. Perrin. A new18f-heteroaryltrifluoroborate radio-prosthetic with greatly enhanced stability that is labelled by18f–19f-isotope exchange in good yield at high specific activity. *Med. Chem. Commun.*, 5(2):171–179, 2014.

[284] Zhong Liu. Preparation of imidazo-pyridine compounds in preparation of pi3k inhibitors, March 29 2017. CN Patent 105,130,984.

[285] R. Lochtman, M. Keil, J. Gebhardt, M. Rack, and D.W. Von. Method for producing 4-bromine-aniline derivatives, January 10 2002. WO Patent App. PCT/EP2001/007,482.

[286] O. Loiseleur, P. Durieux, S. Trah, A. Edmunds, A. Jeanguenat, A. Stoller, and D.J. Hughes. Pesticides containing a bicyclic bisamide structure, August 23 2007. WO Patent App. PCT/EP2007/001,283.

[287] R. Lopez, S. Hebbe, D. Gillet, and J. Barbier. New compounds with activity that protects against the action of toxins and viruses in intracellular action mode, January 20 2010. EP Patent App. EP20,080,290,570.

[288] Rosario López-Moreno, Josep V. Mercader, Consuelo Agulló, Antonio Abad-Somovilla, and Antonio Abad-Fuentes. Immunoassays for trifloxystrobin analysis. part i. rational design of regioisomeric haptens and production of monoclonal antibodies. *Food Chemistry*, 152:230–236, jun 2014.

[289] E. Lukevics, L. Ignatovich, Yu. Coldberg, F. Polyak, A. Gaukhman, S. Rozite, and J. Popelis. Reactions of trialkyl(2-furyl) germanes with electrophilic reagents. *Journal of Organometallic Chemistry*, 348(1):11–23, jun 1988.

[290] Jun Lv, Qiancai Liu, Jie Tang, Franc Perdih, and Kristof Kranjc. A facile synthesis of indolo[3,2,1-jk]carbazoles via palladium-catalyzed intramolecular cyclization. *Tetrahedron Letters*, 53(39):5248–5252, sep 2012.

[291] M.J. Macielag and J.J. McNally. Sulfonamides as trpm8 modulators, June 24 2010. US Patent App. 12/638,550.

[292] A. Macleod, D.R. Mitchell, N.J. Palmer, C.C. Parsy, M.D. Goldsmith, and C.J. Harris. Imidazo [1,2-a] pyrazine compounds for treatment of viral infections such as hepatitis, February 26 2009. WO Patent App. PCT/EP2008/060,896.

[293] Matthew Maddess and Rhiannon Carter. SNAr reactions of 2-methylthio-4-pyrimidinones in pivalic acid: Access to functionalized pyrimidinones and pyrimidines. *Synthesis*, 44(07):1109–1118, mar 2012.

[294] Sean M. Maddox, Christopher J. Nalbandian, Davis E. Smith, and Jeffrey L. Gustafson. A practical lewis base catalyzed electrophilic chlorination of arenes and heterocycles. *Organic Letters*, 17(4):1042–1045, feb 2015.

[295] Zahia Mahiout, Thierry Lomberget, Sylvie Goncalves, and Roland Barret. Solvent-dependent oxidations of 5- and 6-azaindoles to trioxopyrrolopyridines and functionalised azaindoles. *Organic & Biomolecular Chemistry*, 6(8):1364, 2008.

[296] D.A.P.A. Man, J.B.M. Rewinkel, C.G.J.M. Jans, H.C.A. Raaijmakers, and J.C.H.M. Wijkmans. 8-methyl-1-phenyl-imidazol[1,5-a]pyrazine compounds, August 11 2011. WO Patent App. PCT/EP2011/051,584.

[297] Pascal Marchand, Maud Antoine, Matthias Gerlach, Eckhard Günther, Tilmann Schuster, Michael Czech, and Irene Seipelt. A convenient synthesis of novel 2, 8-disubstituted pyrido[3, 4-b]pyrazines possessing biological activity. *Synthesis*, 44(01):69–82, nov 2011.

[298] Jesse A. May, Najam A. Sharif, Marsha A. McLaughlin, Hwang-Hsing Chen, Bryon S. Severns, Curtis R. Kelly, William F. Holt, Richard Young, Richard A. Glennon, Mark R. Hellberg, and Thomas R. Dean. Ocular hypotensive response in nonhuman primates of (8r)-1-[(2s)-2-aminopropyl]-8,9-dihydro-7h-pyrano[2,3-g]indazol-8-ol a selective 5-HT2receptor agonist. *Journal of Medicinal Chemistry*, 58(22):8818–8833, nov 2015.

[299] Dennis J. McNamara, P. Dan Cook, Lois B. Allen, Mary Jo Kehoe, Carolyn S. Holland, and Annette G. Teepe. Synthesis, antitumor activity, and antiviral activity of 3-substituted 3-deazacytidines and 3-substituted 3-deazauridines. *Journal of Medicinal Chemistry*, 33(7):2006–2011, jul 1990.

[300] E. B. Melnikova, M. M. Elchaninov, A. A. Milov, and B. S. Lukyanov. Synthesis and properties of 2-(2-furyl)benzothiazole. *Chemistry of Heterocyclic Compounds*, 44(9):1070–1076, sep 2008.

[301] Laura C. Meurer, Richard L. Tolman, Edward W. Chapin, Richard Saperstein, Pasquale P. Vicario, Matthew M. Zrada, and Malcolm MacCoss. Synthesis and hypoglycemic activity of substituted 8-(1-piperazinyl)imidazo[1,2-a]pyrazines. *Journal of Medicinal Chemistry*, 35(21):3845–3857, oct 1992.

[302] M. Michels, M. Follmann, A. Vakalopoulos, K. Zimmermann, N. Teusch, and K. Engel. Substituted 4- (indazolyl) -1,4-dihydropyridines and methods of use thereof, December 17 2009. WO Patent App. PCT/EP2009/003,838.

[303] Iulia Mihai, Maria Ivanoiu, Loredana Vacareanu (Stafie), and Mircea Grigoras. Suzuki polycondensation: a simple way for synthesis of new polyazines containing thiophene and fluorene moieties. *e-Polymers*, 11(1), jan 2011.

[304] J. Mihara, K. Araki, T. Mori, T. Murata, Y. Yoneta, E. Shimojo, T. Ichihara, M. Ataka, K. Shibuya, and U. Görgens. Pesticidal carboxamides, May 5 2011. WO Patent App. PCT/EP2010/004,217.

[305] M. Miller, K. Basu, D. Demong, J. Scott, W. Li, A. Stamford, M. Poirier, and P. Tempest. Compounds inhibiting leucine-rich repeat kinase enzyme activity, September 12 2014. WO Patent App. PCT/US2014/018,888.

[306] Robert D. Miller, Victor Y. Lee, and Christopher R. Moylan. Substituted azole derivatives as nonlinear optical chromophores. *Chemistry of Materials*, 6(7):1023–1032, jul 1994.

[307] Masafumi Minoshima, Tetsuaki Matsumoto, and Kazuya Kikuchi. Development of a fluorogenic probe based on a DNA staining dye for continuous monitoring of the histone deacetylase reaction. *Analytical Chemistry*, 86(15):7925–7930, aug 2014.

[308] Yasuo Miyata, Tohru Nishinaga, and Koichi Komatsu. Synthesis and structural, electronic, and optical properties of oligo(thienylfuran)s in comparison with oligothiophenes and oligofurans. *The Journal of Organic Chemistry*, 70(4):1147–1153, feb 2005.

[309] Michiyo Mochizuki, Masakuni Kori, Katsumi Kobayashi, Takahiko Yano, Yuu Sako, Maiko Tanaka, Naoyuki Kanzaki, Albert C. Gyorkos, Christopher P. Corrette, Suk Young Cho, Scott A. Pratt, and Kazuyoshi Aso. Design and synthesis of benzimidazoles as novel corticotropin-releasing factor 1 receptor antagonists. *Journal of Medicinal Chemistry*, 59(6):2551–2566, mar 2016.

[310] Anna-Maria Monforte, Patrizia Logoteta, Stefania Ferro, Laura De Luca, Nunzio Iraci, Giovanni Maga, Erik De Clercq, Christophe Pannecouque, and Alba Chimirri. Design, synthesis, and structure–activity relationships of 1,3-dihydrobenzimidazol-2-one analogues as anti-HIV agents. *Bioorganic & Medicinal Chemistry*, 17(16):5962–5967, aug 2009.

[311] Rémy Morgentin, Georges Pasquet, Pascal Boutron, Frédéric Jung, Maryannick Lamorlette, Mickaël Maudet, and Patrick Plé. Strategic studies in the syntheses of novel 6, 7-substituted quinolones and 7- or 6-substituted 1, 6- and 1, 7-naphthyridones. *Tetrahedron*, 64(12):2772–2782, mar 2008.

[312] R.M. Moslin, D.S. Weinstein, S.T. Wrobleski, Y. Zhang, J.S. Tokarski, M.E. Mertzman, and C. Liu. Alkyl-amide-substituted pyridyl compounds useful as modulators of il-12, il-23 and/or ifnalpha responses, May 14 2015. WO Patent App. PCT/US2014/011,769.

[313] R.M. MOSLIN, D.S. Weinstein, S.T. Wrobleski, Y. Zhang, J.S. Tokarski, M.E. Mertzman, and C. Liu. Alkyl-amide-substituted pyridyl compounds useful as modulators of il-12, il-23 and/or ifnalpha responses, May 14 2015. WO Patent App. PCT/US2014/011,769.

[314] P. Murer, K. Dormann, F.L. Benedito, G. BATTAGLIARIN, S. Metz, U. HEINEMEYER, C. Lennartz, G. Wagenblast, S. Watanabe, and T. Gessner. Monosubstituted diazabenzimidazole carbene metal complexes for use in organic light emitting diodes, January 8 2015. WO Patent App. PCT/EP2014/064,054.

[315] T. NABATA, S. Mizuta, T. Miyano, K. Sato, A. Kihara, S. Kazama, and Y. Katayama. Electronic device, January 29 2015. US Patent App. 14/374,646.

[316] N. Negoro, Y. Terao, S. Mikami, and T. Yukawa. Novel fused cyclic compound and use thereof, December 16 2010. WO Patent App. PCT/JP2010/059,999.

[317] M.H. Nettekoven and S. Schmitt. 5-methoxy-8-aryl-[1,2,4] triazolo [1,5-a] pyridine derivatives as adenosine receptor antagonists, April 17 2003. WO Patent App. PCT/EP2002/010,917.

[318] John L. Neumeyer, Nandkishore Baindur, Hyman B. Niznik, H. C. Guan, and Philip Seeman. (.+-.)-3-allyl-6-bromo-7,8-dihydroxy-1-phenyl-2,3,4,5-tetrahydro-1h-3-benzazepine, a new high affinity d1 dopamine receptor ligand: synthesis and structure-activity relationship. *Journal of Medicinal Chemistry*, 34(12):3366–3371, dec 1991.

[319] J. Neyts, J. Paeshuyse, O. Chavignon, J.M. Chezal, V. Gaumet, A. Gueiffier, and J.C. Teulade. Imidazo [1, 2-a] pyrrolo [3, 2-c] pyridine compounds useful as pestivirus inhibitors, June 19 2008. WO Patent App. PCT/BE2007/000,127.

[320] Olaia Nieto-García and Ricardo Alonso. IMDAF/aromatization path of halogenated furylacrylamides and furylpropiolamides to dihydroisoquinolin-1(2h)-ones. *The Journal of Organic Chemistry*, 78(6):2564–2570, mar 2013.

[321] Olaia Nieto-García and Ricardo Alonso. Synthesis and cytotoxicity of (+/-)-7,9-dideoxypancratistatin analogues. *Org. Biomol. Chem.*, 11(3):515–522, 2013.

[322] Toshiharu Noji, Hideto Fujiwara, Kentaro Okano, and Hidetoshi Tokuyama. Synthesis of substituted indoline and carbazole by benzyne-mediated cyclization–functionalization. *Organic Letters*, 15(8):1946–1949, apr 2013.

[323] M. Nomura, N. Tomura, A. Kawahara, and H. Daido. Pest control composition, June 26 2008. WO Patent App. PCT/JP2007/001,355.

[324] M. Nomura, N. Tomura, A. Kawahara, and H. Daido. Pesticidal composition for vegetable seed or harvest, and use of the same, June 26 2008. WO Patent App. PCT/JP2007/001,415.

[325] Michikazu Nomura, Naofumi Tomura, Atsuko Kawahara, and Hidenori Daido. *Jpn. Kokai Tokkyo Koho*, chapter Insecticide compositions containing amides., page 163pp. Number JP2010047480A. Mitsui Chemicals Inc., Japan ., Mar 2010. Copyright (C) 2017 American Chemical Society (ACS). All Rights Reserved.

[326] Michikazu Nomura, Naofumi Tomura, Atsuko Kawahara, and Hidenori Daido. *Jpn. Kokai Tokkyo Koho*, chapter Pest control compositions containing amides., page 139pp. Number JP2010047479A. Mitsui Chemicals Inc., Japan ., Mar 2010. Copyright (C) 2017 American Chemical Society (ACS). All Rights Reserved.

[327] Veronika Novakova, Miroslav Miletin, Kamil Kopecky, and Petr Zimcik. Red-emitting dyes with photophysical and photochemical properties controlled by pH. *Chemistry - A European Journal*, 17(50):14273–14282, nov 2011.

[328] Koji Ochiai, Satoshi Takita, Tomohiko Eiraku, Akihiko Kojima, Kazuhiko Iwase, Tetsuya Kishi, Kazunori Fukuchi, Tokutaro Yasue, David R. Adams, Robert W. Allcock, Zhong Jiang, and Yasushi Kohno. Phosphodiesterase inhibitors. part 3: Design, synthesis and structure–activity relationships of dual PDE3/4-inhibitory fused bicyclic heteroaromatic-dihydropyridazinones with anti-inflammatory and bronchodilatory activity. *Bioorganic & Medicinal Chemistry*, 20(5):1644–1658, mar 2012.

[329] Christopher J. O'Donnell, Bruce N. Rogers, Brian S. Bronk, Dianne K. Bryce, Jotham W. Coe, Karen K. Cook, Allen J. Duplantier, Edelweiss Evrard, Mihaly Hajos, William E. Hoffmann, Raymond S. Hurst, Noha Maklad, Robert J. Mather, Stafford McLean, Frank M. Nedza, Brian T. O'Neill, Langu Peng, Weimin Qian, Melinda M. Rottas, Steven B. Sands, Anne W. Schmidt, Alka V. Shrikhande, Douglas K. Spracklin, Diane F. Wong, Andy Zhang, and Lei Zhang. Discovery of 4-(5-methyloxazolo[4, 5-b]pyridin-2-yl)-1, 4-diazabicyclo[3.2.2]nonane (CP-810, 123), a novel α7 nicotinic acetylcholine receptor agonist for the treatment of cognitive disorders in schizophrenia: Synthesis, sar development, and in vivo efficacy in cognition models. *Journal of Medicinal Chemistry*, 53(3):1222–1237, feb 2010.

[330] N. OI, N. Yamamoto, M. Suzuki, Y. NAKATANI, T. Suhara, M. CHO, T. Fukumura, M. Higuchi, T. MINAMIMOTO, J. Maeda, et al. [11 c] and [18f] labeled 1,3-diphenyl-5-(pyrimidin-2-yl)-pyridin-2(1 h)-one derivatives and their use for pet imaging of the ampa receptor, October 9 2014. WO Patent App. PCT/JP2014/060,233.

[331] Levente Ondi, Olivier Lefebvre, and Manfred Schlosser. Brominated 4-(trifluoromethyl)pyrimidines: A convenient access to versatile intermediates. *European Journal of Organic Chemistry*, 2004(20):4280–4280, oct 2004.

[332] Stefan Ortgies and Alexander Breder. Selenium-catalyzed oxidative c(sp2)–h amination of alkenes exemplified in the expedient synthesis of (aza-)indoles. *Organic Letters*, 17(11):2748–2751, jun 2015.

[333] K. Ouhenia-Ouadahi, R. Yasukuni, P. Yu, G. Laurent, C. Pavageau, J. Grand, J. Guérin, A. Léaustic, N. Félidj, J. Aubard, K. Nakatani, and R. Métivier. Photochromic–fluorescent–plasmonic nanomaterials: towards integrated three-component photoactive hybrid nanosystems. *Chem. Commun.*, 50(55):7299–7302, 2014.

[334] Attila Paczal, Balázs Bálint, Csaba Wéber, Zoltán B. Szabó, Levente Ondi, Isabelle Theret, Frédéric De Ceuninck, Catherine Bernard, Alain Ktorza, Francoise Perron-Sierra, and András Kotschy. Structure–activity relationship of azaindole-based glucokinase activators. *Journal of Medicinal Chemistry*, 59(2):687–706, jan 2016.

[335] Andreas Marc Palmer, Gabriela Münch, Christof Brehm, Peter Jan Zimmermann, Wilm Buhr, Martin Philipp Feth, and Wolfgang Alexander Simon. 5-substituted 1h-pyrrolo[3,2-b]pyridines as inhibitors of gastric acid secretion☆. *Bioorganic & Medicinal Chemistry*, 16(3):1511–1530, feb 2008.

[336] Brian D. Palmer and William A. Denny. Synthesis and reactions of brominated 2-nitroimidazoles. *Journal of the Chemical Society, Perkin Transactions 1*, (1):95, 1989.

[337] Barbara Parrino, Anna Carbone, Marina Muscarella, Virginia Spanò, Alessandra Montalbano, Paola Barraja, Alessia Salvador, Daniela Vedaldi, Girolamo Cirrincione, and Patrizia Diana. 11h-pyrido[3′,2′:4,5]pyrrolo[3,2-c]cinnoline and pyrido[3′,2′:4,5]pyrrolo[1,2-c][1,2,3]benzotriazine: Two new ring systems with antitumor activity. *Journal of Medicinal Chemistry*, 57(22):9495–9511, nov 2014.

[338] K. Paruch, M. Petrujova, and V. NEMEC. Furopyridines utilisées en tant qu'inhibiteurs de protéine kinases, November 5 2015. WO Patent App. PCT/CZ2015/000,038.

[339] L. Patient, D. Evans, I. Simpson, and A. POWELL. New compounds, September 18 2014. WO Patent App. PCT/GB2014/050,764.

[340] William W. Paudler and Misa V. Jovanovic. Bromination of some pyridine and diazine n-oxides. *The Journal of Organic Chemistry*, 48(7):1064–1069, apr 1983.

[341] Kevin C Pels and Veljko Dragojlovic. Solvent-free phase-vanishing reactions with PTFE (teflon®) as a phase screen. *Beilstein Journal of Organic Chemistry*, 5, dec 2009.

[342] Xuefeng Peng, Jian-Guo Deng, and Hai-Bing Xu. Substituent and solvent effects on the fluorescent and photochromic properties of 2-(2-pyridyl)imidazole containing diarylethene derivatives. *RSC Advances*, 3(46):24146, 2013.

[343] Maria Grazia Perrone, Paola Vitale, Andrea Panella, Cosimo G. Fortuna, and Antonio Scilimati. General role of the amino and methylsulfamoyl groups in selective cyclooxygenase(COX)-1 inhibition by 1,4-diaryl-1,2,3-triazoles and validation of a predictive pharmacometric PLS model. *European Journal of Medicinal Chemistry*, 94:252–264, apr 2015.

[344] L. M. Pevzner. Synthesis of isomeric bromo(diethoxyphosphorylmethyl)furans. *Russian Journal of General Chemistry*, 79(3):362–372, mar 2009.

[345] J.A. Picard, B.D. Roth, and D.R. Sliskovic. Trans-6-(2-(n-heteroaryl-3,5-disubstituted)pyrazol-4-yl)-ethyl)-or ethenyl)tetrahydro-4-hydroxypyran-2-one inhibitors of cholesterol biosynthesis, September 18 1990. US Patent 4,957,971.

[346] Cinzia Di Pietro, Scolastica Serroni, Sebastiano Campagna, Maria Teresa Gandolfi, Roberto Ballardini, Stefano Fanni, Wesley R. Browne, and Johannes G. Vos. Proton controlled intramolecular communication in dinuclear ruthenium(II) polypyridine complexes. *Inorganic Chemistry*, 41(11):2871–2878, jun 2002.

[347] MR Prabhath, Julia Romanova, Richard J Curry, S Ravi P Silva, and Peter D Jarowski. The role of substituent effects in tuning metallophilic interactions and emission energy of bis-4-(2-pyridyl)-1, 2, 3-triazolatoplatinum (ii) complexes. *Angewandte Chemie International Edition*, 54(27):7949–7953, 2015.

[348] R. Pracitto, J.F. Kadow, J.A. Bender, B.R. Beno, K. Grant-Young, Y. Han, P. Hewawasam, A. Nickel, K.E. Parcella, K.S. Yeung, et al. Compounds for the treatment of hepatitis c, September 15 2011. WO Patent App. PCT/US2010/026,786.

[349] G. K. Surya Prakash, Thomas Mathew, Dushyanthi Hoole, Pierre M. Esteves, Qi Wang, Golam Rasul, and George A. Olah. N-halosuccinimide/BF3-h2o, efficient electrophilic halogenating systems for aromatics. *Journal of the American Chemical Society*, 126(48):15770–15776, dec 2004.

[350] A.V. Purandare, J.W. Grebinski, A. Hart, J. Inghrim, G. Schroeder, and H. Wan. Jak2 inhibitors and their use for the treatment of myeloproliferative diseases and cancer, March 10 2011. WO Patent App. PCT/US2010/047,621.

[351] Rintje Raap and Ronald G. Micetich. Penicillins and cephalosporins from isothiazolylacetic acids. *Journal of Medicinal Chemistry*, 11(1):70–73, jan 1968.

[352] Potharaju Raju, Ganesan Gobi Rajeshwaran, Meganathan Nandakumar, and Arasambattu K. Mohanakrishnan. Unusual reactivity of aryl aldehydes with triethyl phosphite and zinc bromide: A facile preparation of epoxides, benzisoxazoles, and α-hydroxy phosphonate esters. *European Journal of Organic Chemistry*, 2015(16):3513–3523, apr 2015.

[353] El Rakib, Bassou Oulemda, Said Abouricha, Latifa Bouissane, Hassan Mouse, and Abdelmajid Zyad. In vitro cytotoxicity evaluation of some substituted indazole derivatives. *Letters in Drug Design & Discovery*, 4(7):467–470, oct 2007.

[354] S. Ravula, D.M. Swanson, B.M. SAVALL, and M.K. Ameriks. Benzimidazolone and benzothiazolone compounds and their use as ampa receptor modulators, November 3 2016. WO Patent App. PCT/US2016/029,780.

[355] T.M. Raynham, T.R. Hammonds, J.H. Gilliatt, M.D. Charles, G.A. Pave, C.H. Foxton, J.L. Carr, and N.S. Mistry. Amino-ethyl-amino-aryl (aeaa) compounds and their use, November 8 2007. WO Patent App. PCT/GB2007/001,537.

[356] Philip Reigan, Abdul Gbaj, Ian J. Stratford, Richard A. Bryce, and Sally Freeman. Xanthine oxidase-activated prodrugs of thymidine phosphorylase inhibitors. *European Journal of Medicinal Chemistry*, 43(6):1248–1260, jun 2008.

[357] M.D. Robinson, M. Robertson, H. Isaac, O. Guinan, T.J. Melendez, D. Berkenstock, and J. Mann. Adaptive image acquisition and processing with image analysis feedback, February 28 2013. US Patent App. 13/219,461.

[358] E.T. ROMEO, M.R. Machacek, B.W. TROTTER, T.A. MILLER, B.M. Andresen, N.J. ANTHONY, B.M. Taoka, and Y. Liu. Amino-pyridine-containing spleen tyrosine kinase (syk) inhibitors, November 8 2012. WO Patent App. PCT/US2012/035,725.

[359] F.A. Romero, S. Magnuson, R. Pastor, V.H.W. Tsui, J. Murray, T. CRAWFORD, B.K. Albrecht, A. Cote, A.M. Taylor, K.W. LAI, et al. 4,5,6,7-tetrahydro-1 h-pyrazolo[4,3-c]pyridin-3-amine compounds as cbp and/or ep300 inhibitors, June 2 2016. WO Patent App. PCT/US2015/062,794.

[360] Emilie Rossignol, Ali Youssef, Pascale Moreau, Michelle Prudhomme, and Fabrice Anizon. Synthesis of aminopyrimidylindoles structurally related to meridianins. *Tetrahedron*, 63(41):10169–10176, oct 2007.

[361] T. Rueckle, X. Jiang, P. Gaillard, D. Church, and T.G. Vallotton. Azolidinone-vinyl fused-benzene derivatives, December 7 2010. US Patent 7,846,925.

[362] Heinrich Rueeger, Rainer Lueoend, Olivier Rogel, Jean-Michel Rondeau, Henrik M" obitz, Rainer Machauer, Laura Jacobson, Matthias Staufenbiel, Sandrine Desrayaud, and Ulf Neumann. Discovery of cyclic sulfone hydroxyethylamines as potent and selective β-site APP-cleaving enzyme 1 (BACE1) inhibitors: Structure-based design and in vivo reduction of amyloid β-peptides. *Journal of Medicinal Chemistry*, 55(7):3364–3386, apr 2012.

[363] Seoung ryoung Choi, Anupam Pradhan, Nicholas L. Hammond, Amar G. Chittiboyina, Babu L. Tekwani, and Mitchell A. Avery. Design, synthesis, and biological evaluation of-Plasmodium falciparumLactate dehydrogenase inhibitors. *Journal of Medicinal Chemistry*, 50(16):3841–3850, aug 2007.

[364] Giorgio Della Sala, Daniela Capozzo, Irene Izzo, Assunta Giordano, Antonella Iommazzo, and Aldo Spinella. Synthesis of antifungal n-isoprenyl-indole alkaloids from the fungus aporpium caryae. *Tetrahedron Letters*, 43(49):8839–8841, dec 2002.

[365] N. Saldabol, J. Popelis, O. Lando, and V. Slavinska. 2-(2-furyl)imidazo[1,2-a]pyrimidine synthesis and electrophilic substitution reactions. *Chemistry of Heterocyclic Compounds*, 42(4):495–499, apr 2006.

[366] Ramesh C. Samanta and Hisashi Yamamoto. Selective halogenation using an aniline catalyst. *Chemistry - A European Journal*, 21(34):11976–11979, jul 2015.

[367] Ramesh C. Samanta and Hisashi Yamamoto. Selective halogenation using an aniline catalyst. *Chemistry - A European Journal*, 21(34):11976–11979, jul 2015.

[368] Mª Ascensión Sanz-Tejedor, Ángela Meana, Justo F. Rodríguez, and José L. García-Ruano. Efficient regioselective preparationof monobromo and bromoiodo hydroxy pyridines from dibromoderivativesvia bromine-lithium exchange. *Synlett*, (11):1678–1682, 2003.

[369] Mª Ascensión Sanz-Tejedor, Ángela Meana, Justo F. Rodríguez, and José L. García-Ruano. Efficient regioselective preparationof monobromo and bromoiodo hydroxy pyridines from dibromoderivativesvia bromine-lithium exchange. *Synlett*, (11):1678–1682, 2003.

[370] Nobuhiro Sato. Studies on pyrazines. 4.. the synthesis of 2-hydroxy-6-phenylpyrazine and its derivatives. *Journal of Heterocyclic Chemistry*, 15(4):665–670, jun 1978.

[371] G. Saxty, V. Berdini, C.W. Murray, E.J.E. Freyne, Y.A.E. Ligny, P.G.A. BONNET, B. Wroblowski, and A. Papanikos. Imidazo [1,2-a]pyridine derivatives as fgfr kinase inhibitors for use in therapy, October 21 2010. WO Patent App. PCT/GB2010/050,618.

[372] E. Schwiebert, J. STREIFF, J. Dixon, and H. Gao. Coumarin derivatives and methods of use in treating cystic fibrosis, chronic obstructive pulmonary disease, and misfolded protein disorders, September 25 2014. WO Patent App. PCT/US2014/027,079.

[373] J.D. Scott, A.W. Stamford, E.J. Gilbert, J.N. Cumming, U. Iserloh, J.A. Misiaszek, and G. Li. Iminothiadiazine dioxide compounds as bace inhibitors, compositions, and their use, April 14 2011. WO Patent App. PCT/US2010/051,553.

[374] Keitaro Senga, Darrell E. O'Brien, Mieka B. Scholten, Thomas Novinson, Jon P. Miller, and Roland K. Robins. Synthesis and enzymic activity of various substituted pyrazolo[1,5-a]-1,3,5-triazines as adenosine cyclic 3',5'-phosphate phosphodiesterase inhibitors. *Journal of Medicinal Chemistry*, 25(3):243–249, mar 1982.

[375] R. Sharma, U. Ghosh, T. More, M. Kulkarni, K. BAJAJ, S. BURUDKAR, and Z. RIZVI. Imidazopyridine compounds and uses thereof, May 30 2014. WO Patent App. PCT/IB2012/056,580.

[376] Sudhir R. Shengule, Anthony Willis, and Stephen G. Pyne. Synthesis of spirocyclic azacycles from the cyclization of furan tethered n-acyliminium ions. *Tetrahedron*, 68(4):1207–1215, jan 2012.

[377] Rajesha Shetty1, S. Syed Shafi, and Yuvaraj. K. Synthesis and antifungal activity studies of novel 3,4,5-trisubstitutedisoxazole derivatives. *Journal of Pharmacy Research*, 8(3):289–295, 2014.

[378] M. J. Shiao, P. Shieh, and J. S. Lai. *Chinese Chem. Soc. (Taipei, Taiwan)*, 35(3):233–236, 1988.

[379] Hirotaka Shibuya, Yoshihiro Hamamoto, Yang Cai, and Isao Kitagawa. Chem. pharm. bull. *Trends in Heterocyclic Chemistry*, 35(2):924–927, 1987.

[380] Y. Shishido, M. Ohmi, and K. Ando. Azaspiro derivatives as trpm8 antagonists, September 17 2015. WO Patent App. PCT/JP2015/001,454.

[381] Baldev Singh, Edward R. Bacon, George Y. Lesher, Shaughnessy Robinson, Patrick O. Pennock, Donald C. Bode, Edward D. Pagani, Ross G. Bentley, Mary J. Connell, Linda T. Hamel, and Paul J. Silver. Novel and potent adenosine 3',5'-cyclic phosphate phosphodiesterase III inhibitors: Thiazolo[4,5-b][1,6]naphthyridin-2-ones. *Journal of Medicinal Chemistry*, 38(14):2546–2550, jul 1995.

[382] M.P. Singh and M.N. Chatterjee. Oligoheteroaromatic luminiscent assemblies as high-affinity dna sequence-directed ligands, April 26 2007. WO Patent App. PCT/CA2006/001,724.

[383] L. Sipos. A process for preparing a polymer having a 2,5-furandicarboxylate moiety within the polymer backbone and such (co)polymers, October 5 2011. EP Patent App. EP20,090,796,484.

[384] A.J. SMITH, A.R. NOVAK, D.M. Evans, H.J. EDWARDS, M.J. Stocks, R.L. DAVIE, S.L. MARSH, and S.T. Hodgson. N-((heteroarylmethyl)-heteroaryl-carboxamide derivatives as plasma kallikrein inhibitors, June 2 2016. WO Patent App. PCT/GB2015/053,610.

[385] H. Smits, T. Pitterna, O.F. Hueter, A. Edmunds, A. Stoller, and P.J.M. Jung. Procédé pour la préparation d'amides à partir d'anilines encombrées contenant un groupe perhaloalkyle, October 9 2014. WO Patent App. PCT/EP2014/056,518.

[386] E. Sonnenschein, M. Sonnenschein, and L. Crainich. Stapler for endoscopes, March 13 2003. US Patent App. 10/030,018.

[387] Chad E. Stephens, Ruth Ann Day, and Jacqueline A. Blake. Convenient and improved halogenationof 3,5-diarylisoxazoles UsingN-halosuccinimides. *Synthesis*, (10):1586–1590, 2003.

[388] A. Sudau, J. Neumann, and J. BENTING. Thienylpyri(mi)dinylpyrazole, December 27 2012. WO Patent App. PCT/EP2012/061,741.

[389] M. Sugawara, Sugawara Masamori, T. Danjo, Tan Shangyou Hao, T. Sawada, Zetian Guishi, H. Ishida, Ishida Hiroshi, O. Saku, Zuojiu mo, et al. Hedgehog signal inhibitor, February 17 2011. WO Patent App. PCT/JP2010/063,655.

[390] Young-Ger Suh, Yong-Sil Lee, Kyung-Hoon Min, Ok-Hui Park, Jin-Kwan Kim, Ho-Sun Seung, Seung-Yong Seo, Bo-Young Lee, Yeon-Hee Nam, Kwang-Ok Lee, Hee-Doo Kim, Hyeung-Geun Park, Jeewoo Lee, Uhtaek Oh, Ju-Ok Lim, Sang-Uk Kang, Min-Jung Kil, Jae yeon Koo, Song Seok Shin, Yung-Hyup Joo, Jin Kwan Kim, Yeon-Su Jeong, Sun-Young Kim, and Young-Ho Park. Novel potent antagonists of transient receptor potential channel, vanilloid subfamily member 1: structure-activity relationship of 1,3-diarylalkyl thioureas possessing new vanilloid equivalents. *Journal of Medicinal Chemistry*, 48(18):5823–5836, sep 2005.

[391] Hua Sun, Yazhou Zhang, Weina Ding, Xue Zhao, Xiaotong Song, Dong Wang, Yashan Li, Kailin Han, Yang Yang, Ying Ma, Runling Wang, Dong Wang, and Peng Yu. Inhibitory activity evaluation and mechanistic studies of tetracyclic oxindole derivatives as α-glucosidase inhibitors. *European Journal of Medicinal Chemistry*, 123:365–378, nov 2016.

[392] N. Sun and W.W. Zhang. Flame resistant polyamide resin composition and articles comrpising the same, February 2 2012. WO Patent App. PCT/US2011/030,146.

[393] R. Sun, A.B. Cooper, Y. Deng, T. Wang, Y. Nan, H.Y. Zhu, S.B. Boga, X. Gao, J.M. Kelly, S. Paliwal, et al. Heterocyclic compounds and use thereof as erk inhibitors, December 24 2008. WO Patent App. PCT/US2008/007,509.

[394] K. Suzuki, T. Yamaguchi, A. Tamura, T. Nishizawa, and M Yamaguchi. Nitrogen-containing aromatic heterocyclyl com- pound, 2010. WO Patent App. 2010/004972 A1.

[395] Kazuharu Suzuki, Masaaki Tomura, Shoji Tanaka, and Yoshiro Yamashita. Synthesis and characterization of novel strong electron acceptors: bithiazole analogues of tetracyanodiphenoquinodimethane (TCNDQ). *Tetrahedron Letters*, 41(43):8359–8364, oct 2000.

[396] Keiko Suzuki, Takahiro Yamaguchi, Akihiro Tamura, Tomohiro Nishizawa, and Mitsuhiro Yamaguchi. *PCT Int. Appl.*, chapter Preparation of nitrogen-containing aromatic heterocyclyl compounds as hypolipidemics., page 226pp. Number WO2010004972A1. Daiichi Sankyo Company, Limited, Japan ., Jan 2010. Copyright (C) 2017 American Chemical Society (ACS). All Rights Reserved.

[397] Murat Süküroglu, Burcu Çaliskan Ergün, Serdar ünlü, M. Fethi Sahin, Esra Küpeli, Erdem Yesilada, and Erden Banoglu. Synthesis, analgesic, and anti-inflammatory activities of [6-(3,5-dimethyl-4-chloropyrazole-1-yl)-3(2h)-pyridazinon-2-yl]acetamides. *Archives of Pharmacal Research*, 28(5):509–517, may 2005.

[398] Shizuka Takami and Masahiro Irie. Synthesis and photochromic properties of novel yellow developing photochromic compounds. *Tetrahedron*, 60(29):6155–6161, 2004.

[399] Nuria A. Tamayo, Mark H. Norman, Michael D. Bartberger, Fang-Tsao Hong, Yunxin Bo, Longbin Liu, Nobuko Nishimura, Kevin C. Yang, Seifu Tadesse, Christopher Fotsch, Jie Chen, Samer Chmait, Rod Cupples, Clarence Hale, Steven R. Jordan, David J. Lloyd, Glenn Sivits, Gwyneth Van, and David J. St. Jean. Small molecule disruptors of the glucokinase–glucokinase regulatory protein interaction: 5. a novel aryl sulfone series, optimization through conformational analysis. *Journal of Medicinal Chemistry*, 58(11):4462–4482, jun 2015.

[400] Naohito Tanizawa. *Jpn. Kokai Tokkyo Koho*, chapter Preparation of, 2,5-dihalobenzotrichlorides., page 4 pp. Number JP03027334A. Ihara Chemical Industry Co., Ltd., Japan ., Feb 1991. Copyright (C) 2017 American Chemical Society (ACS). All Rights Reserved.

[401] J.R. Tata, K.T. Chapman, J.L. Duffy, N.J. Kevin, Y. Cheng, T.A. Rano, F. Zhang, T. Huening, B.A. Kirk, Z. Lu, et al. Gamma-hydroxy-2-(fluoroalkylaminocarbonyl)-1-piperazinepentanamides intervenant comme inhibiteurs de la protease du vih, May 31 2001. WO Patent App. PCT/US2000/032,089.

[402] Miroslav Terinek and Andrea Vasella. Synthesis ofC(2)-substitutedmanno-configured tetrahydroimidazopyridines and their evaluation as inhibitors of snailβ-mannosidase. *Helvetica Chimica Acta*, 86(10):3482–3509, oct 2003.

[403] W.K. Tsai. Content procurement architecture, June 26 2008. US Patent App. 11/962,006.

[404] B.C.A.A. Van, G.P.J.M. Van, and J.B.M. Rewinkel. Heterocyclic derivatives and their use as antithrombotic agents, October 29 1998. WO Patent App. PCT/EP1998/002,455.

[405] Jacobus P.D. van Veldhoven, Lisa C.W. Chang, Jacobien K. von Frijtag Drabbe Künzel, Thea Mulder-Krieger, Regina Struensee-Link, Margot W. Beukers, Johannes Brussee, and Adriaan P. IJzerman. A new generation of adenosine receptor antagonists: From di- to trisubstituted aminopyrimidines. *Bioorganic & Medicinal Chemistry*, 16(6):2741–2752, mar 2008.

[406] Ermes Vanotti, Marina Caldarelli, Teresa Disingrini, Marcella Nesi, Marco Tatò, Paola Vianello, and Maria Menichincheri. Regioselective halogenation of aminopyrimidinyl-pyrrole carboxylic acid derivatives. *Tetrahedron*, 65(50):10418–10423, dec 2009.

[407] U. Velaparthi, C.P. Darne, P. Liu, M.D. Wittman, B.C. Pearce, E.M.V. Araujo, B. Dasgupta, J.S. Nair, S.K. Janakiraman, C.R. Rachamreddy, et al. Novel substituted pyrazolo-piperazines as casein kinase 1 d/e inhibitors, May 14 2015. US Patent App. 14/541,343.

[408] Marie-Claude Viaud, Patricia Jamoneau, Laurence Savelon, and Gerald Guillaumet. Synthesis of 6-substituted 2-phenyloxazolo[4,5-b]pyridines. *Heterocycles*, 41(12):2799–809, 1995. Copyright (C) 2017 American Chemical Society (ACS). All Rights Reserved.

[409] J.B. Vidal, D.J.A. Alonso, A.M.A. Buil, P.R. Eastwood, T.C. Esteve, T.M.E. Lozoya, R.S. Roberts, G.L. Vidal, R.J. Gonzalez, and C.M. Mir. New crth2 antagonists, January 24 2013. WO Patent App. PCT/EP2012/063,603.

[410] J.B. Vidal, T.C. Esteve, P.L. Soca, and P.R. Eastwood. Pyrazine derivatives useful as adenosine receptor antagonists, February 15 2007. WO Patent App. PCT/EP2006/007,318.

[411] J.B. Vidal, T.C. Esteve, P.L. Soca, and P.R. Eastwood. Pyrazine derivatives useful as adenosine receptor antagonists, February 15 2007. WO Patent App. PCT/EP2006/007,318.

[412] J.B. Vidal, T.C. Esteve, P.L. Soca, and P.R. Eastwood. Pyrazine derivatives useful as adenosine receptor antagonists, February 15 2007. WO Patent App. PCT/EP2006/007,318.

[413] J.B. Vidal, C.J.C. Fernandez, S.M. Erra, I.N. Aguilar, C.M. Mir, and M.I. Carranco. Compounds as syk kinase inhibitors, August 22 2012. EP Patent App. EP20,110,382,043.

[414] J.B. Vidal, P.S. Fonquerna, P.R. Eastwood, B.J. Aiguade, F.A. Cardus, M.I. Carranco, R.J. Gonzalez, and A.S. Paredes. Imidazopyridine derivatives as a2b adenosine receptor antagonists, April 12 2007. WO Patent App. PCT/EP2006/009,620.

[415] J.B. Vidal, P.S. Fonquerna, P.R. Eastwood, B.J. Aiguade, F.A. Cardus, M.I. Carranco, R.J. Gonzalez, and A.S. Paredes. Imidazopyridine derivatives as a2b adenosine receptor antagonists, April 12 2007. WO Patent App. PCT/EP2006/009,620.

[416] Gábor Vlád and István T. Horváth. Improved synthesis of 2,2'-bipyrimidine. *The Journal of Organic Chemistry*, 67(18):6550–6552, sep 2002.

[417] Duc Duy Vo and Mikael Elofsson. Total synthesis of viniferifuran, resveratrol-piceatannol hybrid, anigopreissin a and analogues - investigation of demethylation strategies. *Advanced Synthesis & Catalysis*, 358(24):4085–4092, dec 2016.

[418] A. Vogel and F. Troxler. *F. Helv. Chim. Acta*, 58(3):761–771, 1975.

[419] F. VOSS, S. RITTER, S. Nordhoff, S. Wachten, S. OBERBÖRSCH, and A. Kless. Annelated pyrroles and their use as crac inhibitors, February 19 2015. WO Patent App. PCT/EP2014/002,218.

[420] Yasuhiro Wada, Hiromitsu Shirahashi, Taisuke Iwanami, Masami Ogawa, Seiji Nakano, Akifumi Morimoto, Ken ichi Kasahara, Eiichi Tanaka, Yoshio Takada, Shigeki Ohashi, Mutsuhiro Mori, and Satoshi Shuto. Discovery of novel indazole derivatives as highly potent and selective human β3-adrenergic receptor agonists with the possibility of having no cardiovascular side effects. *Journal of Medicinal Chemistry*, 58(15):6048–6057, aug 2015.

[421] Peter J. Wagner and Lingling Wang. Electronic effects of ring substituents on triplet benzylic biradicals. *Organic Letters*, 8(4):645–647, feb 2006.

[422] Zehong Wan, Adrian Hall, Yun Jin, Jia-Ning Xiang, Eric Yang, Andrew Eatherton, Beverley Smith, Guang Yang, Haihua Yu, Ju Wang, Liang Ye, Lit-Fui Lau, Ting Yang, William Mitchell, Wei Cai, Xiaomin Zhang, Yingxia Sang, Yonghui Wang, Zhaolong Tong, Ziqiang Cheng, Ishrut Hussain, John D. Elliott, and Yasuji Matsuoka. Pyridazine-derived γ-secretase modulators. *Bioorganic & Medicinal Chemistry Letters*, 21(13):4016–4019, jul 2011.

[423] L. Wang, J. Pratt, L.A. Hasvold, D. Liu, Y. Dai, S.D. Fidanze, J.H. Holms, R.A. Mantei, K.F. McDaniel, G.S. Sheppard, et al. Dihydro-pyrrolopyridinone inhibitors, September 18 2014. US Patent App. 14/206,028.

[424] L. Wang and T. WANG. Substituted pyridine compounds and methods of use, March 12 2015. WO Patent App. PCT/US2014/053,017.

[425] X.J. Wang, T. Wirth, T. Nicola, L. Zhang, R. Frutos, Y. Xu, D. Krishnamurihy, L. Nummy, R. Varsolona, C. Senanayake, et al. Synthesis of 6,7-dihydro-5h-imidazo[1,2-a]imidazole-3-sulfonic acid amides, February 2 2006. US Patent App. 11/188,377.

[426] Zhi-Liang Wei, Pavel A. Petukhov, Yingxian Xiao, Werner Tückmantel, Clifford George, Kenneth J. Kellar, and Alan P. Kozikowski. Synthesis, nicotinic acetylcholine receptor binding affinities, and molecular modeling of constrained epibatidine analogues. *Journal of Medicinal Chemistry*, 46(6):921–924, mar 2003.

[427] Birgit Wilding, Carina Vidovic, and Norbert Klempier. A convenient synthetic route to substituted pyrrolo[2,3- b]pyridines via a novel ethylene-bridged compound. *Tetrahedron Letters*, 56(47):6606–6609, nov 2015.

[428] R.R. Wilkening, D.L. Parker, K.J. Wildonger, D. Meng, and R.W. Ratcliffe. Estrogen receptor modulators, May 30 2002. WO Patent App. PCT/US2001/044,010.

[429] M.G. Woll, G. Chen, S. Choi, A. DAKKA, S. Huang, G.M. Karp, C.S. Lee, C. Li, J. Narasimhan, N. Naryshkin, et al. Compounds for treating spinal muscular atrophy, July 4 2013. WO Patent App. PCT/US2012/071,899.

[430] H. Wu, J. Lin, Y. Li, C. WEI, and S. Chen. Quinoline derivatives as smo inhibitors, October 1 2015. WO Patent App. PCT/CN2015/074,268.

[431] Yongqian Wu. *Faming Zhuanli Shenqing*, chapter Preparation of pyrimidine derivatives as PI3K inhibitors., page 47pp. Number CN105541792A. Xuanzhu Pharma Co., Ltd., Peop. Rep. China ., May 2016. Copyright (C) 2017 American Chemical Society (ACS). All Rights Reserved.

[432] Ning Xi. Heteroaromatic compounds as pi3 kinase modulators and methods of use, May 15 2014. US Patent App. 14/076,256.

[433] Z. XU, N. Zhang, Q. SUN, and T. WU. Condensed ring derivative, and preparation method, intermediate, pharmaceutical composition and use thereof, September 29 2016. WO Patent App. PCT/CN2016/073,043.

[434] Seiji Yamaguchi, Kazuaki Awajima, Yoshiro Hirai, Hajime Yokoyama, and Shunsaku Shiotani. Furopyridines.XXVIII. reactions of 3-bromo derivatives of furo[2,3-b]-, -[3,2-b]-, -[2,3-c]- and -[3,2-c]pyridine and theirN-oxides. *Journal of Heterocyclic Chemistry*, 35(6):1249–1255, nov 1998.

[435] Seiji Yamaguchi, Masahide Kurosaki, Keiko Orito, Hajime Yokayama, Yoshiro Hirai, and Shunsaku Shiotani. Furopyridines.XXVII. reactions of 2-methyl and 2-cyano derivatives of furo[2,3-b]-, -[3,2-b]-, - [2,3-c]- and -[3,2-c]pyridine. *Journal of Heterocyclic Chemistry*, 35(6):1237–1247, nov 1998.

[436] Rulong Yan, Xiaoni Li, Xiaodong Yang, Xing Kang, Likui Xiang, and Guosheng Huang. A novel one-pot method for the synthesis of substituted furopyridines: iodine-mediated oxidation of enaminones by tandem metal-free cyclization. *Chem. Commun.*, 51(13):2573–2576, 2015.

[437] Youqing Yang, Ruirui Li, Yue Zhao, Dongbing Zhao, and Zhuangzhi Shi. Cu-catalyzed direct c6-arylation of indoles. *Journal of the American Chemical Society*, 138(28):8734–8737, jul 2016.

[438] Zhou Yang, Jian Wang, Xiu-zhi Xu, Chun-ling Zhang, and Zhu-lai Li. Synthesis, characterization and process improvement of 6-substituted pteridinone derivatives. *Jingxi Huagong Zhongjianti*, 42(3):31–34, 43, 2012. Copyright (C) 2017 American Chemical Society (ACS). All Rights Reserved.

[439] S. Yonishi, S. Aoki, Y. Matsushima, and A. Akahane. Pyrazine derivatives and pharmaceutical use thereof, May 6 2005. WO Patent App. PCT/JP2004/016,193.

[440] Summer E. Young, Matthew T. Duvernay, Michael L. Schulte, Craig W. Lindsley, and Heidi E. Hamm. Synthesis of indole derived protease-activated receptor 4 antagonists and characterization in human platelets. *PLoS ONE*, 8(6):e65528, jun 2013.

[441] Tarek Yousfi, Alysha Elliott, Messiad Hanane, Rachid Merdes, and Albert Moyano. Expedient organocatalytic syntheses of 4-substituted pyrazolidines and isoxazolidines. *Molecules*, 21(12):1655, dec 2016.

[442] Chunhui Yu, Xiaoguang Zhang, Jinghua Zhang, and Zhengwu Shen. Total synthesis of dysidavarone a. *Tetrahedron*, 72(29):4337–4345, jul 2016.

[443] H. Yu, M. WEI, Y. Cui, X. Sun, Z. Tong, et al. Egfr inhibitor, and preparation and application thereof, April 14 2016. WO Patent App. PCT/CN2015/091,189.

[444] Jianyu Yuan, Zhichun Zhai, Jing Li, Jialing Lu, Xiaodong Huang, Zhongjie Xu, and Wanli Ma. Correlation between structure and photovoltaic performance of a series of furan bridged donor–acceptor conjugated polymers. *Journal of Materials Chemistry A*, 1(39):12128, 2013.

[445] R. Zahler. Aryloxyacetylindoles and analogs as antibiotic tolerance inhibitors, July 14 2016. WO Patent App. PCT/US2016/012,308.

[446] S.K. Zahn, G. Boehmelt, A. Mantoulidis, U. Reiser, M. Treu, U. Guertler, A. Schoop, F. Solca, U. Tontsch-Grunt, R. Brueckner, et al. 2,4-diamino-pyrimidines used as aurora inhibitors, January 11 2007. WO Patent App. PCT/EP2006/063,736.

[447] Massimo Zambianchi, Andrea Barbieri, Alfredo Ventola, Laura Favaretto, Cristian Bettini, Matteo Galeotti, and Giovanna Barbarella. Testing oligothiophene fluorophores under physiological conditions. preparation and optical characterization of the conjugates of bovine serum albumin with OligothiopheneN-hydroxysuccinimidyl esters. *Bioconjugate Chemistry*, 18(3):1004–1009, may 2007.

[448] C. Zhang, J. Zhang, P.N. Ibrahim, D.R. Artis, R. Bremer, G. Wu, H. Zhu, and M. Nespi. Compounds modulating c-fms and/or c-kit activity and uses therefor, November 13 2008. WO Patent App. PCT/US2007/085,299.

[449] J. Zhang, J. BUELL, K. Chan, P.N. Ibrahim, J. Lin, P. Pham, S. Shi, W. Spevak, G. Wu, and J. Wu. Composés hétérocycliques et leurs utilisations, September 18 2014. WO Patent App. PCT/US2014/029,701.

[450] Ming-Zhi Zhang, Qiong Chen, Nick Mulholland, David Beattie, Dianne Irwin, Yu-Cheng Gu, Guang-Fu Yang, and John Clough. Synthesis and fungicidal activity of novel pimprinine analogues. *European Journal of Medicinal Chemistry*, 53:283–291, jul 2012.

[451] Peilong Zhang, Jiaqiang Dong, Boyu Zhong, Deyi Zhang, Can Jin, Xuejing Meng, Desheng Sun, Xiangyuan Xu, Yong Zhou, Zhi Liang, Minghua Ji, Hailong Li, Tao Xu, Guowei Song, Ling Zhang, Gang Chen, Hongbin Yuan, Joe Shih, Ruihao Zhang, Guojun Hou, Ying Jin, and Qiong Yang. Discovery of 2-arylamino-4-(1-methyl-3-isopropylsulfonyl-4-pyrazol-amino)pyrimidines as potent anaplastic lymphoma kinase (ALK) inhibitors. *Bioorganic & Medicinal Chemistry Letters*, 25(17):3738–3743, sep 2015.

[452] Puwen Zhang, Jeffrey C. Kern, Eugene A. Terefenko, Andrew Fensome, Ray Unwalla, Zhiming Zhang, Jeffrey Cohen, Thomas J. Berrodin, Matthew R. Yudt, and Richard C. Winneker. 7-aryl 1,5-dihydro-benzo[e][1,4]oxazepin-2-ones and analogs as non-steroidal progesterone receptor antagonists. *Bioorganic & Medicinal Chemistry*, 16(13):6589–6600, jul 2008.

[453] Yan-Li Zhao, Lihua Liu, Wenyu Zhang, Chi-Hau Sue, Qiaowei Li, Ognjen Š. Miljanić, Omar M. Yaghi, and J. Fraser Stoddart. Rigid-strut-containing crown ethers and [2]catenanes for incorporation into metal-organic frameworks. *Chemistry - A European Journal*, 15(48):13356–13380, nov 2009.

[454] Ming-Hua Zheng, Wei Sun, Jing-Yi Jin, and Chun-Hua Yan. Molecular keypad locks based on gated photochromism and enhanced fluorescence by protonation effects. *Journal of Fluorescence*, 24(4):1169–1176, apr 2014.

[455] J. Zhou, L.M. Oh, and P. Ma. Convergent synthesis of α-aryl-β-ketonitriles, May 13 2003. US Patent 6,562,965.

[456] Gui-Dong Zhu, Jianchun Gong, Viraj B. Gandhi, Keith Woods, Yan Luo, Xuesong Liu, Ran Guan, Vered Klinghofer, Eric F. Johnson, Vincent S. Stoll, Mulugeta Mamo, Qun Li, Saul H. Rosenberg, and Vincent L. Giranda. Design and synthesis of pyridine–pyrazolopyridine-based inhibitors of protein kinase b/akt. *Bioorganic & Medicinal Chemistry*, 15(6):2441–2452, mar 2007.

[457] Xiaohua Zhu, Abdelbasset A. Farahat, Meena Mattamana, April Joice, Trupti Pandharkar, Elizabeth Holt, Moloy Banerjee, Jamie L. Gragg, Laixing Hu, Arvind Kumar, Sihyung Yang, Michael Zhuo Wang, David W. Boykin, and Karl A. Werbovetz. Synthesis and pharmacological evaluation of mono-arylimidamides as antileishmanial agents. *Bioorganic & Medicinal Chemistry Letters*, 26(10):2551–2556, may 2016.

[458] F. Ziemer, L. Willms, C. Rosinger, T. Auler, E. Hacker, and U. Bickers. Pyridinecarboxamides, useful-plant-protecting composition comprising them and processes for their prepartion and their use, October 30 2008. US Patent App. 12/111,419.

[459] T. Zierke, J. Rheinheimer, M. Rack, S.P. Smidt, A.G. Altenhoff, J. Schmidt-Leithoff, and N. Challand. Method for the production of n-substituted (3-dihalomethyl-1-methyl-pyrazole-4-yl) carboxamides, December 4 2008. WO Patent App. PCT/EP2008/056,712.

[460] Eli Zysman-Colman, Karla Arias, and Jay S. Siegel. Synthesis of arylbromides from arenes andN-bromosuccinimide (NBS) in acetonitrile — a convenient method for aromatic bromination. *Canadian Journal of Chemistry*, 87(2):440–447, feb 2009.
